# Breastfeeding promotion interventions and breastfeeding practices: a systematic review

**DOI:** 10.1186/1471-2458-13-S3-S20

**Published:** 2013-09-17

**Authors:** Sarah Haroon, Jai K Das, Rehana A Salam, Aamer Imdad, Zulfiqar A Bhutta

**Affiliations:** 1 Division of Women & Child Health, The Aga Khan University, Karachi 74800, Pakistan; 2 Global Child Health and Policy, Centre for Global Child Health, The Hospital for Sick Children, Toronto, ON, Canada

## Abstract

**Introduction:**

Exclusive Breastfeeding (EBF) rates remain low in both low-income and high-income countries despite World Health Organization recommendations for EBF till 6 months. Breastfeeding has been shown to have a protective effect against gastrointestinal infections, among other benefits. Large-scale interventions focusing on educating mothers about breastfeeding have the potential to increase breastfeeding prevalence, especially EBF, up to recommended standards and also to decrease infant morbidity.

**Methods:**

A systematic literature search was conducted for RCTs and quasi-experimental studies comparing breastfeeding education or support to routine care. The effect of interventions was observed for exclusive, predominant, partial and no breastfeeding rates. The time intervals of interest were day 1, <1 month, and 1 to 5 months. Outcome-specific evidence was graded according to the Child Health Epidemiology Reference Group (CHERG) rules using the adapted Grading of Recommendations, Assessment, Development and Evaluation (GRADE) criteria and recommendations were made from studies in developing countries for inclusion into the Lives Saved Tool (LiST) model.

**Results:**

After reviewing 4600 abstracts, 372 studies were selected for full text screening and 110 of these studies were finally included. Statistically significant increases in EBF rates as a result of breastfeeding promotion interventions were observed: 43% at day 1, 30% at <1 month, and 90% at 1-5 months. Rates of ‘no breastfeeding’ reduced by 32% at 1 day, 30% at <1 month, and 18% at 1-5 months. The effect of interventions on the rates of predominant and partial breastfeeding were non-significant.

**Conclusion:**

Breastfeeding education and/or support increased EBF rates and decreased no breastfeeding rates at birth, <1 month and 1-5 months. Combined individual and group counseling appeared to be superior to individual or group counseling alone. Interventions in developing countries had a greater impact than those in developed countries.

## Introduction

The World Health Organization (WHO) recommends exclusive breastfeeding (EBF) to infants till 6 months of age to achieve optimum growth [1]. Despite this, EBF remains uncommon in most countries, even in countries with high rates of breastfeeding initiation [2,3]. In the developing world, one out of every three children is exclusively breastfed for the first six months of life, though considerable variation exists across regions [4]. Recent data shows that the prevalence of EBF in developing countries has increased from 33% in 1995 to just 39% in 2010 [5]. These figures were based on 66 countries covering 74% of the developing world population. The prevalence of EBF increased in almost all regions in the developing world, with a major improvement seen in West and Central Africa where the prevalence doubled from 12% to 28%, while more modest improvements were observed in South Asia where the increase was from 40% in 1995 to 45% in 2010. A recent WHO report shows that the median coverage of EBF has increased from 26% in 2000-2005 to 40% in 2006-2011 in the 48 Countdown countries [6].

EBF has protective effects against gastrointestinal infection [1] and the high incidence of morbidity and mortality from gastrointestinal infection in developing countries demands large-scale interventions to increase breastfeeding prevalence and exclusivity as evidence shows that “no breast feeding” is associated with a significant 165% increase in diarrhoea incidence in 0-5 month old infants and a 32% increase in 6-11 month old infants [7].

Lack of knowledge and confidence were found as the main reasons among mothers for less than optimum breastfeeding duration [8,9]. Perception of insufficient milk and work outside the home were cited as common reasons for premature weaning or not breast-feeding exclusively [10,11]. Pediatricians, nurses, midwives and lay counselors should therefore actively promote and educate, while taking into account mothers’ situational limitations.

Strategies that have been successful in increasing breastfeeding rates are the Baby Friendly Hospital Initiative (BFHI) [12], and the use of peer counselors in settings where home deliveries are predominant [13,14]. Large-scale interventions including the Integrated Management of Childhood Illness (IMCI) program in developing countries, which have shown to improve feeding practices and reduced growth faltering [15,16]. Other strategies that have been employed to increase education include mother-to-mother support and contact with lay counselors or trained personnel via home visits [11,17] or telephone-based support [10,18]. These interventions may be carried out in a one-to-one counseling session or may occur in a group setting [19-22] or peer support groups [11]. Apart from interactive counseling strategies, large-scale awareness programs have also been launched via mass, electronic and print media.

Several reviews on the effect of educational interventions to increase breastfeeding have been conducted. A review by Chapman et al [23] found that peer counselors effectively improved breastfeeding initiation, duration and exclusivity. A recent Cochrane review by Lumbiganon et al [24] found that peer counseling, lactation consultation and formal BF education during pregnancy increased BF duration. The review conducted by Imdad et al [25] concludes that EBF rates rose significantly as a result of educational interventions, with a greater effect observed in developing countries. We in this review have updated the previous review by Imdad et al [25] to include the studies published after the last search date and have in addition expanded it to examine the effect of these interventions beyond EBF to include predominant, partial and no breastfeeding rates. We have reviewed and evaluated the quality of included studies according to the Child Health Epidemiology Reference Group (CHERG) adaptation of Grading of Recommendations, Assessments, Development and Education (GRADE) criteria [26].

## Methods

We searched published literature from PubMed, Medline, Cochrane Library, EMBASE and WHO regional databases to identify studies examining the effects of interventions to promote breastfeeding on breastfeeding rates; exclusive, predominant, partial or no breastfeeding. We used the Medical Subject Heading (MeSH) Terms and keywords in various combinations. No language or date restrictions were employed in the electronic searches. Two authors independently assessed the eligibility using pre-defined inclusion and exclusion criteria and performed data extraction. Any discrepancies between the reviewers in either the decision of inclusion or exclusion of studies or in data extraction were resolved by discussion aimed at reaching consensus. If two or more studies presented data for the same population during the same time period, the most applicable study based on methods and analysis was included in the meta-analyses.

### Inclusion criteria

We selected studies that were either randomized controlled trials (RCTs) or quasi-experimental trials. Studies with community- or facility-based interventions were included. The type of interventions included were those that offered education and/or support given to mothers through counselors (lay counselors and health professionals), and in either individual or group sessions, or a combination of both. All studies where intervention (education/support) was given either in prenatal, postnatal, or combined prenatal and postnatal periods, were included. Studies were included irrespective of the mode of delivery, whether vaginal or cesarean. For non-English articles, we primarily relied on the abstracts but did not translate the entire article into English. If the desired outcome was not present in the abstract, the study was excluded.

### Exclusion criteria

We excluded studies that had before-after study designs, or were cohort and cross-sectional studies. All studies in which interventions were given specifically to preterm/very preterm babies, low birth weight/very low birth weight babies, babies with prenatal disease, born to drug-using mothers or babies in the Neonatal Intensive Care Unit (NICU) were excluded. Other interventions for promotion of breastfeeding like skin-to-skin contact or delayed pacifier use with the goal of decreasing ambivalence and resistance toward sustained breastfeeding were excluded.

### Abstraction, analysis and summary measure

For the studies that met the final inclusion criteria, double data abstraction was done describing study identifiers and context, study design and limitations, intervention specifics and outcome effects into a standardized abstraction form as detailed in the CHERG Systematic Review Guidelines [26]. Each study was assessed and graded according to the CHERG adaptation of the GRADE technique [27].

### Quantitative data synthesis

For any outcome with more than one study, we conducted a meta-analysis using Revman 5.2 [28] and reported the Mantel-Haenszel pooled relative risk (RR) and corresponding 95% confidence interval (CI). Heterogeneity was assessed by a low P value (less than 0.1) or a large chi-squared statistic relative to its degree of freedom. The I^2^ values were also examined, and a value greater than 50% was interpreted as representing substantial and high heterogeneity, where causes were explored and the random effects model used.

Subgroup analyses were also done for studies; group vs. individual counseling, community based interventions vs. facility based interventions, and developing countries vs. developed countries. We summarized the evidence by outcome, including qualitative assessments of study quality and quantitative measures, according to the standard guidelines. A grade of “high”, “moderate”, “low” and “very low” was used for grading the overall evidence indicating the strength of an effect on specific health outcome according to the CHERG Rules for Evidence Review [26].

### Outcomes and definitions

We have specified breastfeeding outcomes according to the categories of breastfeeding defined by the WHO [29,30]. The outcomes of interest included ‘EBF’, ‘Predominant breastfeeding’, ‘Partial breastfeeding’ and ‘No breastfeeding’ rates at day 1, <1 month and 1-5 months age.

‘EBF’ was defined as the child receiving only breast milk (including milk expressed or from a wet nurse) and no other type of milk or solids but could include vitamins, drops of other medicines and oral rehydration therapy (ORT). ‘Predominant breastfeeding’ was defined as the infant having breast milk as the predominant source of nourishment; however, the infant may also have received liquids (water and water-based drinks, fruit juice), ritual fluids and ORT, drops or syrups (vitamins, minerals and medicines). ‘Partial breastfeeding’ was defined as giving a baby some breastfeeds, and some artificial feeds, either milk or cereal, or other food. ‘No breastfeeding’ was defined as infants receiving no breast milk at all.

The time intervals of interest; day 1, <1 month and 1-5 months, were selected. “Day 1” was intended to refer to the early postpartum period and was extended to include time at hospital discharge provided it occurred at approximately the routine 48-hours postpartum. Where more than one data point was presented during this time period in a study, the earlier one was selected. The time interval “<1 month” included the time beginning from the end of the early postpartum period to 30 days. The time interval “1-5 months” included the beginning of the 2^nd^ month to the end of 6 months. Breastfeeding rates after 6 months were recorded if they were reported in studies. For each of these time intervals, when multiple data for outcomes belonging to the same time interval were presented, the later data point was selected.

We performed subgroup analyses based on the types of counseling. “Individual counseling” was defined as interventions which solely had individual counseling and included one-on-one education or social support via home visits or telephone support. “Group counseling”, was defined as interventions with solely group counseling, including education or support sessions, discussions or classes in groups directed at mothers or other family members. “Individual and group counseling” included those studies that used interventions involving both individual and group counseling.

Subgroup analyses were also done based on the level of care. “Community-based interventions” included studies that had interventions conducted solely at the community level, in the form of care given at the home or in community and village centers, or disbursed throughout the community as an awareness program. “Facility-based interventions” was defined as interventions conducted solely at the facility level, including hospitals (such as the BFHI) and outpatient clinics or involving follow-up with facility-based professionals in the form of telephone calls. “Facility- and community-based interventions” included those studies that used interventions that were conducted at both the facility and community level.

Subgroup analyses were also conducted based on the country in which the intervention took place. The World Bank list of economies was used to classify developing (low-income and middle-income) and developed (high-income) countries [31].

## Results

**Figure 1:**
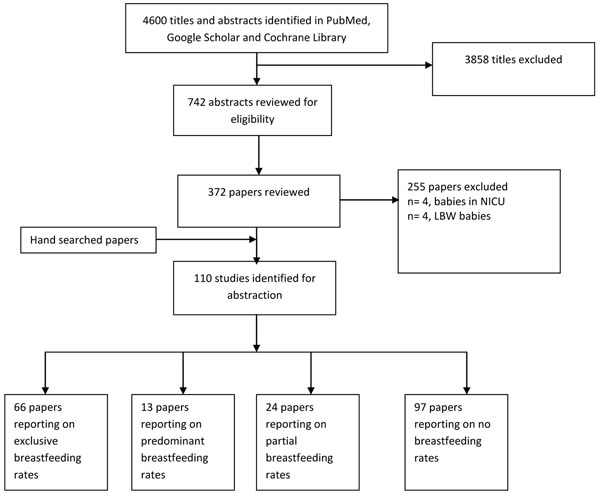
Search strategy flow diagram

We conducted the search on October 5, 2012 and updated it on November 27, 2012. We screened 4600 titles and abstracts identified through literature searches and contacts with subject area experts. Of these, we reviewed 372 papers and included 110 in our final database (Figure 1). These studies included 63 RCTs and 47 quasi-experimental studies. Of these studies, 78 had individual counseling, 14 had group counseling, and 19 studies had both individual and group counseling. 21 of these studies were done in community, 46 in facility while 43 were both community- and facility-based. 34 studies were conducted in developing countries and 76 in developed countries.

Various educational interventions were employed by the included studies; counseling during home visits was used in 39 studies, peer counseling in 18 studies and peer support groups in three studies. 30 studies used telephonic counseling and two studies used Internet or software-based educational programs. 16 studies used formal educational classes and 15 studies used in-hospital counseling. There were three studies that used counseling of fathers as the primary intervention.

### Exclusive breastfeeding rates

**Figure 2:**
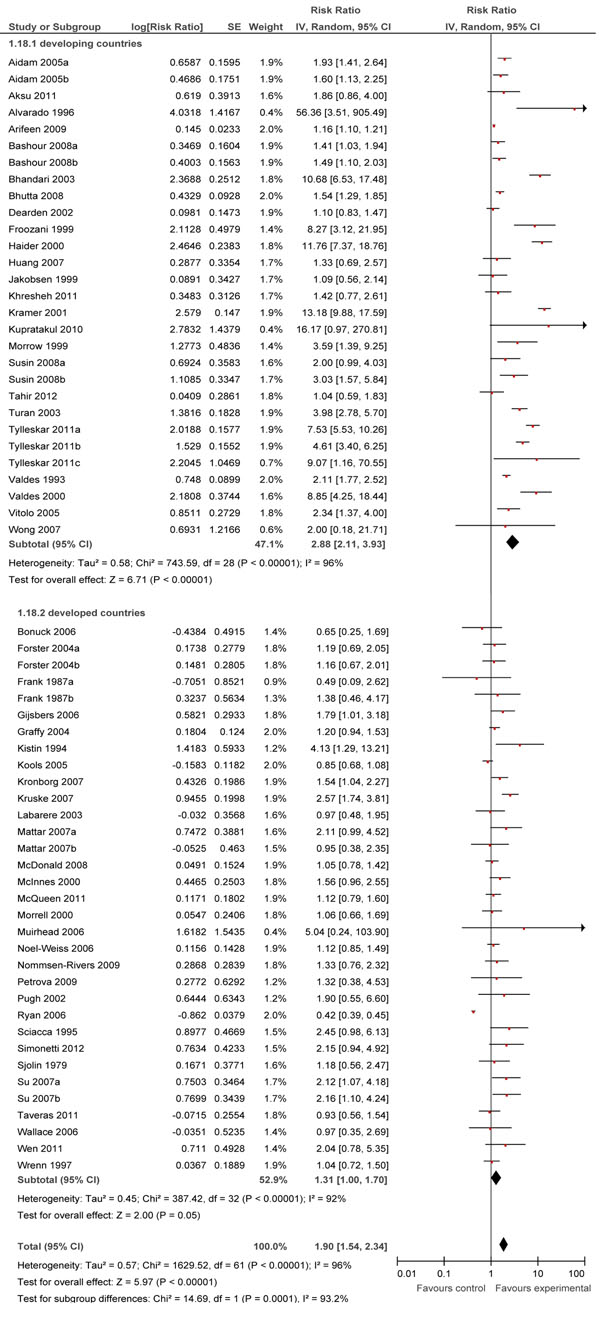
Effect of breastfeeding education on the rate of exclusive breastfeeding for 1 to 5 months

In Table 1, we report the quality assessment of breastfeeding promotion intervention on EBF. A total of 66 studies were included for this outcome [11-14,17-22,32-87]. 27 of 66 studies were conducted in developing countries. Overall, educational interventions significantly increased EBF rates at day 1 by 43% (RR: 1.43, 95% CI: 1.09-1.87), at <1 month by 30% (RR: 1.30, 95% CI: 1.19-1.42) and at 1-5 months by 90% (RR: 1.90, 95% CI: 1.54-2.34) (Figure 2).

**Table 1:** Summary of findings for the effect of breastfeeding promotion interventions on exclusive breastfeeding rates.

Quality Assessment						Summary of Findings			
				**Directness**		**No of events**			

**No of Studies**	**Design**	**Limitations**	**Consistency**	**Generalizability to population of interest**	**Generalizability to intervention of interest**	**Intervention**	**Control**	**Relative Risk (95% CI)**	**Comments**

***Rate of exclusive breastfeeding at day 1: low outcome-specific quality***									

15	6 RCTs [14,40,49,70,71,74], 9 QE [21,32,45,48,56,59,64,85,88]	Studies used different follow up periods and recall criteria. Mothers in the intervention group may have over-reported feeding practices.	6 of 15 studies suggest benefit. Significant heterogeneity	10 out of 15 studies were conducted in developed countries	Pooled results for different types of interventions.	4093	6316	1.43 [1.09, 1.87]	Random effects meta-analysis due to heterogeneity. The majority of studies used individual counseling as the intervention; most were facility and community-based.

**Rate of exclusive breastfeeding at 0-1 month*****: Low outcome-specific quality***									

30	22 RCTs, 8 QE [13,17-19,21,22,33,38,39,41,42,45,52,53,55-57,60,61,65,66,68,71-74,76,81,84,85]	Studies used different follow up periods. Recall criteria variable across studies (past 24 hr, past week or previous month). Mothers in the intervention group may have over-reported feeding practices.	15 of 30 studies suggest benefit. Significant heterogeneity	19 of 30 studies were conducted in developed countries	Pooled results for different types of interventions	1512	1276	1.30 [1.19, 1.42]	Random effects meta-analysis due to heterogeneity. The majority of studies used individual counseling as the intervention; most were facility and community-based.

**Rate of exclusive breastfeeding at 1-6 months*****: low outcome-specific quality***									

53	34 RCTs, 19 QE [11-14,19,20,22,33-38,40-52,54,57-59,61-65,67,69,71-83,85-87]	Variable follow up periods used in studies. Recall criteria variable across studies (past 24 hr, past week or previous month). Mothers in the intervention group may have over-reported feeding practices.	21 of 53 studies suggest benefit. Significant heterogeneity	29 of 53 studies were conducted in developed countries	Pooled results for different types of interventions	5481	4897	1.90 [1.54, 2.34]	Random effects meta-analysis due to heterogeneity. The majority of studies used individual counseling as the intervention; most were facility and community-based

At day 1, subgroup analyses showed that individual counseling alone led to a 60% increase (RR: 1.60, 95% CI: 1.04-2.48) while the effects of group counseling alone or combined individual and group counseling were non-significant. Subgroup analyses for the level of care showed that results were significant only for facility-based interventions. In developing countries, these interventions led to an increase of 157% (RR: 2.57, 95%CI: 1.39-4.77) whereas a non-significant effect was demonstrated in developed countries.

For the <1 month interval, subgroup analyses showed that the effects of individual counseling and combined individual and group counseling were significant, with increases of 31% and 27% respectively. Facility-based interventions were found to increase EBF rates significantly by 26% (RR: 1.26, 95% CI: 1.11-1.43) and combined facility and community based interventions showed significant increase of 31% (RR: 1.31, 95%CI: 1.14-1.50). The effects were significant for both developing and developed countries at 35% (RR: 1.35, 95% CI: 1.15-1.58) and 26% (RR: 1.26, 95% CI: 1.13-1.41) respectively.

At 1-5 months, subgroup analyses showed that both individual and group counseling alone had significant impacts at 90% (RR: 1.90, 95% CI: 1.54-2.34) and 80% (RR: 1.80, 95% CI: 1.18-2.74), respectively. Combined individual and group counseling led to an increase of 101% (RR: 2.01, 95% CI: 1.43-2.82), Subgroup analyses for level of care revealed that both community and facility-based care had significant results at 159% (RR: 2.59, 95% CI: 1.80-3.73) and 87% (RR: 1.87, 95% CI: 1.26-2.78) respectively and the effect of combined facility- and community-based care was an increase of 47% (RR: 1.47, 95% CI: 1.08-1.99). Interventions in developing countries led to a significant increase of 188% (RR: 2.88, 95% CI 2.11-3.93), while the impact was non-significant for developed countries.

### Predominant breastfeeding rates

**Figure 3:**
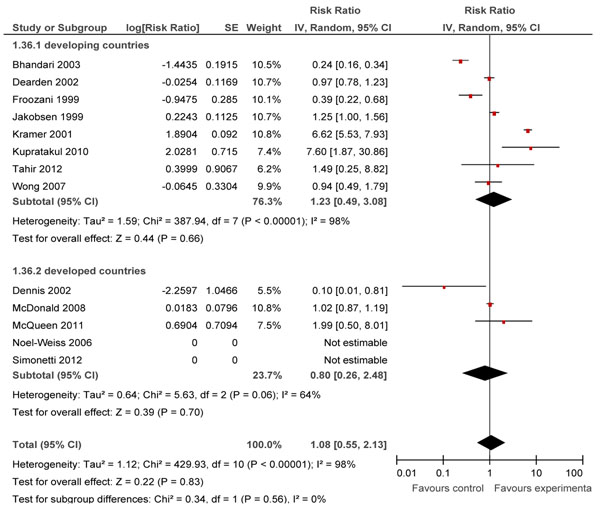
Effect of breastfeeding education on the rate of predominant breastfeeding for 1-5 months

In Table 2, we report the quality assessment of breastfeeding promotion intervention on predominant breast feeding. 13 studies had reported this outcome [11,12,18,20,36,42,46,52,58,61,72,76,85] and eight of these were conducted in developing countries. Overall, educational interventions had a non-significant effect on predominant breastfeeding rates at <1 month (RR: 0.66, 95% CI: 0.43, 1.01) and at 1-5 months (RR: 1.08, 95%CI: 0.55, 2.13) (Figure 3), while there were no studies reporting predominant breastfeeding rates at day 1. Subgroup analysis also did not show significant findings for any of the subgroups.

**Table 2:** Summary of findings for the effect of breastfeeding promotion interventions on predominant and partial breastfeeding rates.

Quality Assessment						Summary of Findings			
				**Directness**		**No of events**			

**No of studies**	**Design**	**Limitations**	**Consistency**	**Generalizability to population of interest**	**Generalizability to intervention of interest**	**Intervention**	**Control**	**Relative Risk (95% CI)**	**Comments**

**Rate of predominant breastfeeding at <1 month*****: Moderate outcome-specific quality***									

6	5 RCTs, 1 QE [18,52,61,72,76,85]	Variable follow up periods used in studies. Recall criteria variable across studies (past 24 hr, past week or previous month). mothers in the intervention group may have over-reported feeding practices	None of the studies suggest benefit. Insignificant heterogeneity	3 studies were conducted in developing countries	Pooled results for different types of interventions	33	59	0.66 [0.43, 1.01]	Fixed effects meta-analysis; insignificant heterogeneity. Most studies were facility-based and all used individual counseling.

**Rate of predominant breastfeeding at 1- 5 months*****: low outcome specific-quality***									

13	10 RCTs, 3 QE [11,12,18,20,36,42,46,52,58,61,72,76,85]	Variable follow up periods used in studies. Recall criteria variable across studies (past 24 hr, past week or previous month). mothers in the intervention group may have over-reported feeding practices	2 studies suggest benefit. Significant heterogeneity	8 of 13 studies were conducted in developed countries	Pooled results for different types of interventions	1433	707	1.08 [0.55, 2.13]	Random effects meta-analysis due to significant heterogeneity. Most studies are facility-based and used individual counseling.

**Rate of partial breastfeeding at day 1*****: low outcome-specific quality***									

6	2 RCT, 4 QE [45,49,59,64,71,89]	Variable follow up periods used in studies. Recall criteria variable across studies (past 24 hr, past week or previous month). Mothers in the intervention group may have over-reported feeding practices.	1 of 6 studies suggests benefit. Significant heterogeneity	1 of 6 studies was conducted in a developing country	Pooled results for different types of interventions	101	99	1.21 [0.79, 1.87]	Random effects meta-analysis due to significant heterogeneity. Most studies used individual counseling and most were facility-based.

**Rate of partial breastfeeding at <1 month*****: moderate outcome-specific quality***									

11	8 RCTs, 3 QE [18,19,45,52,61,66,71,72,76,85,88]	Variable follow up periods used in studies. Recall criteria variable across studies (past 24 hr, past week or previous month). mothers in the intervention group may have over-reported feeding practices.	None of the studies suggest benefit. Insignificant heterogeneity	5 of 11 studies were conducted in developing countries	Pooled results for different types of interventions	112	151	0.88 [0.72, 1.08]	Fixed effects meta-analysis; insignificant heterogeneity Most studies used individual counseling and most were facility-based.

**Rate of partial breastfeeding at 1-5 months*****: moderate outcome-specific quality***									

20	11 RCTs, 9 QE [18-20,36,42,45,47,49,51,52,59,61,62,71,72,76,80,85,86,89]	Variable follow up periods used in studies. Recall criteria variable across studies (past 24 hr, past week or previous month). mothers in the intervention group may have over-reported feeding practices.	None of the studies suggest benefit. Significant heterogeneity	9 of 20 studies were conducted in developing countries	Pooled results for different types of interventions	524	578	0.87 [0.75, 1.02]	Random effects meta-analysis due to significant heterogeneity. Most studies used individual counseling and most were facility-based.

### Partial breastfeeding rates

**Figure 4:**
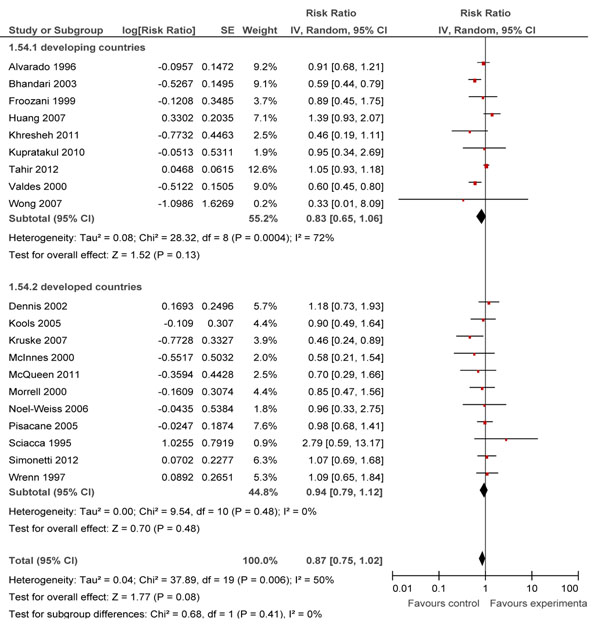
Effect of breastfeeding education on the rate of partial breastfeeding for 1 to 5 months

In Table 2, we report the quality assessment of breastfeeding promotion intervention on partial breast feeding. 24 studies [18-20,34,36,42,45,47,49,51,52,59,61,62,64,66,71,72,76,80,85,86,88,89] reported outcomes of partial breastfeeding, of which ten were conducted in developing countries. Overall, educational interventions had a non-significant effect on partial breastfeeding rates at day 1 (RR: 1.21 95% CI: 0.79, 1.87), at <1 month (RR: 0.88 95% CI: 0.72, 1.08) and at 1-5 months (RR: 0.87, 95%CI: 0.75, 1.02) intervals (Figure 4).

For the subgroup analysis based on the level of care, combined facility and community-based interventions had a significant reduction of 66% (RR: 0.34, 95% CI: 0.13-0.93) at <1 month duration. Findings from all other subgroups were non-significant.

### ‘No breastfeeding’ rates

**Figure 5:**
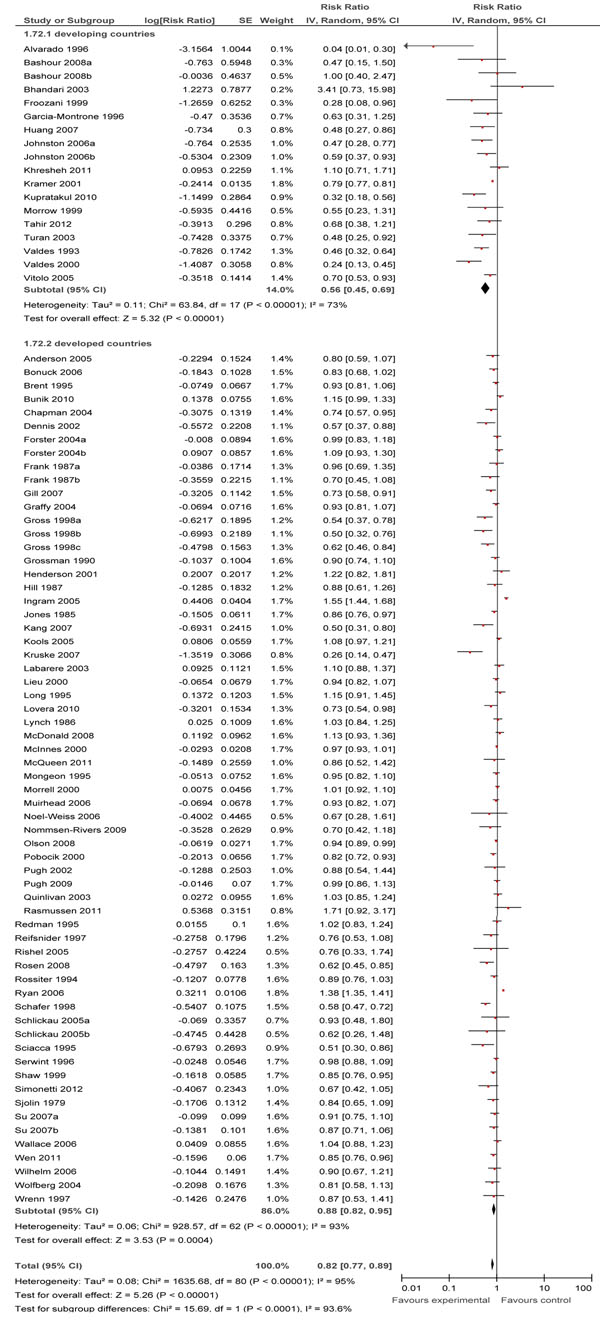
Effect of breastfeeding education on the rate of no breastfeeding for 1 to 5 months

In Table 3, we report the quality assessment of breastfeeding promotion intervention on ‘no breast feeding’. Of the 97 papers [10-14,17-20,33,35-38,40-42,44,45,47,49,51-55,57-74,76-84,86,88-130] reporting this particular outcome, 23 were from developing countries. Overall, educational interventions significantly decreased rates of no breastfeeding by 32% at day 1 (RR: 0.68, 95% CI: 0.54-0.87), 30% (RR: 0.70, 95% CI: 0.62-0.80) at <1 month and 18% (RR: 0.82, 95% CI: 0.77-0.89) at 1-5 months intervals (Figure 5).

**Table 3:** Summary of findings for effect of breastfeeding promotion interventions on ‘no breastfeeding’ rates.

Quality Assessment						Summary of Findings			
				**Directness**		**No of events**			

**No of Studies**	**Design**	**Limitations**	**Consistency**	**Generalizability to population of interest**	**Generalizability to intervention of interest**	**Intervention**	**Control**	**Relative Risk (95% CI)**	**Comments**

**Rate of no breastfeeding at day 1*****: low outcome-specific quality***									

38	21 RCTs, 17 QE [11,13,14,17,33,36,37,44,49,59,63,64,68-71,74,78,79,89-91,96,100,103,107,108,110,111,113,117,120-122,124-127]	Variable follow up periods used in studies. Recall criteria variable across studies (past 24 hr, past week or previous month).	Most studies suggest benefit. Significant heterogeneity	10 of 38 studies were conducted in developing countries	Pooled results for different types of interventions	48026	39843	0.68 [0.54, 0.87]	Random effects meta-analysis due to significant heterogeneity Most studies used individual counseling and most were facility and community-based. Effect of benefit refers to decrease in numbers not breastfeeding.

**Rate of no breastfeeding at <1 month*****: low outcome-specific quality***									

33	21 RCTs, 12 QE [10,18,19,38,45,52,53,61,63,66,68,71-74,76,88,90,91,93,95,98,99,101-103,106,113,117,118,120,124,128]	Variable follow up periods used in studies. Recall criteria variable across studies (past 24 hr, past week or previous month).	10 of 33 studies suggest benefit. Significant heterogeneity	4 of 33 studies were conducted in developing countries	Pooled results for different types of interventions	770	1018	0.70 [0.62, 0.80]	Random effects meta-analysis due to significant heterogeneity. Most studies used individual counseling.

**Rate of no breastfeeding at 1-5 months*****: low outcome-specific quality***									

73	41 RCTs, 32 QE [10,12,13,17-20,35,36,38,40-42,44,45,47,49,51,52,54,58,59,61-64,67-69,71-74,76,78-83,86,90-93,95-99,101,103-106,108-110,112-121,123,124,126,129,130]	Variable follow up periods used in studies. Recall criteria variable across studies (past 24 hr, past week or previous month).	25 of 73 studies suggest benefit. Significant heterogeneity	16 of 73 studies were conducted in developing countries	Pooled results for different types of interventions	15473	17578	0.82 [0.77, 0.89]	Random effects meta-analysis due to significant heterogeneity Most studies used individual counseling.

At day 1, subgroup analyses for type of counseling revealed that group counseling alone resulted in a 43% reduction (RR: 0.57, 95% CI: 0.41-0.80) and individual counseling alone led to a 27% reduction (RR: 0.73, 95% CI: 0.55-0.96). The effect of combined individual and group counseling was non-significant. Only facility-based interventions led to a significant reduction of 52% (RR: 0.48, 95% CI: 0.34-0.69); the effects of community-based and combined facility- and community-based interventions were non-significant. Interventions in both developing and developed countries had significant results, with reduction of 42% (RR: 0.58, 95% CI: 0.44-0.78) and 27% (RR: 0.73, 95% CI: 0.57-0.95), respectively.

At <1 month, subgroup analyses for type of counseling showed that combined individual and group counseling resulted in a 34% decrease (RR: 0.66, 95% CI: 0.51-0.87), individual counseling alone resulted in a 29% decrease (RR: 0.71, 95% CI: 0.61-0.84) and group counseling alone led to a 29% decrease (RR: 0.71, 95% CI: 0.51-0.99) . In the subgroup analyses for level of care, the effects of facility-based interventions and combined facility- and community-based interventions were 32% (RR: 0.68, 95% CI: 0.56-0.83) and 33% (RR: 0.67, 95% CI: 0.54-0.83) respectively. The effects of community-based interventions were non-significant. Developing countries showed a reduction of 49% (RR: 0.51, 95% CI: 0.29-0.90) and in developed countries there was a reduction of 29% (RR: 0.71, 95% CI: 0.62-0.81).

For the 1-5 months interval, subgroup analyses showed statistically significant reduction in ‘no breastfeeding’ rates for combined individual and group counseling with a reduction of 32% (RR: 0.68, 95% CI: 0.50-0.92), individual counseling alone with a reduction of 14% (RR: 0.86, 95% CI: 0.79-0.94), and group counseling alone with a reduction of 24% (RR: 0.76, 95% CI: 0.63-0.91). Facility-based and combined facility- and community-based interventions led to significant reduction of 18% (RR: 0.82, 95% CI: 0.75-0.89) and 17% (RR: 0.83, 95% CI: 0.75-0.93), respectively; however, results for community based interventions were non-significant. The effect of educational interventions in both developing and developed countries were significant at 44% (RR: 0.56, 95% CI: 0.45-0.69) and 12% (RR: 0.88, 95% CI: 0.82-0.95) respectively.

### Beyond 6 months

Beyond 6 months, data was available from 11 studies [12,34,38,81,83,89,94,98,104,108,113] for exclusive, partial and no breastfeeding rates. At 6-12 months, a 19% increase in partial breastfeeding rates was demonstrated, which was significant (RR: 1.19, 95% CI: 1.12-1.26). The effect of interventions was non-significant for both exclusive and no breastfeeding rates.

### Recommendations for LiST model

Considering only the estimates from studies conducted in developing countries (Table 4), we propose that educational interventions increase EBF rates at day 1 by 157% (RR: 2.57 95% CI: 1.39, 4.77), at <1 month by 35% (RR: 1.35, 95% CI: 1.15, 1.58) and at 1-5 months by 188% (RR: 2.88, 95% CI: 2.11, 3.93). For predominant and partial breastfeeding rates, results were non-significant for all age durations. For ‘no breastfeeding’ we propose that educational interventions are associated with a reduction of 42% (RR: 0.58, 95% CI: 0.44, 0.78) at day 1, 49% (RR: 0.51, 95% CI: 0.29, 0.90) for <1 month and 44% (RR: 0.56, 95% CI: 0.45, 0.69) for 1-5 month ages.

**Table 4:** Estimates of effect of breastfeeding promotion interventions on exclusive, predominant, partial and no breastfeeding rates in developing countries: Recommendations for LiST model.

Feeding practice and time interval	Relative Risk (95% CI)
Exclusive breastfeeding rate at day 1	2.57 [1.39, 4.77]
Exclusive breastfeeding rate at <1 month	1.35 [1.15, 1.58]
Exclusive breastfeeding rate at 1-5 months	2.88 [2.11, 3.93]
Predominant breastfeeding rate at <1 month	0.67 [0.42, 1.06]
Predominant breastfeeding rate at 1-5 months	1.23 [0.49, 3.08]
Partial breastfeeding rate at day 1	0.84 [0.61, 1.15]
Partial breastfeeding rate at <1 month	0.94 [0.72, 1.24]
Partial breastfeeding rate at 1-5 months	0.83 [0.65, 1.06]
‘No breastfeeding’ rate at day 1	0.58 [0.44, 0.78]
‘No breastfeeding’ rate at <1 month	0.51 [0.29, 0.90]
‘No breastfeeding’ rate at 1-5 months	0.56 [0.45, 0.69]

## Discussion

In this systematic review we summarized the effect of educational interventions to promote breastfeeding. We specifically examined the effect of these interventions on the various categories of breastfeeding, i.e. exclusive, predominant, partial breastfeeding and no breastfeeding, at day 1, <1 month, and 1-5 months. We also observed the prevalence of breastfeeding beyond 6 months if any study had reported outcomes in this age range.

EBF rates appeared to increase as a result of breastfeeding promotion interventions by 43% at day 1, by 30% till 1 month, and by 90% from 1-5 months (low outcome-specific quality of evidence). Significant reduction in rates of no breastfeeding were observed for the same time intervals, i.e. by 32% at day 1, by 30% till 1 month, and by 18% from 1-5 months (low outcome-specific quality of evidence). The overall effects of these interventions on predominant and partial breastfeeding rates were non-significant (moderate outcome-specific quality of evidence).

Combined individual and group counseling was found more effective than individual or group counseling alone. Overall, facility and combined facility- and community-based interventions led to greater improvements in breastfeeding rates, except for EBF at 1-5 months when the greatest increase resulted from community-based interventions. The effects of interventions in developing countries were greater than those observed in developed countries, i.e. increases in EBF rates of 35% compared to 26% at <1 month. At day 1 and at 1-5 months, the effects of interventions in developing countries on EBF rates were increases of 157% and 188%, respectively, whereas results for developed countries were non-significant. Reduction in ‘no breastfeeding’ rates of 42% were demonstrated in developing countries compared to 27% in developed countries at day 1, 49% compared to 29% at <1 month, and 44% compared to 12% at 1-5 months. Beyond 6 months, educational interventions had no significant effect except increasing rates of partial breastfeeding by 19% (moderate outcome-specific quality of evidence).

One of the limitations of this review is that the methodology of the RCTs included, indicated an unclear or high risk of bias as most RCTs demonstrated unclear blinding and/or allocation concealment. As quasi-experimental trials were also included, most of which did not employ blinding, this limited the quality of the evidence. Not only was there methodological heterogeneity across studies based on study design, clinical heterogeneity was also observed due to variations in types of intervention and the duration of the intervention, target population (differences in income and education), outcome definitions (‘fully’ breastfeeding interpreted as EBF but possibly including predominant BF) and different time intervals for follow-up. There were also differences in exposure to intervention, e.g. in the availability of a breastfeeding support telephone service, all the mothers in the intervention group did not choose to use the service. To investigate the subsequent statistical heterogeneity, we performed subgroup analyses to identify the cause. The random effects model was used to address this heterogeneity when it could not be explained. Criteria for recall of infant feeding practices for mothers were also variable, e.g. ranging from ‘continuous EBF from birth’ to ‘EBF in last 24 hours’.

Other reviews on the subject include a Cochrane review on antenatal education for increasing breastfeeding duration, which examined specific types of breastfeeding education and compared multiple methods with a single method of education. Peer counseling, lactation consultation and formal BF education during pregnancy were found to increase BF duration. Though we have included interventions given both during the antenatal and postpartum periods, our findings are similar with respect to the effectiveness of individual and group counseling, or individual counseling alone. Our findings are also similar to the previous review [25], which concluded that educational interventions increased EBF rates at 4-6 weeks and at 6 months, and the review by Chapman et al [23], which specifically examined studies with peer counseling programs and found that in the majority of studies peer counselors improved rates of breastfeeding initiation, duration and exclusivity.

We observed a statistically significant increase in EBF rates as well as a reduction in no breastfeeding rates at all measured time intervals till 6 months of age as a result of promotional interventions for breastfeeding. This corresponds with the messages in many interventions, which promote EBF till the age of 6 months in compliance with WHO recommendations [1]. A general effect of reduction of predominant and partial breastfeeding rates was demonstrated at day 1, <1 month and 1-5 months, however results were broadly non-significant. This finding may be explained by the rise of EBF as mothers realize the importance of not introducing formula or non-nutritional water-based foods early in the life of the infant. One exception to this pattern was the significant increase in partial breastfeeding rates of 325% (RR 4.25, CI 1.43-12.61) in the community-based interventions subgroup at day 1, comprising a single quasi-experimental study [59].

The impact was greater in developing countries when compared to developed countries. This could be because in less developed health systems, routine breastfeeding education in-hospital or follow-up home visits from public health nurses are less common than in the developed world leading to gaps in mothers’ knowledge of breastfeeding. These mothers may benefit more after any educational intervention. Breastfeeding is also socially accepted as the norm in many cultures in developing countries, which would make mothers more eager to breastfeed after counseling. Mothers in developed countries may have increases in breastfeeding rates of lower magnitude due to wider availability of formula, work constraints and social perceptions.

Both individual and group counseling markedly increased the rates of exclusive breastfeeding, with combined individual and group counseling having the greatest effect from 1-5 months of age with a 101% increase. Combined individual and group counseling also led to a greater decrease in no breastfeeding rates, of 34% till 1 month and 32% for 1-5 months, than individual or group counseling alone. Receiving the combination of one-on-one educational sessions with group sessions may be the ideal combination for women as a motivating strategy to continue breastfeeding.

Observing the success of educational strategies for promoting breastfeeding in developing countries, we should consider introducing these strategies on a large scale, utilizing both facility-based care and resources at the community level. These interventions should involve well-timed individual counseling along with group sessions for helping mothers achieve the goal of EBF till 6 months and continued BF till two years of life.

## Conclusion

Breastfeeding education and/or support increased EBF rates and decreased no breastfeeding rates at birth, <1 month and 1-5 months. Combined individual and group counseling appeared to be superior to individual or group counseling alone. Interventions in developing countries had a greater impact than those in developed countries. These interventions have the potential to obtain optimum breast feeding practices and should be scaled up.

## Competing interests

We do not have any financial or non-financial competing interests for this review. The publications costs for this paper are from a grant from the Bill & Melinda Gates Foundation to the US Fund for UNICEF (grant 43386 to "Promote evidence-based decision making in designing maternal, neonatal, and child health interventions in low- and middle-income countries”).

## Authors' contributions

Dr ZAB was responsible for designing the review and coordinating the review. SH, JKD, AI and RAS were responsible for: data collection, screening the search results, screening retrieved papers against inclusion criteria, appraising quality of papers, abstracting data from papers, entering data into RevMan, analysis and interpretation of data and writing the review. ZAB, RAS and JKD critically reviewed and modified the manuscript.

## Supplementary Material

Additional File 1 Detailed quality of evidence tables for the subgroups. Click here for file

Additional File 2 Plots for meta analyses for day 1 and 0-1 month. Click here for file

## Declarations

The publication costs for this supplement were funded by a grant from the Bill & Melinda Gates Foundation to the US Fund for UNICEF (grant 43386 to "Promote evidence-based decision making in designing maternal, neonatal, and child health interventions in low- and middle-income countries”). The Supplement Editor is the principal investigator and lead in the development of the Lives Saved Tool (LiST), supported by grant 43386. He declares that he has no competing interests.

This article has been published as part of *BMC Public Health* Volume 13 Supplement 3, 2013: The Lives Saved Tool in 2013: new capabilities and applications. The full contents of the supplement are available online at http://www.biomedcentral.com/bmcpublichealth/supplements/13/S3.
