# Supplementary material for: Breastfeeding promotion interventions and breastfeeding practices: a systematic review
Source: BMC Public Health. 2013 Sep 17;13(Suppl 3):S20. doi: 10.1186/1471-2458-13-S3-S20 (PMC3847366; doi:10.1186/1471-2458-13-S3-S20)
Supplement: Additional File 1 — Detailed quality of evidence tables for the subgroups. [file 1471-2458-13-S3-S20-S1.pdf]

Table 1: Summary of findings for effect of breastfeeding promotion interventions on exclusive breastfeeding rates.

Quality Assessment						Summary of Findings			
				Directness		No of events			
No of studies	Design	Limitations	Consistency	Generalizability to population of interest	Generalizability to intervention of interest	Intervention	Control	Relative Risk (95% CI)	Comments
Rate of exclusive breastfeeding at day 1: low outcome-specific quality									
15	6 RCTs (14, 40, 49, 70, 71, 74), 9 QE (21, 32, 45, 48, 56, 59, 64, 85, 88)	Studies used different follow up periods and recall criteria. Mothers in the intervention group may have over-reported feeding practices.	6 of 15 studies suggest benefit. Significant heterogeneity	10 out of 15 studies were conducted in developed countries	Pooled results for different types of interventions.	4093	6316	1.43 [1.09, 1.87]	Random effects meta-analysis due to heterogeneity. The majority of studies used individual counseling as the intervention; most were facility and community-based.
11	4 RCTs (14, 49, 70, 74), 7 QE (32, 45, 48, 56, 59, 64, 69)	None	5 of 11 studies suggest benefit	4 of 11 studies were conducted in developing countries	Effect of individual counseling	3540	5772	1.60 [1.04, 2.48]	
2	1 RCT(40), 1 QE (21)	Standard care for comparison group included optional breastfeeding classes, peer support groups and home visit.	None of the studies suggests benefit	2 studies conducting in developed country.	Effect of group counseling	503	503	1.00 [0.94, 1.07]	Insignificant heterogeneity in this subgroup.
2	2 RCTs (71, 74)	None	1 of 2 studies suggests benefit	Both studies were conducted in developed countries	Effect of individual and group counseling	50	41	1.37 [0.93, 2.00]	
2	1 RCT, 1 QE (14, 59)	None	1 of 2 studies suggests benefit	1 study conducted in developed country	Effect of community-based intervention	285	103	4.84 [0.30, 78.76]	
5	1 RCT, 4 QE (21, 32, 45, 56, 71)	None	3 of 5 studies suggest benefit	Only 1 study conducted in developed country; rest in developing countries	Effect of facility-based intervention	461	199	1.67 [1.17, 2.38]	
7	4 RCTs, 3 QE (40, 48, 49, 64, 69, 70, 74)	None	2 of 7 studies suggest benefit	All studies conducted in developed countries	Effect of facility- and community-based intervention	3347	6014	1.05 [0.81, 1.36]	
5	1RCT, 4 QE (14, 21, 32, 45, 56)	None	3 of 5 studies suggest benefit	Developing countries	Effect of intervention in developing countries	618	192	2.57 [1.39, 4.77]	The majority of studies used individual counseling and were facility-based.
9	5 RCTs, 4 QE (40, 48, 49, 59, 64, 69-71, 74)	None	3 of 9 studies suggest benefit	Developed countries	Effect of intervention in developed countries	3475	6124	1.11 [0.87, 1.41]	The majority of studies were facility- and community-based.
**rate of exclusive breastfeeding at 0-1 month*: Low outcome-specific quality***									
30	22 RCTs, 8 QE (13, 17-19, 21, 22, 33, 38, 39, 41, 42, 45, 52, 53, 55-57, 60, 61, 65, 66, 68, 71-74, 76, 81, 84, 85)	Studies used different follow up periods. Recall criteria variable across studies (past 24 hr, past week or previous month). Mothers in the intervention group may	15 of 30 studies suggest benefit. Significant heterogeneity	16 of 30 studies were conducted in developed countries	Pooled results for different types of interventions	1512	1276	1.30 [1.19, 1.42]	Random effects meta-analysis due to heterogeneity. The majority of studies used individual counseling

		have over-reported feeding practices.							as the intervention; most were facility and community-based.
24	19 RCTs, 5 QE (13, 17, 18, 33, 38, 39, 41, 42, 45, 52, 53, 55, 56, 60, 61, 65, 66, 68, 72-74, 76, 81, 85)		12 of 24 studies suggest benefit	13 of 24 studies were conducted in developed countries	Effect of individual counseling	1167	973	1.31 [1.17, 1.46]	
3	1 RCT, 2 QE (56, 57, 84)		1 of 3 studies suggests benefit	2 of 3 studies were conducted in developed countries	Effect of group counseling	102	90	1.56 [0.83, 2.93]	All studies had facility-based interventions.
5	4 RCTs, 1 QE (19, 22, 57, 71, 74)		2 of 5 studies suggest benefit	3 of 5 studies were conducted in developed countries	Effect of individual and group counseling	243	213	1.27 [1.09, 1.49]	Most studies had facility and community-based interventions.
2	2 RCTs (13, 18)		1 of 2 studies suggests benefit	1 of 2 studies conducted in developed country	Effect of community-based intervention	165	86	1.78 [0.73, 4.37]	
13	9 RCTs, 4 QE (21, 45, 52, 53, 55-57, 61, 68, 71, 76, 84, 85)		7 of 13 studies suggest benefit	6 of 13 studies were conducted in developed countries	Effect of facility-based intervention	647	550	1.26 [1.11, 1.43]	
15	11 RCTs, 4 QE (17, 19, 22, 33, 38, 39, 41, 42, 60, 65, 66, 72-74, 81)		7 of 15 studies suggest benefit	9 of 15 studies were conducted in developed countries	Effect of facility- and community-based intervention	700	640	1.31 [1.14, 1.50]	The majority of studies used individual counseling.
14	9 RCTs, 6 QE (13, 19, 21, 22, 33, 39, 42, 45, 52, 55, 56, 76, 81, 85)		7 of 14 studies suggest benefit	Developing countries	Effect of intervention in developing countries	706	607	1.35 [1.15, 1.58]	The majority of studies used individual counseling.
16	14 RCTs, 2 QE (17, 18, 38, 41, 53, 57, 60, 61, 65, 66, 68, 71-74, 84)	One study (57)included predominant BF in the category of EBF	8 of 16 studies suggest benefit	Developed countries	Effect of intervention in developed countries	806	669	1.26 [1.13, 1.41]	Most studies used individual counseling and were facility- and community-based.
**rate of exclusive breastfeeding at 1-6 months*: low outcome-specific quality***									
53	34 RCTs, 19 QE (11-14, 19, 20, 22, 33-38, 40-52, 54, 57-59, 61-65, 67, 69, 71-83, 85-87)	Variable follow up periods used in studies. Recall criteria variable across studies (past 24 hr, past week or previous month). Mothers in the intervention group may have over-reported feeding practices.	21 of 53 studies suggest benefit. Significant heterogeneity	29 of 53 studies were conducted in developed countries	Pooled results for different types of interventions	5481	4897	1.90 [1.54, 2.34]	Random effects meta-analysis due to heterogeneity. The majority of studies used individual counseling as the intervention; most were facility and community-based
39	28 RCTs, 11 QE (12-14, 33, 35, 37, 38, 41-50, 52, 54, 58, 59, 61-65, 67, 69, 72-74, 76, 80-83, 85-87)		13 of 39 studies suggest benefit	24 of 39 studies were conducted in developed countries	Effect of individual counseling	3440	3320	1.90 [1.34, 2.68]	
6	3 RCTs, 3 QE (20, 40, 51, 57, 75, 78)		3 of 6 studies suggest benefit	4 of 6 studies were conducted in developed countries	Effect of group counseling	298	151	1.80 [1.18, 2.74]	

10	6 RCTs, 4 QE (11, 19, 22, 34, 36, 57, 71, 74, 77, 79)	One study (79) allowed supplemental feeding of less than 2 ounces to be grouped with EBF.	6 of 10 studies suggest benefit	5 of 10 studies were conducted in developing countries	Effect of individual and group counseling	1743	1426	2.01 [1.43, 2.82]	
14	9 RCTs, 5 QE (11, 13, 14, 35-37, 43, 44, 50, 51, 59, 62, 78, 87)		10 of 14 studies suggest benefit	8 of 14 studies were conducted in developing countries	Effect of community-based intervention	1737	656	2.59 [1.80, 3.73]	Most studies used individual counseling.
19	13 RCTs, 6 QE (12, 20, 45, 47, 52, 54, 57, 58, 61, 67, 71, 72, 75-77, 79, 80, 82, 85)		4 of 19 studies suggest benefit	11 of 19 studies were conducted in developed countries	Effect of facility-based intervention	1418	420	1.87 [1.26, 2.78]	Most studies used individual counseling.
20	13 RCTs, 7 QE (19, 22, 33, 34, 38, 40-42, 46, 48, 49, 63-65, 69, 73, 74, 81, 83, 86)		7 of 20 studies suggest benefit	13 of 20 studies were conducted in developed countries	Effect of facility- and community-based intervention	2326	3821	1.47 [1.08, 1.99]	Most studies used individual counseling.
24	14 RCTs, 10 QE (11-14, 19, 22, 33-37, 42, 45-47, 52, 75, 76, 78-81, 85, 87)		16 of 24 studies suggest benefit	Developing countries	Effect of intervention in developing countries	3797	1909	2.88 [2.11, 3.93]	Most studies used individual counseling.
29	22 RCTs, 7 QE (20, 38, 40, 41, 43, 44, 48-51, 54, 57-59, 61-65, 67, 69, 71-74, 77, 82, 83, 86)	2 studies (57, 82) included predominant BF in the category of EBF	5 of 29 studies suggest benefit	Developed countries	Effect of intervention in developed countries	1684	2988	1.31 [1.00, 1.70]	Most studies used individual counseling and were facility- and community-based.

Table 2: Summary of findings for effect of breastfeeding promotion interventions on predominant and partial breastfeeding rates.

	Quality Assessment					Summary of Findings			
				Directness		No of events			
No of studies	Design	Limitations	Consistency	Generalizability to population of interest	Generalizability to intervention of interest	Intervention	Control	Relative Risk (95% CI)	Comments
**rate of predominant breastfeeding at 0-1 month*: Moderate outcome-specific quality***									
6	5 RCTs, 1 QE (18, 52, 61, 72, 76, 85)	Variable follow up periods used in studies. Recall criteria variable across studies (past 24 hr, past week or previous month). mothers in the intervention group may have over-reported feeding practices	None of the studies suggest benefit. Insignificant heterogeneity	3 studies were conducted in developing countries	Pooled results for different types of interventions	33	59	0.66 [0.43, 1.01]	Fixed effects meta-analysis; insignificant heterogeneity. Most studies were facility-based and all used individual counseling.
6	5 RCTs, 1 QE (18, 52, 61, 72, 76, 85)		None of the studies suggest benefit	3 studies were conducted in developing countries	Effect of individual counseling	33	59	0.66 [0.43, 1.01]	Fixed effects meta-analysis; insignificant heterogeneity. Most studies had facility-based interventions.
1	1 RCT (18)	Low event rat		Developed country	Effect of community-based intervention	4	6	0.63 [0.18, 2.17]	All studies used individual counseling.
5	4 RCTs, 1 QE (52, 61, 72, 76, 85)		None of the studies suggest benefit.	3 of 5 studies were conducted in developing countries	Effect of facility-based intervention	29	53	0.66 [0.42, 1.04]	Most studies used individual counseling.
3	2 RCTs, 1 QE (52, 76, 85)		None of the studies suggest benefit	Developing countries	Effect of intervention in developing countries	26	39	0.67 [0.42, 1.06]	All studies were facility-based and used individual counseling.
3	3 RCTs (18, 61, 72)		None of the studies suggest benefit	Developed countries	Effect of intervention in developed countries	7	20	0.63 [0.23, 1.72]	All studies used individual counseling.
**rate of predominant breastfeeding at 1- 6 months*: low outcome specific-quality***									
13	10 RCTs, 3 QE (11, 12, 18, 20, 36, 42, 46, 52, 58, 61, 72, 76, 85)	Variable follow up periods used in studies. Recall criteria variable across studies (past 24 hr, past week or previous month). mothers in the intervention group may have over-reported feeding practices	2 studies suggest benefit. Significant heterogeneity	8 of 13 studies were conducted in developed countries	Pooled results for different types of interventions	1433	707	1.08 [0.55, 2.13]	Random effects meta-analysis due to significant heterogeneity. Most studies are facility-based and used individual counseling.
10	8 RCTs, 2 QE (12, 18, 42, 46, 52, 58, 61, 72, 76, 85)		2 studies suggest benefit	6 of 10 studies were conducted in developing countries	Effect of individual counseling	1309	473	1.34 [0.62, 2.90]	Most studies had facility-based interventions.
1	1 RCT (20)	No events. Small sample size	-	Study was conducted in developed country	Effect of group counseling	0	0	-	The study used a facility-based intervention.

2	1 RCT, 1 QE (11, 36)	In one study (11), follow-up assessment 1 year after intervention leading to recall bias	None of the studies suggest benefit	Both studies were conducted in developing countries	Effect of individual and group counseling	124	234	0.48 [0.12, 1.94]	All studies had community-based interventions.
3	2 RCTs, 1 QE (11, 18, 36)	In one study (11), follow-up assessment 1 year after intervention leading to recall bias	None of the studies suggest benefit	2 of 3 studies were conducted in developing countries	Effect of community-based intervention	125	243	0.36 [0.10, 1.26]	Most studies used individual and group counseling.
8	7 RCTs, 1 QE (12, 20, 52, 58, 61, 72, 76, 85)		2 studies suggest effect of benefit	4 of 8 studies were conducted in developing countries	Effect of facility-based intervention	1160	333	2.26 [0.77, 6.67]	Most studies used individual counseling.
2	1 RCT, 1 QE (42, 46)		None of the studies suggest benefit	1 of 2 studies was conducted in a developing country	Effect of facility- and community-based intervention	148	131	0.72 [0.23, 2.26]	All studies used individual counseling.
8	5 RCTs, 3 QE (11, 12, 36, 42, 46, 52, 76, 85)	In one study (Dearden 2002), follow-up assessment 1 year after intervention leading to recall bias	2 studies suggest benefit	Developing countries	Effect of intervention in developing countries	1246	516	1.23 [0.49, 3.08]	Most studies used individual counseling and 4 of 8 were facility-based.
5	5 RCTs (18, 20, 58, 61, 72)		None of the studies suggest benefit	Developed countries	Effect of intervention in developed countries	187	191	0.80 [0.26, 2.48]	Most studies used individual counseling.
**rate of partial breastfeeding at day 1*: low outcome-specific quality***									
6	2 RCT, 4 QE (45, 49, 59, 64, 71, 89)	Variable follow up periods used in studies. Recall criteria variable across studies (past 24 hr, past week or previous month). Mothers in the intervention group may have over-reported feeding practices.	1 of 6 studies suggests benefit. Significant heterogeneity	1 of 6 studies was conducted in a developing country	Pooled results for different types of interventions	101	99	1.21 [0.79, 1.87]	Random effects meta-analysis due to significant heterogeneity. Most studies used individual counseling and most were facility-based.
5	1 RCT, 4 QE (45, 49, 59, 64, 89)		1 of 5 studies suggests benefit.	1 of 5 studies was conducted in a developing country	Effect of individual counseling	98	92	1.32 [0.84, 2.08]	
1	1 RCT (71)	Small sample size (<50)	-	Study conducted in developed country	Effect of individual and group counseling	3	7	0.48 [0.14, 1.66]	The study was a facility-based intervention.
1	1 QE (59)		-	Study conducted in developed country	Effect of community-based intervention	16	4	4.25 [1.43, 12.61]	The study used individual counseling.
3	1 RCT, 2 QE (45, 71, 89)		No significant effect observed in any study.	1 of 3 studies was conducted in a developing country	Effect of facility-based intervention	41	49	0.84 [0.63, 1.13]	Insignificant heterogeneity in this subgroup Most studies used individual counseling.
2	1 RCT, 1 QE (49, 64)		No significant effect observed in any study.	Both studies were conducted in developed countries	Effect of facility- and community-based intervention	44	46	1.29 [0.88, 1.90]	Insignificant heterogeneity in this subgroup
1	1 QE (45)		-	Developing countries	Effect of intervention in developing countries	31	37	0.84 [0.61, 1.15]	The study had individual counseling and was facility-based.
5	2 RCTs, 3 QE (49, 59, 64, 71, 89)		1 of 5 studies suggests benefit.	Developed countries	Effect of intervention in developed countries	70	62	1.40 [0.83, 2.35]	Most studies used individual counseling.

**rate of partial breastfeeding at 0-1 month*: moderate outcome-specific quality***									
11	8 RCTs, 3 QE (18, 19, 45, 52, 61, 66, 71, 72, 76, 85, 88)	Variable follow up periods used in studies. Recall criteria variable across studies (past 24 hr, past week or previous month). mothers in the intervention group may have over-reported feeding practices.	None of the studies suggest benefit. Insignificant heterogeneity	5 of 11 studies were conducted in developing countries	Pooled results for different types of interventions	112	151	0.88 [0.72, 1.08]	Fixed effects meta-analysis; insignificant heterogeneity Most studies used individual counseling and most were facility-based.
9	7 RCTs, 2 QE (18, 45, 52, 61, 66, 72, 76, 85, 88)		No significant effect observed in any study.	4 of 9 studies were conducted in developing countries	Effect of individual counseling	108	130	0.90 [0.73, 1.11]	Most studies were facility-based.
2	1 RCT, 1 QE (19, 71)		None of the studies suggest benefit	1 of 2 studies were conducted in developing countries	Effect of individual and group counseling	4	21	0.48 [0.16, 1.38]	
1	1 RCT (18)		-	Study was conducted in a developed country	Effect of community-based interventions	17	18	0.89 [0.48, 1.64]	The study used individual counseling.
8	6 RCTs, 2 QE (45, 52, 61, 71, 72, 76, 85, 88)		No significant effect observed in any study.	4 of 8 studies were conducted in developing countries	Effect of facility-based interventions	91	110	0.92 [0.74, 1.16]	Most studies used individual counseling.
2	1 RCT, 1 QE (19, 66)		None of the studies suggest benefit	1 of 2 studies was conducted in a developing country	Effect of facility- and community-based interventions	4	23	0.34 [0.13, 0.93]	One study used individual counseling.
5	2 RCTs, 3 QE (19, 45, 52, 76, 85)		None of the studies suggest benefit	Developing countries	Effect of intervention in developing countries	57	79	0.94 [0.72, 1.24]	Both studies used individual counseling and were facility-based.
6	6 RCTs (18, 61, 64, 66, 71, 88)		No significant effect observed in any study.	Developed countries	Effect of intervention in developed countries	55	72	0.80 [0.59, 1.10]	Most studies used individual counseling.
**rate of partial breastfeeding at 1-6 months*: moderate outcome-specific quality***									
20	11 RCTs, 9 QE (18-20, 36, 42, 45, 47, 49, 51, 52, 59, 61, 62, 71, 72, 76, 80, 85, 86, 89)	Variable follow up periods used in studies. Recall criteria variable across studies (past 24 hr, past week or previous month). mothers in the intervention group may have over-reported feeding practices.	None of the studies suggest benefit. Significant heterogeneity	9 of 20 studies were conducted in developing countries	Pooled results for different types of interventions	524	578	0.87 [0.75, 1.02]	Random effects meta-analysis due to significant heterogeneity. Most studies used individual counseling and most were facility-based.
15	8 RCTs, 7 QE (18, 42, 45, 47, 49, 52, 59, 61, 62, 72, 76, 80, 85, 86, 89)		None of the studies suggest benefit. Insignificant heterogeneity	7 of 15 studies were conducted in developing countries	Effect of individual counseling	402	421	0.93 [0.80, 1.09]	Insignificant heterogeneity in this subgroup Most studies were facility-based.
2	1 RCT, 1 QE (20, 51)		None of the studies suggest benefit. Insignificant heterogeneity	1 of the 2 studies was conducted in a developing country	Effect of group counseling	19	23	0.59 [0.30, 1.15]	Insignificant heterogeneity in this subgroup
3	2 RCTs, 1 QE (19, 36, 71)		None of the studies suggest benefit	2 of 3 studies were conducted in developing	Effect of individual and group counseling	103	134	0.82 [0.51, 1.31]	

				countries					
5	3 RCTs, 2 QE (18, 36, 51, 59, 62)		None of the studies suggest benefit	4 of 5 studies were conducted in developed countries	Effect of community-based interventions	130	162	0.71 [0.50, 1.00]	Insignificant heterogeneity in this subgroup
11	7 RCTs, 4 QE (20, 45, 47, 52, 61, 71, 72, 76, 80, 85, 89)		None of the studies suggest benefit	6 of 11 studies were conducted in developing countries	Effect of facility-based interventions	308	318	0.93 [0.75, 1.16]	Most studies used individual counseling.
4	1 RCT, 3 QE (19, 42, 49, 86)		No significant effect observed in any study.	2 of 4 studies were conducted in developing countries	Effect of facility- and community-based interventions	86	98	0.94 [0.75, 1.17]	Insignificant heterogeneity in this subgroup. Most studies used individual counseling.
9	4 RCTs, 5 QE (19, 36, 42, 45, 47, 52, 76, 80, 85)		None of the studies suggest benefit	Developing countries	Effect of intervention in developing countries	337	380	0.83 [0.65, 1.06]	Both studies had individual counseling and were facility-based.
11	7 RCTs, 4 QE (18, 20, 49, 51, 59, 61, 62, 71, 72, 86, 89)		None of the studies suggest benefit Insignificant heterogeneity	Developed countries	Effect of intervention in developed countries	187	198	0.94 [0.79, 1.12]	Insignificant heterogeneity in this subgroup. Most studies used individual counseling.

Table 3: Summary of findings for effect of breastfeeding promotion interventions on 'no breastfeeding' rates.

	Quality Assessment						Summary of Findings		
				Directness		No of events			
No of studies	Design	Limitations	Consistency	Generalizability to population of interest	Generalizability to intervention of interest	Intervention	Control	Relative Risk (95% CI)	Comments
rate of no breastfeeding at day 1*: low outcome-specific quality*									
38	21 RCTs, 17 QE (11, 13, 14, 17, 33, 36, 37, 44, 49, 59, 63, 64, 68-71, 74, 78, 79, 89-91, 96, 100, 103, 107, 108, 110, 111, 113, 117, 120-122, 124-127)	Variable follow up periods used in studies. Recall criteria variable across studies (past 24 hr, past week or previous month).	Most studies suggest benefit. Significant heterogeneity	10 of 38 studies were conducted in developing countries	Pooled results for different types of interventions	48026	39843	0.68 [0.54, 0.87]	Random effects meta-analysis due to significant heterogeneity<br><br>Most studies used individual counseling and most were facility and community-based.<br><br>Effect of benefit refers to decrease in numbers not breastfeeding.
27	15 RCTs, 12 QE (13, 14, 17, 33, 37, 44, 49, 59, 63, 64, 68-70, 74, 89-91, 103, 107, 108, 110, 111, 113, 120-122, 125)		Half of studies suggest benefit	5 of 27 studies were conducted in developing countries	Effect of individual counseling	46766	37039	0.73 [0.55, 0.96]	Most studies were facility and community-based.
3	1 RCT, 2 QE (78, 117, 124)		Consistent effect of benefit in all studies	1 of 3 studies was conducted in developing countries	Effect of group counseling	87	172	0.57 [0.41, 0.80]	Most studies were facility-based.
9	5 RCTs, 4 QE (11, 36, 71, 74, 79, 96, 100, 126, 127)		Most studies suggest benefit	4 of 9 studies were conducted in developing countries	Effect of individual and group counseling	1173	2632	0.64 [0.39, 1.04]	
9	4 RCTs, 5 QE (11, 13, 14, 36, 37, 59, 78, 96, 100)		Half of studies suggest benefit	7 of 9 studies were conducted in developing countries	Effect of community-based interventions	1748	3536	0.74 [0.51, 1.07]	
11	6 RCTs, 5 QE (68, 71, 79, 89, 90, 110, 111, 117, 120, 122, 124)		Almost all studies suggest benefit	2 of 11 studies were conducted in developing countries	Effect of facility-based interventions	327	483	0.48 [0.34, 0.69]	
18	10 RCTs, 8 QE (17, 33, 44, 49, 63, 64, 69, 70, 74, 91, 103, 107, 108, 113, 121, 125-127)		Most studies suggest benefit	1 of 18 studies were conducted in developing countries	Effect of facility- and community-based interventions	45951	35824	0.81 [0.58, 1.12]	
10	6 RCTs, 4 QE (11, 13, 14, 33, 36, 37, 78, 79, 100, 122)		8 of 10 studies suggest benefit	Developing countries	Effect of intervention in developing countries	1487	2559	0.58 [0.44, 0.78]	
28	15 RCTs, 13 QE (17, 44, 49, 59, 63, 64, 68-71, 74, 89-91, 96, 103, 107, 108, 110, 111, 113, 117, 120, 121, 124-127)		10 of 28 studies suggest benefit	Developed countries	Effect of intervention in developed countries	46539	37284	0.73 [0.57, 0.95]	

**rate of no breastfeeding at 0-1 month*: low outcome-specific quality***									
33	21 RCTs, 12 QE (10, 18, 19, 38, 45, 52, 53, 61, 63, 66, 68, 71-74, 76, 88, 90, 91, 93, 95, 98, 99, 101-103, 106, 113, 117, 118, 120, 124, 128)	Variable follow up periods used in studies. Recall criteria variable across studies (past 24 hr, past week or previous month).	10 of 33 studies suggest benefit. Significant heterogeneity	4 of 33 studies were conducted in developing countries	Pooled results for different types of interventions	770	1018	0.70 [0.62, 0.80]	Random effects meta-analysis due to significant heterogeneity. Most studies used individual counseling.
26	19 RCTs, 7 QE (10, 18, 38, 45, 52, 53, 61, 63, 66, 68, 72-74, 76, 88, 90, 91, 93, 98, 101, 103, 106, 113, 118, 120, 128)		7 of 26 studies suggest benefit.	3 of 26 studies were conducted in developing countries	Effect of individual counseling	603	829	0.71 [0.61, 0.84]	
3	1 RCT, 2 QE (99, 117, 124)		2 of 3 studies suggest benefit.	All in developed countries	Effect of group counseling	75	91	0.71 [0.51, 0.99]	Most studies were facility-based.
5	2 RCTs, 3 QE (19, 71, 74, 95, 102)		1 of 5 studies suggests benefit	1 out of 5 studies were conducted in developing countries	Effect of individual and group counseling	92	98	0.66 [0.51, 0.87]	
3	2 RCTs, 1 QE (18, 101, 106)		1 of 3 studies suggest benefit	All in developed countries	Effect of community-based interventions	137	151	0.90 [0.51, 1.57]	
16	11 RCTs, 5 QE (45, 52, 53, 61, 68, 71, 76, 88, 90, 98, 99, 102, 117, 120, 124, 128)		6 of 16 studies suggest benefit.	3 out of 16 studies were conducted in developing countries	Effect of facility-based interventions	334	490	0.68 [0.56, 0.83]	
14	8 RCTs, 6 QE (10, 19, 38, 63, 66, 72-74, 91, 93, 95, 103, 113, 118)		3 of 14 studies suggest benefit.	1 out of 14 studies was conducted in a developing country	Effect of facility- and community-based interventions	299	377	0.67 [0.54, 0.83]	
4	2 RCTs, 2 QE (19, 45, 52, 76)		1 study suggests benefit	Developing countries	Effect of intervention in developing countries	16	34	0.51 [0.29, 0.90]	
29	19 RCTs, 10 QE (10, 18, 38, 53, 61, 63, 66, 68, 71-74, 88, 90, 91, 93, 95, 98, 99, 101-103, 106, 113, 117, 118, 120, 124, 128)		9 of 29 studies suggest benefit.	Developed countries	Effect of intervention in developed countries	754	984	0.71 [0.62, 0.81]	
**rate of no breastfeeding at 1-6 months*: low outcome-specific quality***									
73	41 RCTs, 32 QE (10, 12, 13, 17-20, 35, 36, 38, 40-42, 44, 45, 47, 49, 51, 52, 54, 58, 59, 61-64, 67-69, 71-74, 76, 78-83, 86, 90-93, 95-99, 101, 103-106, 108-110, 112-121, 123, 124, 126, 129, 130)	Variable follow up periods used in studies. Recall criteria variable across studies (past 24 hr, past week or previous month).	25 of 73 studies suggest benefit. Significant heterogeneity	16 of 73 studies were conducted in developing countries	Pooled results for different types of interventions	15473	17578	0.82 [0.77, 0.89]	Random effects meta-analysis due to significant heterogeneity Most studies used individual counseling.

53	37 RCTs, 16 QE (10, 12, 13, 17, 18, 35, 38, 41, 42, 44, 45, 47, 49, 52, 54, 58, 59, 61-64, 67-69, 72-74, 76, 79, 81-83, 86, 90, 91, 93, 97, 98, 101, 103, 105, 106, 108, 110, 112, 113, 118-121, 123, 129, 130)		15 of 53 studies suggest benefit	11 of 53 studies were conducted in developing countries	Effect of individual counseling	14375	14943	0.86 [0.79, 0.94]	
10	3 RCTs, 7 QE (20, 40, 51, 78, 92, 99, 109, 115, 117, 124)		3 of 10 studies suggest benefit	2 of 10 studies were conducted in developing countries	Effect of group counseling	449	503	0.76 [0.63, 0.91]	Most studies were facility-based.
11	3 RCTs, 8 QE (19, 36, 71, 74, 80, 95, 96, 104, 114, 116, 126)		9 of 11 studies suggest benefit	3 of 11 studies were conducted in developing countries	Effect of individual and group counseling	649	2132	0.68 [0.51, 0.91]	Most studies were facility and community-based.
12	7 RCTs, 5 QE (13, 18, 35, 36, 51, 59, 62, 78, 96, 101, 105, 106)		3 of 12 studies suggest benefit	4 of 12 studies were conducted in developing countries	Effect of community-based interventions	1263	2782	0.89 [0.75, 1.06]	Most studies used individual counseling.
31	21 RCTs, 10 QE (12, 20, 45, 47, 52, 54, 58, 61, 67, 68, 71, 72, 76, 79, 80, 82, 90, 92, 98, 99, 109, 110, 115-117, 119, 120, 123, 124, 129, 130)		9 of 31 studies suggest benefit	8 of 31 studies were conducted in developing countries	Effect of facility-based interventions	5548	6515	0.82 [0.75, 0.89]	Most studies used individual counseling.
31	13 RCTs, 18 QE (10, 17, 19, 38, 40-42, 44, 49, 63, 64, 69, 72-74, 81, 83, 86, 91, 93, 95, 97, 103, 104, 108, 112-114, 118, 121, 126)		13 of 31studies suggest benefit	4 of 31 studies were conducted in developing countries	Effect of facility- and community-based interventions	8804	8538	0.83 [0.75, 0.93]	Most studies used individual counseling.
16	8 RCTs, 8 QE (12, 13, 19, 35, 36, 42, 45, 47, 52, 76, 78-81, 92, 97)		10 of 16 studies suggest benefit	Developing countries	Effect of intervention in developing countries	4710	5708	0.56 [0.45, 0.69]	Most studies used individual counseling.
57	33 RCTs, 24 QE (10, 17, 18, 20, 38, 40, 41, 44, 49, 51, 54, 58, 59, 61-64, 67-69, 71-74, 82, 83, 86, 90, 91, 93, 95, 96, 98, 99, 101, 103-106, 108-110, 112-121, 123, 124, 126, 129, 130)		15 of 57 studies suggest benefit	Developed countries	Effect of intervention in developed countries	10905	12127	0.88 [0.82, 0.95]	Most studies used individual counseling.
