# Supplementary material for: Breastfeeding promotion interventions and breastfeeding practices: a systematic review
Source: BMC Public Health. 2013 Sep 17;13(Suppl 3):S20. doi: 10.1186/1471-2458-13-S3-S20 (PMC3847366; doi:10.1186/1471-2458-13-S3-S20)

Figure 1a. Effect of breastfeeding education on the rate of exclusive breastfeeding at day 1: individual vs group counseling.

Study or Subgroup	log[Risk Ratio]	SE	Weight	Risk Ratio IV, Random, 95% CI
**1.2.4 individual and group counseling**				
Sciacca 1995	0.4721	0.1818	6.2%	1.60 [1.12, 2.29]
Su 2007b	0.077	0.2501	5.7%	1.08 [0.66, 1.76]
**Subtotal (95% CI)**			**11.9%**	**1.37 [0.93, 2.00]**
Heterogeneity: Tau² = 0.03; Chi² = 1.63, df = 1 (P = 0.20); I² = 39%				
Test for overall effect: Z = 1.61 (P = 0.11)				
**1.2.5 individual counseling**				
Abolyan 2006	0.9993	0.078	6.8%	2.72 [2.33, 3.17]
Haider 2000	3.0172	0.3325	4.9%	20.43 [10.65, 39.21]
Huang 2007	0.2318	0.2113	6.0%	1.26 [0.83, 1.91]
Kistin 1994	0.6571	0.2021	6.1%	1.93 [1.30, 2.87]
Kools 2005	-0.1037	0.0568	6.9%	0.90 [0.81, 1.01]
Lin CH 2008	0.4228	0.1571	6.4%	1.53 [1.12, 2.08]
McInnes 2000	0.1712	0.1259	6.6%	1.19 [0.93, 1.52]
Nommsen-Rivers 2009	-0.1456	0.1912	6.1%	0.86 [0.59, 1.26]
Ryan 2006	-0.552	0.0191	7.0%	0.58 [0.55, 0.60]
Sandy 2009	0.4836	0.2356	5.8%	1.62 [1.02, 2.57]
Su 2007a	0.3941	0.2304	5.8%	1.48 [0.94, 2.33]
**Subtotal (95% CI)**			**68.4%**	**1.60 [1.04, 2.48]**
Heterogeneity: Tau² = 0.51; Chi² = 628.01, df = 10 (P < 0.00001); I² = 98%				
Test for overall effect: Z = 2.12 (P = 0.03)				
**1.2.6 group counseling**				
Forster 2004a	-0.0037	0.0429	6.9%	1.00 [0.92, 1.08]
Forster 2004b	-0.006	0.0429	6.9%	0.99 [0.91, 1.08]
Lin SS 2008	0.3137	0.2182	5.9%	1.37 [0.89, 2.10]
**Subtotal (95% CI)**			**19.8%**	**1.00 [0.94, 1.07]**
Heterogeneity: Tau² = 0.00; Chi² = 2.09, df = 2 (P = 0.35); I² = 4%				
Test for overall effect: Z = 0.05 (P = 0.96)				
**Total (95% CI)**			**100.0%**	**1.43 [1.09, 1.87]**
Heterogeneity: Tau² = 0.27; Chi² = 768.36, df = 15 (P < 0.00001); I² = 98%				
Test for overall effect: Z = 2.61 (P = 0.009)				
Test for subgroup differences: Chi² = 6.73, df = 2 (P = 0.03), I² = 70.3%				

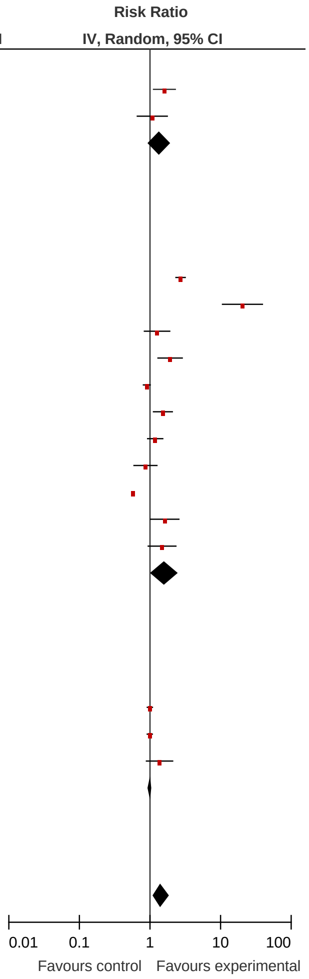

Figure 1b. Effect of breastfeeding education on the rate of exclusive breastfeeding at day 1: community- vs facility-based interventions.

Study or Subgroup	log[Risk Ratio]	SE	Weight	Risk Ratio IV, Random, 95% CI
**1.4.4 community-based intervention**				
Haider 2000	3.0172	0.3325	4.9%	20.43 [10.65, 39.21]
McInnes 2000	0.1712	0.1259	6.6%	1.19 [0.93, 1.52]
**Subtotal (95% CI)**			**11.5%**	**4.84 [0.30, 78.76]**
Heterogeneity: Tau² = 3.99; Chi² = 64.08, df = 1 (P < 0.00001); I² = 98%				
Test for overall effect: Z = 1.11 (P = 0.27)				
**1.4.5 facility-based intervention**				
Abolyan 2006	0.9993	0.078	6.8%	2.72 [2.33, 3.17]
Huang 2007	0.2318	0.2113	6.0%	1.26 [0.83, 1.91]
Lin CH 2008	0.4228	0.1571	6.4%	1.53 [1.12, 2.08]
Lin SS 2008	0.3137	0.2182	5.9%	1.37 [0.89, 2.10]
Sciacca 1995	0.4721	0.1818	6.2%	1.60 [1.12, 2.29]
**Subtotal (95% CI)**			**31.3%**	**1.67 [1.17, 2.38]**
Heterogeneity: Tau² = 0.14; Chi² = 26.89, df = 4 (P < 0.0001); I² = 85%				
Test for overall effect: Z = 2.82 (P = 0.005)				
**1.4.6 facility- and community-based intervention**				
Forster 2004a	-0.0037	0.0429	6.9%	1.00 [0.92, 1.08]
Forster 2004b	-0.006	0.0429	6.9%	0.99 [0.91, 1.08]
Kistin 1994	0.6571	0.2021	6.1%	1.93 [1.30, 2.87]
Kools 2005	-0.1037	0.0568	6.9%	0.90 [0.81, 1.01]
Nommsen-Rivers 2009	-0.1456	0.1912	6.1%	0.86 [0.59, 1.26]
Ryan 2006	-0.552	0.0191	7.0%	0.58 [0.55, 0.60]
Sandy 2009	0.4836	0.2356	5.8%	1.62 [1.02, 2.57]
Su 2007a	0.3941	0.2304	5.8%	1.48 [0.94, 2.33]
Su 2007b	0.077	0.2501	5.7%	1.08 [0.66, 1.76]
**Subtotal (95% CI)**			**57.2%**	**1.05 [0.81, 1.36]**
Heterogeneity: Tau² = 0.13; Chi² = 311.64, df = 8 (P < 0.00001); I² = 97%				
Test for overall effect: Z = 0.40 (P = 0.69)				
**Total (95% CI)**			**100.0%**	**1.43 [1.09, 1.87]**
Heterogeneity: Tau² = 0.27; Chi² = 768.36, df = 15 (P < 0.00001); I² = 98%				
Test for overall effect: Z = 2.61 (P = 0.009)				
Test for subgroup differences: Chi² = 5.11, df = 2 (P = 0.08), I² = 60.9%				

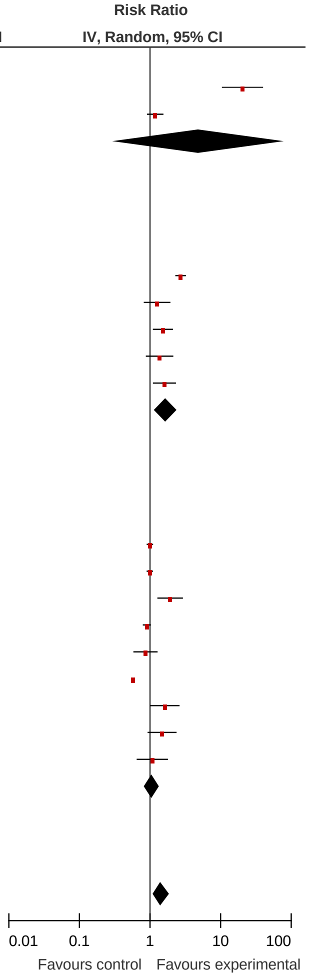

Figure 1c. Effect of breastfeeding education on the rate of exclusive breastfeeding at day 1: developing vs developed countries.

Study or Subgroup	log[Risk Ratio]	SE	Weight	Risk Ratio IV, Random, 95% CI
**1.6.1 developing countries**				
Abolyan 2006	0.9993	0.078	6.8%	2.72 [2.33, 3.17]
Haider 2000	3.0172	0.3325	4.9%	20.43 [10.65, 39.21]
Huang 2007	0.2318	0.2113	6.0%	1.26 [0.83, 1.91]
Lin CH 2008	0.4228	0.1571	6.4%	1.53 [1.12, 2.08]
Lin SS 2008	0.3137	0.2182	5.9%	1.37 [0.89, 2.10]
**Subtotal (95% CI)**			**30.1%**	**2.57 [1.39, 4.77]**
Heterogeneity: Tau² = 0.45; Chi² = 68.13, df = 4 (P < 0.00001); I² = 94%				
Test for overall effect: Z = 3.00 (P = 0.003)				
**1.6.2 developed countries**				
Forster 2004a	-0.0037	0.0429	6.9%	1.00 [0.92, 1.08]
Forster 2004b	-0.006	0.0429	6.9%	0.99 [0.91, 1.08]
Kistin 1994	0.6571	0.2021	6.1%	1.93 [1.30, 2.87]
Kools 2005	-0.1037	0.0568	6.9%	0.90 [0.81, 1.01]
McInnes 2000	0.1712	0.1259	6.6%	1.19 [0.93, 1.52]
Nommsen-Rivers 2009	-0.1456	0.1912	6.1%	0.86 [0.59, 1.26]
Ryan 2006	-0.552	0.0191	7.0%	0.58 [0.55, 0.60]
Sandy 2009	0.4836	0.2356	5.8%	1.62 [1.02, 2.57]
Sciacca 1995	0.4721	0.1818	6.2%	1.60 [1.12, 2.29]
Su 2007a	0.3941	0.2304	5.8%	1.48 [0.94, 2.33]
Su 2007b	0.077	0.2501	5.7%	1.08 [0.66, 1.76]
**Subtotal (95% CI)**			**69.9%**	**1.11 [0.87, 1.41]**
Heterogeneity: Tau² = 0.14; Chi² = 349.43, df = 10 (P < 0.00001); I² = 97%				
Test for overall effect: Z = 0.83 (P = 0.40)				
**Total (95% CI)**			**100.0%**	**1.43 [1.09, 1.87]**
Heterogeneity: Tau² = 0.27; Chi² = 768.36, df = 15 (P < 0.00001); I² = 98%				
Test for overall effect: Z = 2.61 (P = 0.009)				
Test for subgroup differences: Chi² = 6.24, df = 1 (P = 0.01), I² = 84.0%				

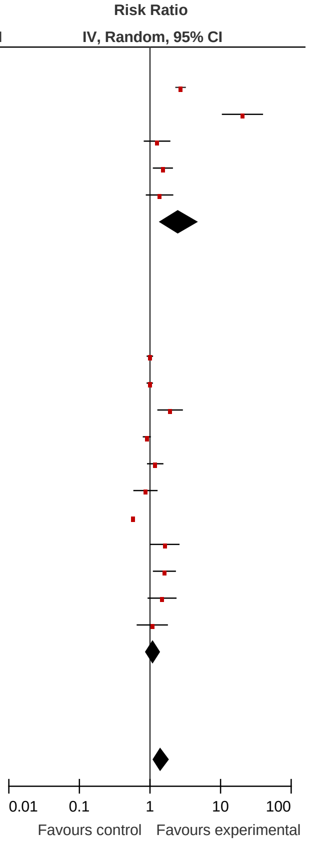

Figure 2a. Effect of breastfeeding education on the rate of exclusive breastfeeding at 0-1 month: individual vs group counseling.

Study or Subgroup	log[Risk Ratio]	SE	Weight	Risk Ratio IV, Random, 95% CI
**1.8.4 individual and group counseling**				
Aidam 2005a	0.1996	0.1593	3.2%	1.22 [0.89, 1.67]
Aidam 2005b	0.2719	0.1513	3.3%	1.31 [0.98, 1.77]
Alvarado 1996	0.2748	0.0707	4.6%	1.32 [1.15, 1.51]
Mattar 2007a	0.0541	0.0588	4.8%	1.06 [0.94, 1.18]
Sciacca 1995	0.8511	0.2733	1.8%	2.34 [1.37, 4.00]
Su 2007b	0.2736	0.2205	2.4%	1.31 [0.85, 2.03]
**Subtotal (95% CI)**			**20.2%**	**1.27 [1.09, 1.49]**
Heterogeneity: Tau² = 0.02; Chi² = 12.97, df = 5 (P = 0.02); I² = 61%				
Test for overall effect: Z = 2.99 (P = 0.003)				
**1.8.5 individual counseling**				
Aksu 2011	0.5108	0.2582	2.0%	1.67 [1.00, 2.76]
Anderson 2005	1.3573	0.4786	0.8%	3.89 [1.52, 9.93]
Bonuck 2006	0.0583	0.2337	2.2%	1.06 [0.67, 1.68]
de Oliveira 2006	-0.1394	0.1299	3.7%	0.87 [0.67, 1.12]
Dennis 2002	0.1657	0.0859	4.4%	1.18 [1.00, 1.40]
Frank 1987a	0.075	0.14	3.5%	1.08 [0.82, 1.42]
Frank 1987b	0.1655	0.139	3.5%	1.18 [0.90, 1.55]
Froozani 1999	0.7085	0.1434	3.5%	2.03 [1.53, 2.69]
Huang 2007	0.4595	0.3204	1.5%	1.58 [0.84, 2.97]
Kupratakul 2010	0.387	0.1759	3.0%	1.47 [1.04, 2.08]
Labarere 2005	0.1543	0.0716	4.6%	1.17 [1.01, 1.34]
Langer 1996	0.5478	0.2403	2.1%	1.73 [1.08, 2.77]
Lin CH 2008	0.7806	0.2159	2.4%	2.18 [1.43, 3.33]
McKeever 2002	0.2575	0.1088	4.0%	1.29 [1.05, 1.60]
McQueen 2011	0.1002	0.143	3.5%	1.11 [0.84, 1.46]
Morrow 1999	1.0873	0.3164	1.5%	2.97 [1.60, 5.51]
Petrova 2009	0.2441	0.3721	1.2%	1.28 [0.62, 2.65]
Porteous 2000	0.8546	0.2795	1.8%	2.35 [1.36, 4.06]
Rasmussen 2011	-0.3542	0.3251	1.4%	0.70 [0.37, 1.33]
Simonetti 2012	0.589	0.1693	3.0%	1.80 [1.29, 2.51]
Sjolin 1979	0.1335	0.0886	4.4%	1.14 [0.96, 1.36]
Su 2007a	0.5996	0.2034	2.6%	1.82 [1.22, 2.71]
Tahir 2012	0.1213	0.0542	4.9%	1.13 [1.02, 1.26]
Vitolo 2005	-0.359	0.1308	3.7%	0.70 [0.54, 0.90]
Wong 2007	-0.0267	0.1826	2.9%	0.97 [0.68, 1.39]
**Subtotal (95% CI)**			**71.9%**	**1.31 [1.17, 1.46]**
Heterogeneity: Tau² = 0.05; Chi² = 87.92, df = 24 (P < 0.00001); I² = 73%				
Test for overall effect: Z = 4.62 (P < 0.00001)				
**1.8.6 group counseling**				
Lin SS 2008	0.47	0.2547	2.0%	1.60 [0.97, 2.64]
Mattar 2007b	-0.0344	0.0691	4.7%	0.97 [0.84, 1.11]
Wiles 1984	1.0986	0.3496	1.3%	3.00 [1.51, 5.95]
**Subtotal (95% CI)**			**8.0%**	**1.56 [0.83, 2.93]**
Heterogeneity: Tau² = 0.26; Chi² = 13.18, df = 2 (P = 0.001); I² = 85%				
Test for overall effect: Z = 1.37 (P = 0.17)				
**Total (95% CI)**			**100.0%**	**1.30 [1.19, 1.42]**
Heterogeneity: Tau² = 0.04; Chi² = 118.41, df = 33 (P < 0.00001); I² = 72%				
Test for overall effect: Z = 5.71 (P < 0.00001)				
Test for subgroup differences: Chi² = 0.38, df = 2 (P = 0.83), I² = 0%				

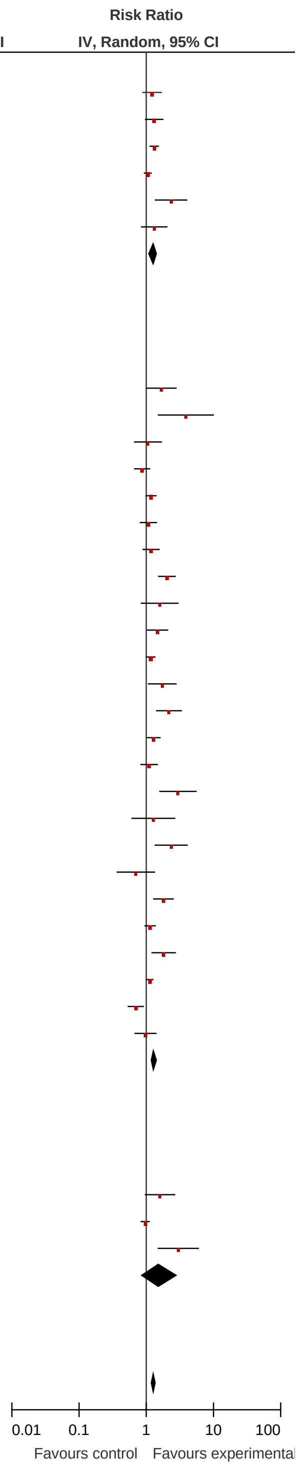

Figure 2b. Effect of breastfeeding education on the rate of exclusive breastfeeding at 0-1 month: community- vs facility-based interventions.

Study or Subgroup	log[Risk Ratio]	SE	Weight	Risk Ratio IV, Random, 95% CI
**1.10.4 community-based intervention**				
Dennis 2002	0.1657	0.0859	4.4%	1.18 [1.00, 1.40]
Morrow 1999	1.0873	0.3164	1.5%	2.97 [1.60, 5.51]
**Subtotal (95% CI)**			**5.9%**	**1.78 [0.73, 4.37]**
Heterogeneity: Tau² = 0.37; Chi² = 7.90, df = 1 (P = 0.005); I² = 87%				
Test for overall effect: Z = 1.26 (P = 0.21)				
**1.10.5 facility-based intervention**				
Huang 2007	0.4595	0.3204	1.5%	1.58 [0.84, 2.97]
Kupratakul 2010	0.387	0.1759	3.0%	1.47 [1.04, 2.08]
Labarere 2005	0.1543	0.0716	4.6%	1.17 [1.01, 1.34]
Langer 1996	0.5478	0.2403	2.1%	1.73 [1.08, 2.77]
Lin CH 2008	0.7806	0.2159	2.4%	2.18 [1.43, 3.33]
Lin SS 2008	0.47	0.2547	2.0%	1.60 [0.97, 2.64]
Mattar 2007a	0.0541	0.0588	4.8%	1.06 [0.94, 1.18]
Mattar 2007b	-0.0344	0.0691	4.7%	0.97 [0.84, 1.11]
McQueen 2011	0.1002	0.143	3.5%	1.11 [0.84, 1.46]
Rasmussen 2011	-0.3542	0.3251	1.4%	0.70 [0.37, 1.33]
Sciacca 1995	0.8511	0.2733	1.8%	2.34 [1.37, 4.00]
Tahir 2012	0.1213	0.0542	4.9%	1.13 [1.02, 1.26]
Wiles 1984	1.0986	0.3496	1.3%	3.00 [1.51, 5.95]
Wong 2007	-0.0267	0.1826	2.9%	0.97 [0.68, 1.39]
**Subtotal (95% CI)**			**40.8%**	**1.26 [1.11, 1.43]**
Heterogeneity: Tau² = 0.03; Chi² = 42.10, df = 13 (P < 0.0001); I² = 69%				
Test for overall effect: Z = 3.55 (P = 0.0004)				
**1.10.6 facility- and community-based intervention**				
Aidam 2005a	0.1996	0.1593	3.2%	1.22 [0.89, 1.67]
Aidam 2005b	0.2719	0.1513	3.3%	1.31 [0.98, 1.77]
Aksu 2011	0.5108	0.2582	2.0%	1.67 [1.00, 2.76]
Alvarado 1996	0.2748	0.0707	4.6%	1.32 [1.15, 1.51]
Anderson 2005	1.3573	0.4786	0.8%	3.89 [1.52, 9.93]
Bonuck 2006	0.0583	0.2337	2.2%	1.06 [0.67, 1.68]
de Oliveira 2006	-0.1394	0.1299	3.7%	0.87 [0.67, 1.12]
Frank 1987a	0.075	0.14	3.5%	1.08 [0.82, 1.42]
Frank 1987b	0.1655	0.139	3.5%	1.18 [0.90, 1.55]
Froozani 1999	0.7085	0.1434	3.5%	2.03 [1.53, 2.69]
McKeever 2002	0.2575	0.1088	4.0%	1.29 [1.05, 1.60]
Petrova 2009	0.2441	0.3721	1.2%	1.28 [0.62, 2.65]
Porteous 2000	0.8546	0.2795	1.8%	2.35 [1.36, 4.06]
Simonetti 2012	0.589	0.1693	3.0%	1.80 [1.29, 2.51]
Sjolin 1979	0.1335	0.0886	4.4%	1.14 [0.96, 1.36]
Su 2007a	0.5996	0.2034	2.6%	1.82 [1.22, 2.71]
Su 2007b	0.2736	0.2205	2.4%	1.31 [0.85, 2.03]
Vitolo 2005	-0.359	0.1308	3.7%	0.70 [0.54, 0.90]
**Subtotal (95% CI)**			**53.3%**	**1.31 [1.14, 1.50]**
Heterogeneity: Tau² = 0.06; Chi² = 62.73, df = 17 (P < 0.00001); I² = 73%				
Test for overall effect: Z = 3.82 (P = 0.0001)				
**Total (95% CI)**			**100.0%**	**1.30 [1.19, 1.42]**
Heterogeneity: Tau² = 0.04; Chi² = 118.41, df = 33 (P < 0.00001); I² = 72%				
Test for overall effect: Z = 5.71 (P < 0.00001)				
Test for subgroup differences: Chi² = 0.69, df = 2 (P = 0.71), I² = 0%				

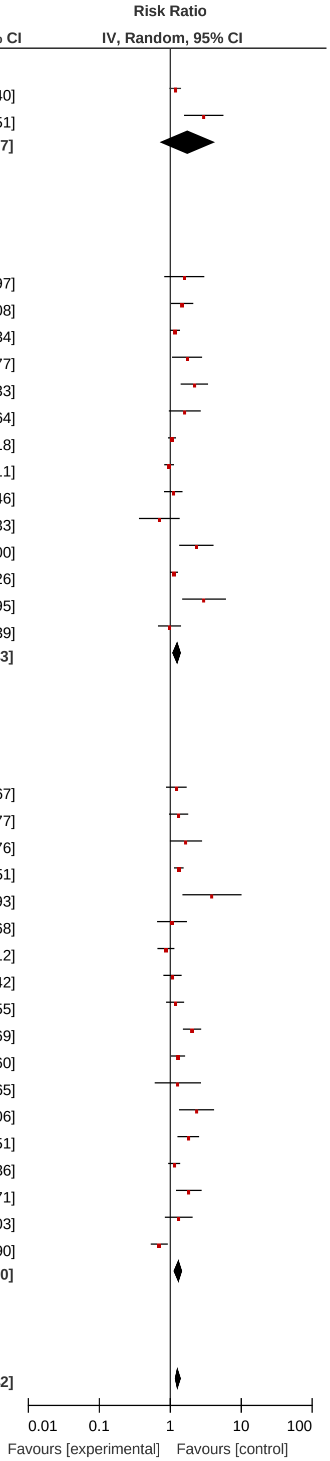

Figure 2c. Effect of breastfeeding education on the rate of exclusive breastfeeding at 0-1 month: developing vs developed countries.

Study or Subgroup	log[]	SE	Weight	IV, Random, 95% CI
**1.12.1 developing countries**				
Aidam 2005a	0.1996	0.1593	3.2%	1.22 [0.89, 1.67]
Aidam 2005b	0.2719	0.1513	3.3%	1.31 [0.98, 1.77]
Aksu 2011	0.5108	0.2582	2.0%	1.67 [1.00, 2.76]
Alvarado 1996	0.2748	0.0707	4.6%	1.32 [1.15, 1.51]
de Oliveira 2006	-0.1394	0.1299	3.7%	0.87 [0.67, 1.12]
Froozani 1999	0.7085	0.1434	3.5%	2.03 [1.53, 2.69]
Huang 2007	0.4595	0.3204	1.5%	1.58 [0.84, 2.97]
Kupratakul 2010	0.387	0.1759	3.0%	1.47 [1.04, 2.08]
Langer 1996	0.5478	0.2403	2.1%	1.73 [1.08, 2.77]
Lin CH 2008	0.7806	0.2159	2.4%	2.18 [1.43, 3.33]
Lin SS 2008	0.47	0.2547	2.0%	1.60 [0.97, 2.64]
Morrow 1999	1.0873	0.3164	1.5%	2.97 [1.60, 5.51]
Tahir 2012	0.1213	0.0542	4.9%	1.13 [1.02, 1.26]
Vitolo 2005	-0.359	0.1308	3.7%	0.70 [0.54, 0.90]
Wong 2007	-0.0267	0.1826	2.9%	0.97 [0.68, 1.39]
**Subtotal (95% CI)**			**44.1%**	**1.35 [1.15, 1.58]**
Heterogeneity: Tau² = 0.07; Chi² = 64.21, df = 14 (P < 0.00001); I² = 78%				
Test for overall effect: Z = 3.65 (P = 0.0003)				
**1.12.2 developed countries**				
Anderson 2005	1.3573	0.4786	0.8%	3.89 [1.52, 9.93]
Bonuck 2006	0.0583	0.2337	2.2%	1.06 [0.67, 1.68]
Dennis 2002	0.1657	0.0859	4.4%	1.18 [1.00, 1.40]
Frank 1987a	0.075	0.14	3.5%	1.08 [0.82, 1.42]
Frank 1987b	0.1655	0.139	3.5%	1.18 [0.90, 1.55]
Labarere 2005	0.1543	0.0716	4.6%	1.17 [1.01, 1.34]
Mattar 2007a	0.0541	0.0588	4.8%	1.06 [0.94, 1.18]
Mattar 2007b	-0.0344	0.0691	4.7%	0.97 [0.84, 1.11]
McKeever 2002	0.2575	0.1088	4.0%	1.29 [1.05, 1.60]
McQueen 2011	0.1002	0.143	3.5%	1.11 [0.84, 1.46]
Petrova 2009	0.2441	0.3721	1.2%	1.28 [0.62, 2.65]
Porteous 2000	0.8546	0.2795	1.8%	2.35 [1.36, 4.06]
Rasmussen 2011	-0.3542	0.3251	1.4%	0.70 [0.37, 1.33]
Sciacca 1995	0.8511	0.2733	1.8%	2.34 [1.37, 4.00]
Simonetti 2012	0.589	0.1693	3.0%	1.80 [1.29, 2.51]
Sjolin 1979	0.1335	0.0886	4.4%	1.14 [0.96, 1.36]
Su 2007a	0.5996	0.2034	2.6%	1.82 [1.22, 2.71]
Su 2007b	0.2736	0.2205	2.4%	1.31 [0.85, 2.03]
Wiles 1984	1.0986	0.3496	1.3%	3.00 [1.51, 5.95]
**Subtotal (95% CI)**			**55.9%**	**1.26 [1.13, 1.41]**
Heterogeneity: Tau² = 0.03; Chi² = 52.42, df = 18 (P < 0.0001); I² = 66%				
Test for overall effect: Z = 4.25 (P < 0.0001)				
**Total (95% CI)**			**100.0%**	**1.30 [1.19, 1.42]**
Heterogeneity: Tau² = 0.04; Chi² = 118.41, df = 33 (P < 0.00001); I² = 72%				
Test for overall effect: Z = 5.71 (P < 0.00001)				
Test for subgroup differences: Chi² = 0.41, df = 1 (P = 0.52), I² = 0%				

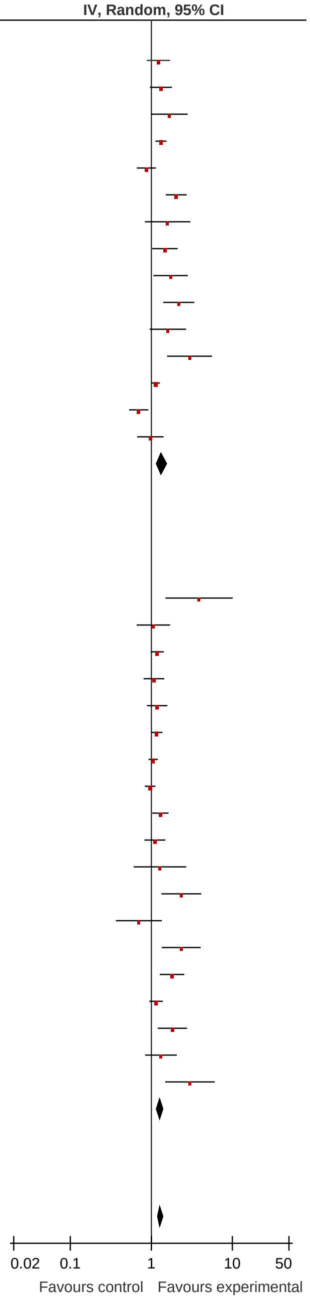

Figure 3a. Effect of breastfeeding education on the rate of exclusive breastfeeding at 1-6 months: individual vs group counseling.

Study or Subgroup	log[Risk Ratio]	SE	Weight	Risk Ratio IV, Random, 95% CI
**1.14.4 individual and group counseling**				
Aidam 2005a	0.6587	0.1595	1.9%	1.93 [1.41, 2.64]
Aidam 2005b	0.4686	0.1751	1.9%	1.60 [1.13, 2.25]
Alvarado 1996	4.0318	1.4167	0.4%	56.36 [3.51, 905.49]
Arifeen 2009	0.145	0.0233	2.0%	1.16 [1.10, 1.21]
Bhandari 2003	2.3688	0.2512	1.8%	10.68 [6.53, 17.48]
Dearden 2002	0.0981	0.1473	1.9%	1.10 [0.83, 1.47]
Mattar 2007a	0.7472	0.3881	1.6%	2.11 [0.99, 4.52]
Sciacca 1995	0.8977	0.4669	1.5%	2.45 [0.98, 6.13]
Su 2007b	0.7699	0.3439	1.7%	2.16 [1.10, 4.24]
Taveras 2011	-0.0715	0.2554	1.8%	0.93 [0.56, 1.54]
Valdes 1993	0.748	0.0899	2.0%	2.11 [1.77, 2.52]
**Subtotal (95% CI)**			**18.6%**	**2.01 [1.43, 2.82]**
Heterogeneity: Tau² = 0.25; Chi² = 143.30, df = 10 (P < 0.00001); I² = 93%				
Test for overall effect: Z = 4.00 (P < 0.0001)				
**1.14.5 individual counseling**				
Aksu 2011	0.619	0.3913	1.6%	1.86 [0.86, 4.00]
Bashour 2008a	0.3469	0.1604	1.9%	1.41 [1.03, 1.94]
Bashour 2008b	0.4003	0.1563	1.9%	1.49 [1.10, 2.03]
Bhutta 2008	0.4329	0.0928	2.0%	1.54 [1.29, 1.85]
Bonuck 2006	-0.4384	0.4915	1.4%	0.65 [0.25, 1.69]
Frank 1987a	-0.7051	0.8521	0.9%	0.49 [0.09, 2.62]
Frank 1987b	0.3237	0.5634	1.3%	1.38 [0.46, 4.17]
Froozani 1999	2.1128	0.4979	1.4%	8.27 [3.12, 21.95]
Gijsbers 2006	0.5821	0.2933	1.8%	1.79 [1.01, 3.18]
Graffy 2004	0.1804	0.124	2.0%	1.20 [0.94, 1.53]
Haider 2000	2.4646	0.2383	1.8%	11.76 [7.37, 18.76]
Huang 2007	0.2877	0.3354	1.7%	1.33 [0.69, 2.57]
Jakobsen 1999	0.0891	0.3427	1.7%	1.09 [0.56, 2.14]
Khresheh 2011	0.3483	0.3126	1.7%	1.42 [0.77, 2.61]
Kistin 1994	1.4183	0.5933	1.2%	4.13 [1.29, 13.21]
Kools 2005	-0.1583	0.1182	2.0%	0.85 [0.68, 1.08]
Kramer 2001	2.579	0.147	1.9%	13.18 [9.88, 17.59]
Kronborg 2007	0.4326	0.1986	1.9%	1.54 [1.04, 2.27]
Kupratakul 2010	2.7832	1.4379	0.4%	16.17 [0.97, 270.81]
Labarere 2003	-0.032	0.3568	1.6%	0.97 [0.48, 1.95]
McDonald 2008	0.0491	0.1524	1.9%	1.05 [0.78, 1.42]
McInnes 2000	0.4465	0.2503	1.8%	1.56 [0.96, 2.55]
McQueen 2011	0.1171	0.1802	1.9%	1.12 [0.79, 1.60]
Morrell 2000	0.0547	0.2406	1.8%	1.06 [0.66, 1.69]
Morrow 1999	1.2773	0.4836	1.4%	3.59 [1.39, 9.25]
Muirhead 2006	1.6182	1.5435	0.4%	5.04 [0.24, 103.90]
Nommsen-Rivers 2009	0.2868	0.2839	1.8%	1.33 [0.76, 2.32]
Petrova 2009	0.2772	0.6292	1.2%	1.32 [0.38, 4.53]
Pugh 2002	0.6444	0.6343	1.2%	1.90 [0.55, 6.60]
Ryan 2006	-0.862	0.0379	2.0%	0.42 [0.39, 0.45]
Simonetti 2012	0.7634	0.4233	1.5%	2.15 [0.94, 4.92]
Sjolin 1979	0.1671	0.3771	1.6%	1.18 [0.56, 2.47]
Su 2007a	0.7503	0.3464	1.7%	2.12 [1.07, 4.18]
Tahir 2012	0.0409	0.2861	1.8%	1.04 [0.59, 1.83]
Tylleskar 2011a	2.0188	0.1577	1.9%	7.53 [5.53, 10.26]
Tylleskar 2011b	1.529	0.1552	1.9%	4.61 [3.40, 6.25]
Tylleskar 2011c	2.2045	1.0469	0.7%	9.07 [1.16, 70.55]
Valdes 2000	2.1808	0.3744	1.6%	8.85 [4.25, 18.44]
Vitolo 2005	0.8511	0.2729	1.8%	2.34 [1.37, 4.00]
Wallace 2006	-0.0351	0.5235	1.4%	0.97 [0.35, 2.69]
Wen 2011	0.711	0.4928	1.4%	2.04 [0.78, 5.35]
Wong 2007	0.6931	1.2166	0.6%	2.00 [0.18, 21.71]
Wrenn 1997	0.0367	0.1889	1.9%	1.04 [0.72, 1.50]
**Subtotal (95% CI)**			**67.4%**	**1.90 [1.34, 2.68]**
Heterogeneity: Tau² = 1.14; Chi² = 1338.44, df = 42 (P < 0.00001); I² = 97%				
Test for overall effect: Z = 3.64 (P = 0.0003)				

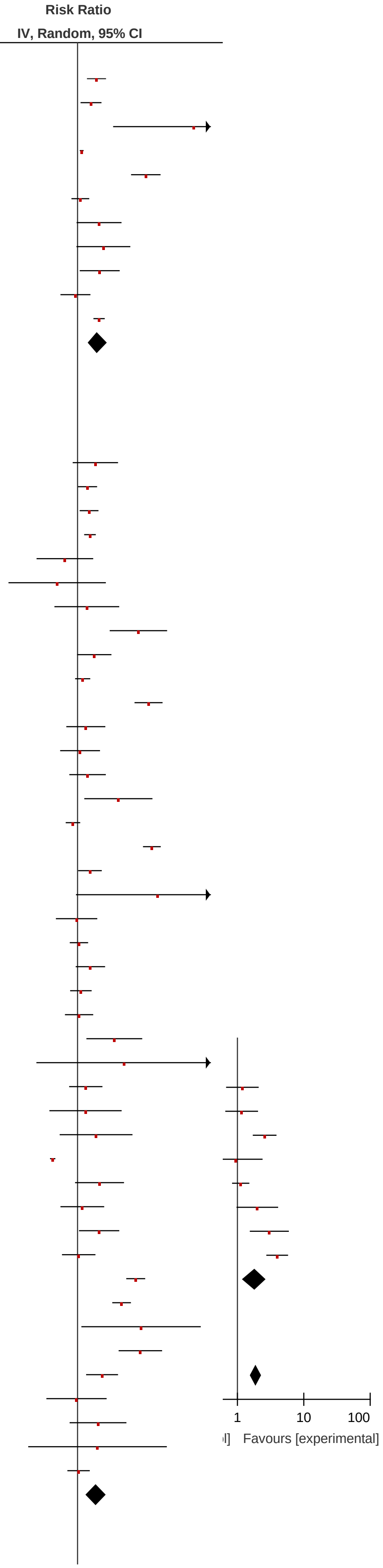

Figure 3b. Effect of breastfeeding education on the rate of exclusive breastfeeding at 1-6 months: community- vs facility-based interventions.

Study or Subgroup	log[Risk Ratio]	SE	Weight	Risk Ratio IV, Random, 95% CI
**1.16.4 community-based intervention**				
Bashour 2008a	0.3469	0.1604	1.9%	1.41 [1.03, 1.94]
Bashour 2008b	0.4003	0.1563	1.9%	1.49 [1.10, 2.03]
Bhandari 2003	2.3688	0.2512	1.8%	10.68 [6.53, 17.48]
Bhutta 2008	0.4329	0.0928	2.0%	1.54 [1.29, 1.85]
Dearden 2002	0.0981	0.1473	1.9%	1.10 [0.83, 1.47]
Gijsbers 2006	0.5821	0.2933	1.8%	1.79 [1.01, 3.18]
Graffy 2004	0.1804	0.124	2.0%	1.20 [0.94, 1.53]
Haider 2000	2.4646	0.2383	1.8%	11.76 [7.37, 18.76]
Kronborg 2007	0.4326	0.1986	1.9%	1.54 [1.04, 2.27]
Kruske 2007	0.9455	0.1998	1.9%	2.57 [1.74, 3.81]
McInnes 2000	0.4465	0.2503	1.8%	1.56 [0.96, 2.55]
Morrell 2000	0.0547	0.2406	1.8%	1.06 [0.66, 1.69]
Morrow 1999	1.2773	0.4836	1.4%	3.59 [1.39, 9.25]
Turan 2003	1.3816	0.1828	1.9%	3.98 [2.78, 5.70]
Tylleskar 2011a	2.0188	0.1577	1.9%	7.53 [5.53, 10.26]
Tylleskar 2011b	1.529	0.1552	1.9%	4.61 [3.40, 6.25]
Tylleskar 2011c	2.2045	1.0469	0.7%	9.07 [1.16, 70.55]
**Subtotal (95% CI)**			**30.4%**	**2.59 [1.80, 3.73]**
Heterogeneity: Tau² = 0.52; Chi² = 275.57, df = 16 (P < 0.00001); I² = 94%				
Test for overall effect: Z = 5.10 (P < 0.00001)				
**1.16.5 facility-based intervention**				
Huang 2007	0.2877	0.3354	1.7%	1.33 [0.69, 2.57]
Khresheh 2011	0.3483	0.3126	1.7%	1.42 [0.77, 2.61]
Kramer 2001	2.579	0.147	1.9%	13.18 [9.88, 17.59]
Kupratakul 2010	2.7832	1.4379	0.4%	16.17 [0.97, 270.81]
Labarere 2003	-0.032	0.3568	1.6%	0.97 [0.48, 1.95]
Mattar 2007a	0.7472	0.3881	1.6%	2.11 [0.99, 4.52]
Mattar 2007b	-0.0525	0.463	1.5%	0.95 [0.38, 2.35]
McDonald 2008	0.0491	0.1524	1.9%	1.05 [0.78, 1.42]
McQueen 2011	0.1171	0.1802	1.9%	1.12 [0.79, 1.60]
Noel-Weiss 2006	0.1156	0.1428	1.9%	1.12 [0.85, 1.49]
Pugh 2002	0.6444	0.6343	1.2%	1.90 [0.55, 6.60]
Sciacca 1995	0.8977	0.4669	1.5%	2.45 [0.98, 6.13]
Susin 2008a	0.6924	0.3583	1.6%	2.00 [0.99, 4.03]
Susin 2008b	1.1085	0.3347	1.7%	3.03 [1.57, 5.84]
Tahir 2012	0.0409	0.2861	1.8%	1.04 [0.59, 1.83]
Taveras 2011	-0.0715	0.2554	1.8%	0.93 [0.56, 1.54]
Valdes 1993	0.748	0.0899	2.0%	2.11 [1.77, 2.52]
Valdes 2000	2.1808	0.3744	1.6%	8.85 [4.25, 18.44]
Wallace 2006	-0.0351	0.5235	1.4%	0.97 [0.35, 2.69]
Wong 2007	0.6931	1.2166	0.6%	2.00 [0.18, 21.71]
**Subtotal (95% CI)**			**31.3%**	**1.86 [1.23, 2.80]**
Heterogeneity: Tau² = 0.70; Chi² = 254.54, df = 19 (P < 0.00001); I² = 93%				
Test for overall effect: Z = 2.96 (P = 0.003)				

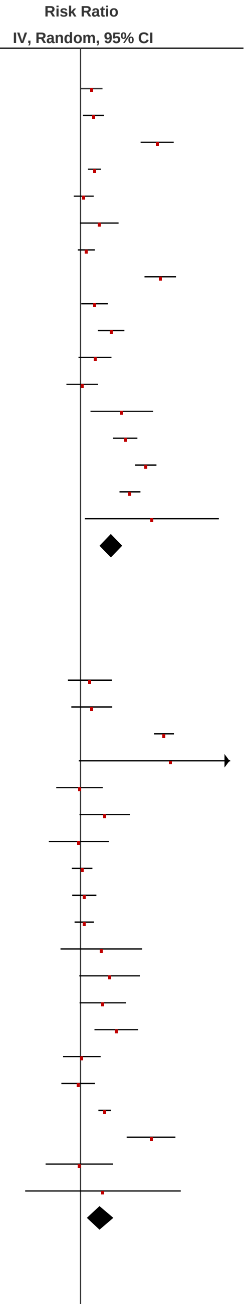

1.16.6 facility- and community-based intervention

Aidam 2005a	0.6587	0.1595	1.9%	1.93 [1.41, 2.64]
Aidam 2005b	0.4686	0.1751	1.9%	1.60 [1.13, 2.25]
Aksu 2011	0.619	0.3913	1.6%	1.86 [0.86, 4.00]
Alvarado 1996	4.0318	1.4167	0.4%	56.36 [3.51, 905.49]
Arifeen 2009	0.145	0.0233	2.0%	1.16 [1.10, 1.21]
Bonuck 2006	-0.4384	0.4915	1.4%	0.65 [0.25, 1.69]
Forster 2004a	0.1738	0.2779	1.8%	1.19 [0.69, 2.05]
Forster 2004b	0.1481	0.2805	1.8%	1.16 [0.67, 2.01]
Frank 1987a	-0.7051	0.8521	0.9%	0.49 [0.09, 2.62]
Frank 1987b	0.3237	0.5634	1.3%	1.38 [0.46, 4.17]
Froozani 1999	2.1128	0.4979	1.4%	8.27 [3.12, 21.95]
Jakobsen 1999	0.0891	0.3427	1.7%	1.09 [0.56, 2.14]
Kistin 1994	1.4183	0.5933	1.2%	4.13 [1.29, 13.21]
Kools 2005	-0.1583	0.1182	2.0%	0.85 [0.68, 1.08]
Muirhead 2006	1.6182	1.5435	0.4%	5.04 [0.24, 103.90]
Nommsen-Rivers 2009	0.2868	0.2839	1.8%	1.33 [0.76, 2.32]
Petrova 2009	0.2772	0.6292	1.2%	1.32 [0.38, 4.53]
Ryan 2006	-0.862	0.0379	2.0%	0.42 [0.39, 0.45]
Simonetti 2012	0.7634	0.4233	1.5%	2.15 [0.94, 4.92]
Sjolin 1979	0.1671	0.3771	1.6%	1.18 [0.56, 2.47]
Su 2007a	0.7503	0.3464	1.7%	2.12 [1.07, 4.18]
Su 2007b	0.7699	0.3439	1.7%	2.16 [1.10, 4.24]
Vitolo 2005	0.8511	0.2729	1.8%	2.34 [1.37, 4.00]
Wen 2011	0.711	0.4928	1.4%	2.04 [0.78, 5.35]
Wrenn 1997	0.0367	0.1889	1.9%	1.04 [0.72, 1.50]
**Subtotal (95% CI)**			**38.2%**	**1.49 [1.10, 2.00]**

Heterogeneity: Tau² = 0.41; Chi² = 625.35, df = 24 (P < 0.00001); I² = 96%

Test for overall effect: Z = 2.60 (P = 0.009)

**Total (95% CI)**	**100.0%**	**1.90 [1.54, 2.34]**

Heterogeneity: Tau² = 0.57; Chi² = 1629.52, df = 61 (P < 0.00001); I² = 96%

Test for overall effect: Z = 5.97 (P < 0.00001)

Test for subgroup differences: Chi² = 5.29, df = 2 (P = 0.07), I² = 62.2%

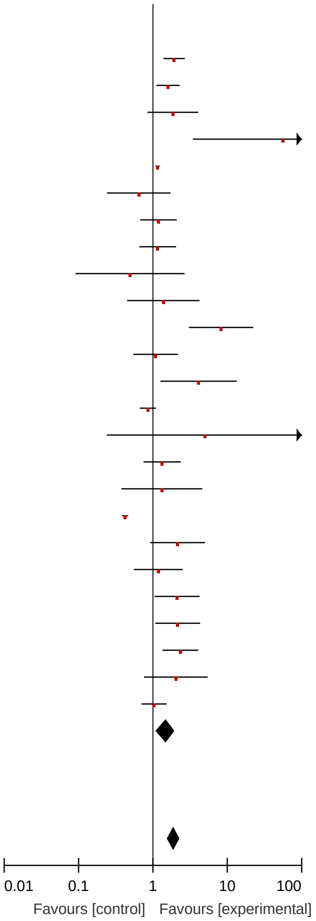

Figure 3c. Effect of breastfeeding education on the rate of exclusive breastfeeding at 1-6 months: developing vs developed countries.

Study or Subgroup	log[Risk Ratio]	SE	Weight	Risk Ratio IV, Random, 95% CI
**1.18.1 developing countries**				
Aidam 2005a	0.6587	0.1595	1.9%	1.93 [1.41, 2.64]
Aidam 2005b	0.4686	0.1751	1.9%	1.60 [1.13, 2.25]
Aksu 2011	0.619	0.3913	1.6%	1.86 [0.86, 4.00]
Alvarado 1996	4.0318	1.4167	0.4%	56.36 [3.51, 905.49]
Arifeen 2009	0.145	0.0233	2.0%	1.16 [1.10, 1.21]
Bashour 2008a	0.3469	0.1604	1.9%	1.41 [1.03, 1.94]
Bashour 2008b	0.4003	0.1563	1.9%	1.49 [1.10, 2.03]
Bhandari 2003	2.3688	0.2512	1.8%	10.68 [6.53, 17.48]
Bhutta 2008	0.4329	0.0928	2.0%	1.54 [1.29, 1.85]
Dearden 2002	0.0981	0.1473	1.9%	1.10 [0.83, 1.47]
Froozani 1999	2.1128	0.4979	1.4%	8.27 [3.12, 21.95]
Haider 2000	2.4646	0.2383	1.8%	11.76 [7.37, 18.76]
Huang 2007	0.2877	0.3354	1.7%	1.33 [0.69, 2.57]
Jakobsen 1999	0.0891	0.3427	1.7%	1.09 [0.56, 2.14]
Khresheh 2011	0.3483	0.3126	1.7%	1.42 [0.77, 2.61]
Kramer 2001	2.579	0.147	1.9%	13.18 [9.88, 17.59]
Kupratakul 2010	2.7832	1.4379	0.4%	16.17 [0.97, 270.81]
Morrow 1999	1.2773	0.4836	1.4%	3.59 [1.39, 9.25]
Susin 2008a	0.6924	0.3583	1.6%	2.00 [0.99, 4.03]
Susin 2008b	1.1085	0.3347	1.7%	3.03 [1.57, 5.84]
Tahir 2012	0.0409	0.2861	1.8%	1.04 [0.59, 1.83]
Turan 2003	1.3816	0.1828	1.9%	3.98 [2.78, 5.70]
Tylleskar 2011a	2.0188	0.1577	1.9%	7.53 [5.53, 10.26]
Tylleskar 2011b	1.529	0.1552	1.9%	4.61 [3.40, 6.25]
Tylleskar 2011c	2.2045	1.0469	0.7%	9.07 [1.16, 70.55]
Valdes 1993	0.748	0.0899	2.0%	2.11 [1.77, 2.52]
Valdes 2000	2.1808	0.3744	1.6%	8.85 [4.25, 18.44]
Vitolo 2005	0.8511	0.2729	1.8%	2.34 [1.37, 4.00]
Wong 2007	0.6931	1.2166	0.6%	2.00 [0.18, 21.71]
**Subtotal (95% CI)**			**47.1%**	**2.88 [2.11, 3.93]**

Heterogeneity: Tau² = 0.58; Chi² = 743.59, df = 28 (P < 0.00001); I² = 96%

Test for overall effect: Z = 6.71 (P < 0.00001)

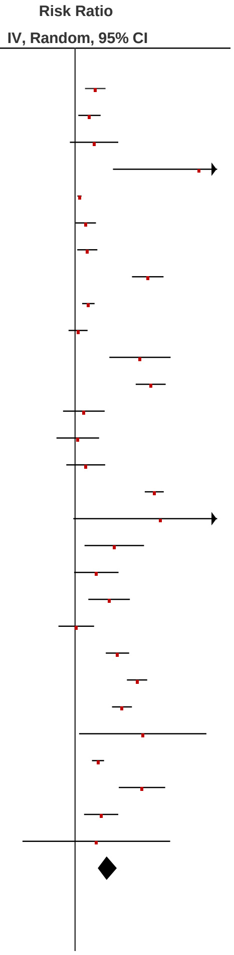

**1.18.2 developed countries**				
Bonuck 2006	-0.4384	0.4915	1.4%	0.65 [0.25, 1.69]
Forster 2004a	0.1738	0.2779	1.8%	1.19 [0.69, 2.05]
Forster 2004b	0.1481	0.2805	1.8%	1.16 [0.67, 2.01]
Frank 1987a	-0.7051	0.8521	0.9%	0.49 [0.09, 2.62]
Frank 1987b	0.3237	0.5634	1.3%	1.38 [0.46, 4.17]
Gijsbers 2006	0.5821	0.2933	1.8%	1.79 [1.01, 3.18]
Graffy 2004	0.1804	0.124	2.0%	1.20 [0.94, 1.53]
Kistin 1994	1.4183	0.5933	1.2%	4.13 [1.29, 13.21]
Kools 2005	-0.1583	0.1182	2.0%	0.85 [0.68, 1.08]
Kronborg 2007	0.4326	0.1986	1.9%	1.54 [1.04, 2.27]
Kruske 2007	0.9455	0.1998	1.9%	2.57 [1.74, 3.81]
Labarere 2003	-0.032	0.3568	1.6%	0.97 [0.48, 1.95]
Mattar 2007a	0.7472	0.3881	1.6%	2.11 [0.99, 4.52]
Mattar 2007b	-0.0525	0.463	1.5%	0.95 [0.38, 2.35]
McDonald 2008	0.0491	0.1524	1.9%	1.05 [0.78, 1.42]
McInnes 2000	0.4465	0.2503	1.8%	1.56 [0.96, 2.55]
McQueen 2011	0.1171	0.1802	1.9%	1.12 [0.79, 1.60]
Morrell 2000	0.0547	0.2406	1.8%	1.06 [0.66, 1.69]
Muirhead 2006	1.6182	1.5435	0.4%	5.04 [0.24, 103.90]
Noel-Weiss 2006	0.1156	0.1428	1.9%	1.12 [0.85, 1.49]
Nommsen-Rivers 2009	0.2868	0.2839	1.8%	1.33 [0.76, 2.32]
Petrova 2009	0.2772	0.6292	1.2%	1.32 [0.38, 4.53]
Pugh 2002	0.6444	0.6343	1.2%	1.90 [0.55, 6.60]
Ryan 2006	-0.862	0.0379	2.0%	0.42 [0.39, 0.45]
Sciacca 1995	0.8977	0.4669	1.5%	2.45 [0.98, 6.13]
Simonetti 2012	0.7634	0.4233	1.5%	2.15 [0.94, 4.92]
Sjolin 1979	0.1671	0.3771	1.6%	1.18 [0.56, 2.47]
Su 2007a	0.7503	0.3464	1.7%	2.12 [1.07, 4.18]
Su 2007b	0.7699	0.3439	1.7%	2.16 [1.10, 4.24]
Taveras 2011	-0.0715	0.2554	1.8%	0.93 [0.56, 1.54]
Wallace 2006	-0.0351	0.5235	1.4%	0.97 [0.35, 2.69]
Wen 2011	0.711	0.4928	1.4%	2.04 [0.78, 5.35]
Wrenn 1997	0.0367	0.1889	1.9%	1.04 [0.72, 1.50]
**Subtotal (95% CI)**			**52.9%**	**1.31 [1.00, 1.70]**
**Total (95% CI)**			**100.0%**	**1.90 [1.54, 2.34]**

Subtotal: Heterogeneity: Tau² = 0.45; Chi² = 387.42, df = 32 (P < 0.00001); I² = 92%

Subtotal: Test for overall effect: Z = 2.00 (P = 0.05)

Total: Heterogeneity: Tau² = 0.57; Chi² = 1629.52, df = 61 (P < 0.00001); I² = 96%

Total: Test for overall effect: Z = 5.97 (P < 0.00001)

Test for subgroup differences: Chi² = 14.69, df = 1 (P = 0.0001), I² = 93.2%

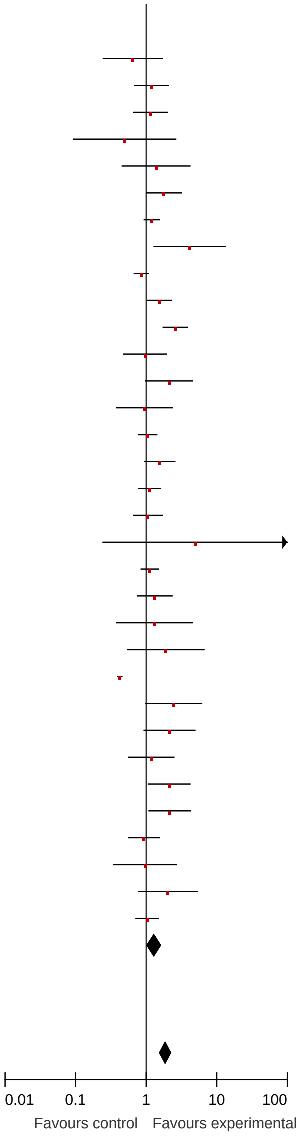

Figure 3d. Effect of breastfeeding education on the rate of exclusive breastfeeding at 6-12 months.

Study or Subgroup	log[]	SE	Weight	IV, Random, 95% CI
Bonuck 2006	0.1884	0.6146	100.0%	1.21 [0.36, 4.03]
**Total (95% CI)**			**100.0%**	**1.21 [0.36, 4.03]**

Heterogeneity: Not applicable

Test for overall effect: Z = 0.31 (P = 0.76)

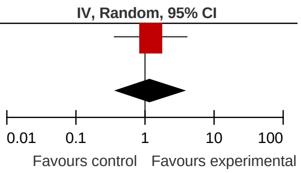

Figure 4a. Effect of breastfeeding education on the rate of predominant breastfeeding at 0-1 month: individual vs group counseling.

Study or Subgroup	log[Risk Ratio]	SE	Weight	Risk Ratio IV, Fixed, 95% CI
**1.26.2 individual counseling**				
Dennis 2002	-0.468	0.6333	11.8%	0.63 [0.18, 2.17]
Kupratakul 2010	-0.0513	0.4841	20.2%	0.95 [0.37, 2.45]
McQueen 2011	1.2964	1.1423	3.6%	3.66 [0.39, 34.30]
Simonetti 2012	-3.2268	1.4281	2.3%	0.04 [0.00, 0.65]
Tahir 2012	-0.5362	0.3842	32.1%	0.58 [0.28, 1.24]
Wong 2007	-0.5108	0.3972	30.0%	0.60 [0.28, 1.31]
**Subtotal (95% CI)**			**100.0%**	**0.66 [0.43, 1.01]**
Heterogeneity: Chi² = 6.85, df = 5 (P = 0.23); I² = 27%				
Test for overall effect: Z = 1.92 (P = 0.05)				
**Total (95% CI)**			**100.0%**	**0.66 [0.43, 1.01]**
Heterogeneity: Chi² = 6.85, df = 5 (P = 0.23); I² = 27%				
Test for overall effect: Z = 1.92 (P = 0.05)				
Test for subgroup differences: Not applicable				

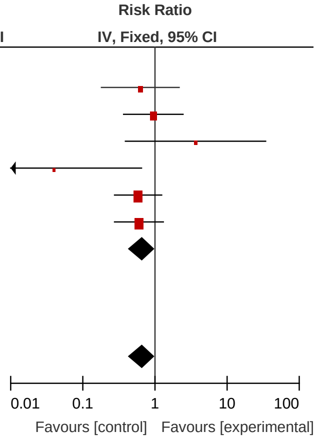

Figure 4b. Effect of breastfeeding education on the rate of predominant breastfeeding at 0-1 month: community- vs facility-based interventions.

Study or Subgroup	log[Risk Ratio]	SE	Weight	Risk Ratio IV, Fixed, 95% CI
**1.28.4 community-based intervention**				
Dennis 2002	-0.468	0.6333	11.8%	0.63 [0.18, 2.17]
**Subtotal (95% CI)**			**11.8%**	**0.63 [0.18, 2.17]**
Heterogeneity: Not applicable				
Test for overall effect: Z = 0.74 (P = 0.46)				
**1.28.5 facility-based intervention**				
Kupratakul 2010	-0.0513	0.4841	20.2%	0.95 [0.37, 2.45]
McQueen 2011	1.2964	1.1423	3.6%	3.66 [0.39, 34.30]
Tahir 2012	-0.5362	0.3842	32.1%	0.58 [0.28, 1.24]
Wong 2007	-0.5108	0.3972	30.0%	0.60 [0.28, 1.31]
**Subtotal (95% CI)**			**85.9%**	**0.71 [0.45, 1.13]**
Heterogeneity: Chi² = 2.85, df = 3 (P = 0.41); I² = 0%				
Test for overall effect: Z = 1.43 (P = 0.15)				
**1.28.6 facility and community-based intervention**				
Simonetti 2012	-3.2268	1.4281	2.3%	0.04 [0.00, 0.65]
**Subtotal (95% CI)**			**2.3%**	**0.04 [0.00, 0.65]**
Heterogeneity: Not applicable				
Test for overall effect: Z = 2.26 (P = 0.02)				
**Total (95% CI)**			**100.0%**	**0.66 [0.43, 1.01]**
Heterogeneity: Chi² = 6.85, df = 5 (P = 0.23); I² = 27%				
Test for overall effect: Z = 1.92 (P = 0.05)				
Test for subgroup differences: Chi² = 4.00, df = 2 (P = 0.14), I² = 50.0%				

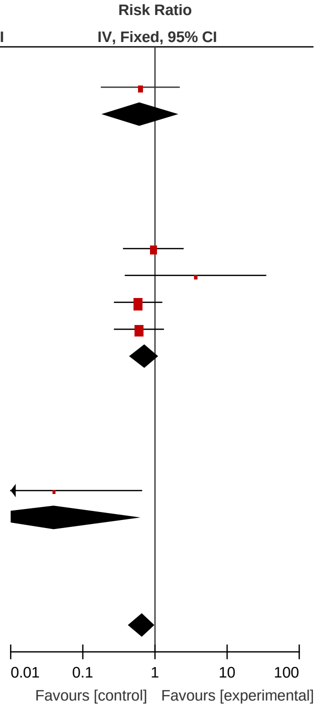

Figure 4c. Effect of breastfeeding education on the rate of predominant breastfeeding at 0-1 month: developing vs developed countries.

Study or Subgroup	log[Risk Ratio]	SE	Weight	Risk Ratio IV, Fixed, 95% CI
**1.30.1 developing countries**				
Kupratakul 2010	-0.0513	0.4841	20.2%	0.95 [0.37, 2.45]
Tahir 2012	-0.5362	0.3842	32.1%	0.58 [0.28, 1.24]
Wong 2007	-0.5108	0.3972	30.0%	0.60 [0.28, 1.31]
**Subtotal (95% CI)**			**82.3%**	**0.67 [0.42, 1.06]**
Heterogeneity: Chi² = 0.72, df = 2 (P = 0.70); I² = 0%				
Test for overall effect: Z = 1.70 (P = 0.09)				
**1.30.2 developed countries**				
Dennis 2002	-0.468	0.6333	11.8%	0.63 [0.18, 2.17]
McQueen 2011	1.2964	1.1423	3.6%	3.66 [0.39, 34.30]
Simonetti 2012	-3.2268	1.4281	2.3%	0.04 [0.00, 0.65]
**Subtotal (95% CI)**			**17.7%**	**0.63 [0.23, 1.72]**
Heterogeneity: Chi² = 6.12, df = 2 (P = 0.05); I² = 67%				
Test for overall effect: Z = 0.91 (P = 0.36)				
**Total (95% CI)**			**100.0%**	**0.66 [0.43, 1.01]**
Heterogeneity: Chi² = 6.85, df = 5 (P = 0.23); I² = 27%				
Test for overall effect: Z = 1.92 (P = 0.05)				
Test for subgroup differences: Chi² = 0.01, df = 1 (P = 0.92), I² = 0%				

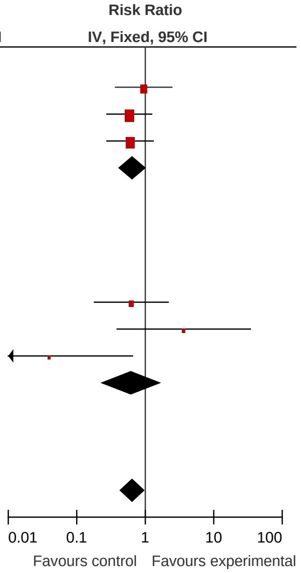

Figure 5a. Effect of breastfeeding education on the rate of predominant breastfeeding at 1-6 months: individual vs group counseling.

Study or Subgroup	log[Risk Ratio]	SE	Weight	Risk Ratio IV, Random, 95% CI
**1.32.2 individual and group counseling**				
Bhandari 2003	-1.4435	0.1915	10.5%	0.24 [0.16, 0.34]
Dearden 2002	-0.0254	0.1169	10.7%	0.97 [0.78, 1.23]
**Subtotal (95% CI)**			**21.2%**	**0.48 [0.12, 1.94]**
Heterogeneity: Tau² = 0.98; Chi² = 39.95, df = 1 (P < 0.00001); I² = 97%				
Test for overall effect: Z = 1.02 (P = 0.31)				
**1.32.3 individual counseling**				
Dennis 2002	-2.2597	1.0466	5.5%	0.10 [0.01, 0.81]
Froozani 1999	-0.9475	0.285	10.1%	0.39 [0.22, 0.68]
Jakobsen 1999	0.2243	0.1125	10.7%	1.25 [1.00, 1.56]
Kramer 2001	1.8904	0.092	10.8%	6.62 [5.53, 7.93]
Kupratakul 2010	2.0281	0.715	7.4%	7.60 [1.87, 30.86]
McDonald 2008	0.0183	0.0796	10.8%	1.02 [0.87, 1.19]
McQueen 2011	0.6904	0.7094	7.5%	1.99 [0.50, 8.01]
Simonetti 2012	0	0		Not estimable
Tahir 2012	0.3999	0.9067	6.2%	1.49 [0.25, 8.82]
Wong 2007	-0.0645	0.3304	9.9%	0.94 [0.49, 1.79]
**Subtotal (95% CI)**			**78.8%**	**1.34 [0.62, 2.90]**
Heterogeneity: Tau² = 1.13; Chi² = 306.24, df = 8 (P < 0.00001); I² = 97%				
Test for overall effect: Z = 0.74 (P = 0.46)				
**1.32.4 group counseling**				
Noel-Weiss 2006	0	0		Not estimable
**Subtotal (95% CI)**				**Not estimable**
Heterogeneity: Not applicable				
Test for overall effect: Not applicable				
**Total (95% CI)**			**100.0%**	**1.08 [0.55, 2.13]**
Heterogeneity: Tau² = 1.12; Chi² = 429.93, df = 10 (P < 0.00001); I² = 98%				
Test for overall effect: Z = 0.22 (P = 0.83)				
Test for subgroup differences: Chi² = 1.58, df = 1 (P = 0.21), I² = 36.6%				

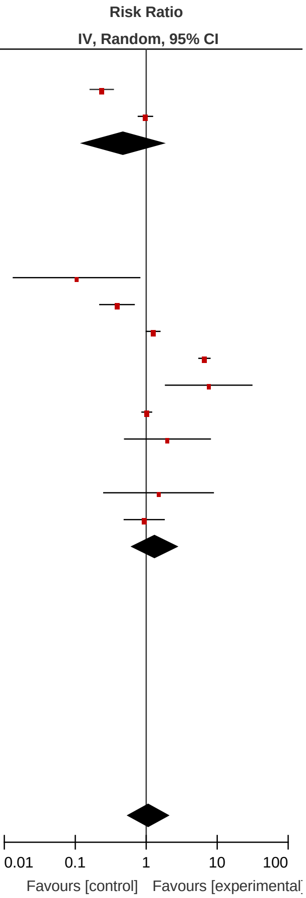

Figure 5b. Effect of breastfeeding education on the rate of predominant breastfeeding at 1-6 months: community- vs facility-based interventions.

Study or Subgroup	log[Risk Ratio]	SE	Weight	Risk Ratio IV, Random, 95% CI
**1.34.4 community-based intervention**				
Bhandari 2003	-1.4435	0.1915	10.5%	0.24 [0.16, 0.34]
Dearden 2002	-0.0254	0.1169	10.7%	0.97 [0.78, 1.23]
Dennis 2002	-2.2597	1.0466	5.5%	0.10 [0.01, 0.81]
**Subtotal (95% CI)**			**26.7%**	**0.36 [0.10, 1.26]**
Heterogeneity: Tau² = 1.00; Chi² = 43.04, df = 2 (P < 0.00001); I² = 95%				
Test for overall effect: Z = 1.60 (P = 0.11)				
**1.34.5 facility-based intervention**				
Kramer 2001	1.8904	0.092	10.8%	6.62 [5.53, 7.93]
Kupratakul 2010	2.0281	0.715	7.4%	7.60 [1.87, 30.86]
McDonald 2008	0.0183	0.0796	10.8%	1.02 [0.87, 1.19]
McQueen 2011	0.6904	0.7094	7.5%	1.99 [0.50, 8.01]
Noel-Weiss 2006	0	0		Not estimable
Tahir 2012	0.3999	0.9067	6.2%	1.49 [0.25, 8.82]
Wong 2007	-0.0645	0.3304	9.9%	0.94 [0.49, 1.79]
**Subtotal (95% CI)**			**52.5%**	**2.26 [0.77, 6.67]**
Heterogeneity: Tau² = 1.55; Chi² = 246.93, df = 5 (P < 0.00001); I² = 98%				
Test for overall effect: Z = 1.48 (P = 0.14)				
**1.34.7 facility and community-based intervention**				
Froozani 1999	-0.9475	0.285	10.1%	0.39 [0.22, 0.68]
Jakobsen 1999	0.2243	0.1125	10.7%	1.25 [1.00, 1.56]
Simonetti 2012	0	0		Not estimable
**Subtotal (95% CI)**			**20.8%**	**0.72 [0.23, 2.26]**
Heterogeneity: Tau² = 0.64; Chi² = 14.63, df = 1 (P = 0.0001); I² = 93%				
Test for overall effect: Z = 0.57 (P = 0.57)				
**Total (95% CI)**			**100.0%**	**1.08 [0.55, 2.13]**
Heterogeneity: Tau² = 1.12; Chi² = 429.93, df = 10 (P < 0.00001); I² = 98%				
Test for overall effect: Z = 0.22 (P = 0.83)				
Test for subgroup differences: Chi² = 5.00, df = 2 (P = 0.08), I² = 60.0%				

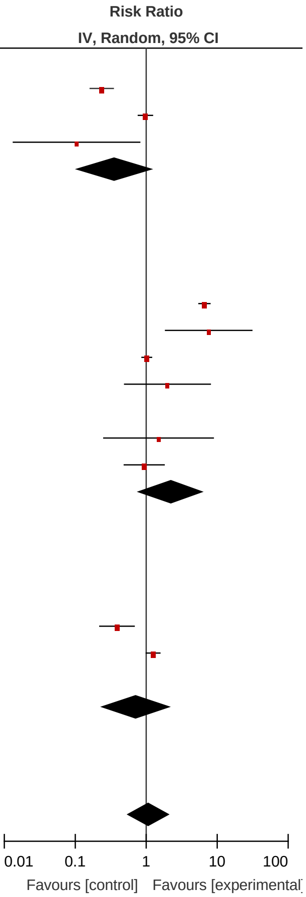

Figure 5c. Effect of breastfeeding education on the rate of predominant breastfeeding at 1-6 months: developing vs developed countries.

Study or Subgroup	log[Risk Ratio]	SE	Weight	Risk Ratio IV, Random, 95% CI
**1.36.1 developing countries**				
Bhandari 2003	-1.4435	0.1915	10.5%	0.24 [0.16, 0.34]
Dearden 2002	-0.0254	0.1169	10.7%	0.97 [0.78, 1.23]
Froozani 1999	-0.9475	0.285	10.1%	0.39 [0.22, 0.68]
Jakobsen 1999	0.2243	0.1125	10.7%	1.25 [1.00, 1.56]
Kramer 2001	1.8904	0.092	10.8%	6.62 [5.53, 7.93]
Kupratakul 2010	2.0281	0.715	7.4%	7.60 [1.87, 30.86]
Tahir 2012	0.3999	0.9067	6.2%	1.49 [0.25, 8.82]
Wong 2007	-0.0645	0.3304	9.9%	0.94 [0.49, 1.79]
**Subtotal (95% CI)**			**76.3%**	**1.23 [0.49, 3.08]**
Heterogeneity: Tau² = 1.59; Chi² = 387.94, df = 7 (P < 0.00001); I² = 98%				
Test for overall effect: Z = 0.44 (P = 0.66)				
**1.36.2 developed countries**				
Dennis 2002	-2.2597	1.0466	5.5%	0.10 [0.01, 0.81]
McDonald 2008	0.0183	0.0796	10.8%	1.02 [0.87, 1.19]
McQueen 2011	0.6904	0.7094	7.5%	1.99 [0.50, 8.01]
Noel-Weiss 2006	0	0		Not estimable
Simonetti 2012	0	0		Not estimable
**Subtotal (95% CI)**			**23.7%**	**0.80 [0.26, 2.48]**
Heterogeneity: Tau² = 0.64; Chi² = 5.63, df = 2 (P = 0.06); I² = 64%				
Test for overall effect: Z = 0.39 (P = 0.70)				
**Total (95% CI)**			**100.0%**	**1.08 [0.55, 2.13]**
Heterogeneity: Tau² = 1.12; Chi² = 429.93, df = 10 (P < 0.00001); I² = 98%				
Test for overall effect: Z = 0.22 (P = 0.83)				
Test for subgroup differences: Chi² = 0.34, df = 1 (P = 0.56), I² = 0%				

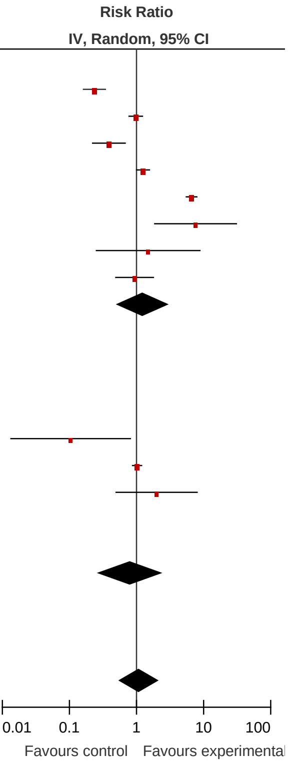

Figure 6a. Effect of breastfeeding education on the rate of partial breastfeeding at day 1: individual vs group counseling.

Study or Subgroup	log[Risk Ratio]	SE	Weight	Risk Ratio IV, Random, 95% CI
**1.38.6 individual and group counseling**				
Sciacca 1995	-0.7381	0.635	8.8%	0.48 [0.14, 1.66]
**Subtotal (95% CI)**			**8.8%**	**0.48 [0.14, 1.66]**
Heterogeneity: Not applicable				
Test for overall effect: Z = 1.16 (P = 0.25)				
**1.38.8 individual counseling**				
Huang 2007	-0.1769	0.1611	27.7%	0.84 [0.61, 1.15]
Kools 2005	0.4776	0.3021	20.2%	1.61 [0.89, 2.91]
McInnes 2000	1.4468	0.5551	10.6%	4.25 [1.43, 12.61]
Nommsen-Rivers 2009	0.0974	0.2588	22.5%	1.10 [0.66, 1.83]
Pisacane 2005	0.3365	0.5732	10.2%	1.40 [0.46, 4.31]
**Subtotal (95% CI)**			**91.2%**	**1.32 [0.84, 2.08]**
Heterogeneity: Tau² = 0.15; Chi² = 10.54, df = 4 (P = 0.03); I² = 62%				
Test for overall effect: Z = 1.22 (P = 0.22)				
**Total (95% CI)**			**100.0%**	**1.21 [0.79, 1.87]**
Heterogeneity: Tau² = 0.15; Chi² = 12.14, df = 5 (P = 0.03); I² = 59%				
Test for overall effect: Z = 0.87 (P = 0.38)				
Test for subgroup differences: Chi² = 2.28, df = 1 (P = 0.13), I² = 56.1%				

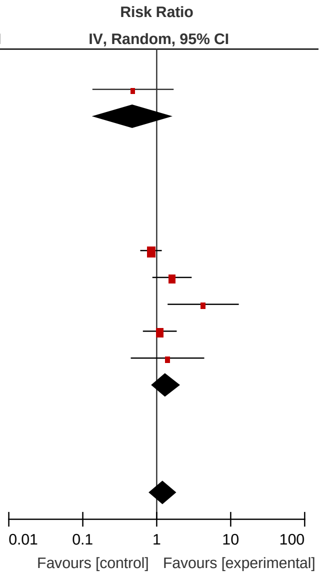

Figure 6b. Effect of breastfeeding education on the rate of partial breastfeeding at day 1: community- vs facility-based interventions.

Study or Subgroup	log[Risk Ratio]	SE	Weight	Risk Ratio IV, Random, 95% CI
**1.40.4 community-based intervention**				
McInnes 2000	1.4468	0.5551	10.6%	4.25 [1.43, 12.61]
**Subtotal (95% CI)**			**10.6%**	**4.25 [1.43, 12.61]**
Heterogeneity: Not applicable				
Test for overall effect: Z = 2.61 (P = 0.009)				
**1.40.5 facility-based intervention**				
Huang 2007	-0.1769	0.1611	27.7%	0.84 [0.61, 1.15]
Pisacane 2005	0.3365	0.5732	10.2%	1.40 [0.46, 4.31]
Sciacca 1995	-0.7381	0.635	8.8%	0.48 [0.14, 1.66]
**Subtotal (95% CI)**			**46.7%**	**0.84 [0.63, 1.13]**
Heterogeneity: Tau² = 0.00; Chi² = 1.58, df = 2 (P = 0.45); I² = 0%				
Test for overall effect: Z = 1.15 (P = 0.25)				
**1.40.7 facility and community-based intervention**				
Kools 2005	0.4776	0.3021	20.2%	1.61 [0.89, 2.91]
Nommsen-Rivers 2009	0.0974	0.2588	22.5%	1.10 [0.66, 1.83]
**Subtotal (95% CI)**			**42.7%**	**1.29 [0.88, 1.90]**
Heterogeneity: Tau² = 0.00; Chi² = 0.91, df = 1 (P = 0.34); I² = 0%				
Test for overall effect: Z = 1.31 (P = 0.19)				
**Total (95% CI)**			**100.0%**	**1.21 [0.79, 1.87]**
Heterogeneity: Tau² = 0.15; Chi² = 12.14, df = 5 (P = 0.03); I² = 59%				
Test for overall effect: Z = 0.87 (P = 0.38)				
Test for subgroup differences: Chi² = 9.65, df = 2 (P = 0.008), I² = 79.3%				

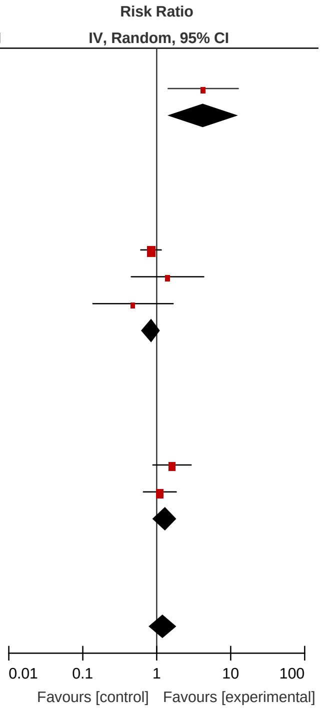

Figure 6c. Effect of breastfeeding education on the rate of partial breastfeeding at day 1: developing vs developed countries.

Study or Subgroup	log[]	SE	Weight	IV, Random, 95% CI
**1.42.1 developing countries**				
Huang 2007	-0.1769	0.1611	27.7%	0.84 [0.61, 1.15]
**Subtotal (95% CI)**			**27.7%**	**0.84 [0.61, 1.15]**
Heterogeneity: Not applicable				
Test for overall effect: Z = 1.10 (P = 0.27)				
**1.42.2 developed countries**				
Kools 2005	0.4776	0.3021	20.2%	1.61 [0.89, 2.91]
McInnes 2000	1.4468	0.5551	10.6%	4.25 [1.43, 12.61]
Nommsen-Rivers 2009	0.0974	0.2588	22.5%	1.10 [0.66, 1.83]
Pisacane 2005	0.3365	0.5732	10.2%	1.40 [0.46, 4.31]
Sciacca 1995	-0.7381	0.635	8.8%	0.48 [0.14, 1.66]
**Subtotal (95% CI)**			**72.3%**	**1.40 [0.83, 2.35]**
Heterogeneity: Tau² = 0.16; Chi² = 7.90, df = 4 (P = 0.10); I² = 49%				
Test for overall effect: Z = 1.26 (P = 0.21)				
**Total (95% CI)**			**100.0%**	**1.21 [0.79, 1.87]**
Heterogeneity: Tau² = 0.15; Chi² = 12.14, df = 5 (P = 0.03); I² = 59%				
Test for overall effect: Z = 0.87 (P = 0.38)				
Test for subgroup differences: Chi² = 2.72, df = 1 (P = 0.10), I² = 63.2%				

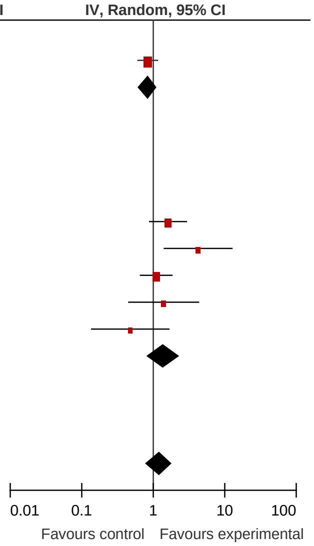

Figure 7a. Effect of breastfeeding education on the rate of partial breastfeeding at 0-1 month: individual vs group counseling.

Study or Subgroup	log[Risk Ratio]	SE	Weight	Risk Ratio IV, Fixed, 95% CI
**1.44.6 individual and group counseling**				
Alvarado 1996	-3.3724	1.4261	0.5%	0.03 [0.00, 0.56]
Sciacca 1995	-0.2963	0.5863	3.2%	0.74 [0.24, 2.35]
**Subtotal (95% CI)**			**3.8%**	**0.48 [0.16, 1.38]**
Heterogeneity: Chi² = 3.98, df = 1 (P = 0.05); I² = 75%				
Test for overall effect: Z = 1.37 (P = 0.17)				
**1.44.8 individual counseling**				
Dennis 2002	-0.1197	0.3142	11.2%	0.89 [0.48, 1.64]
Huang 2007	0.0896	0.1628	41.8%	1.09 [0.79, 1.50]
Kupratakul 2010	-1.3041	0.7691	1.9%	0.27 [0.06, 1.23]
McQueen 2011	0.1237	0.3463	9.2%	1.13 [0.57, 2.23]
Porteous 2000	-0.7324	0.5446	3.7%	0.48 [0.17, 1.40]
Ryser 2004	-0.3314	0.5852	3.2%	0.72 [0.23, 2.26]
Simonetti 2012	-0.3606	0.303	12.1%	0.70 [0.39, 1.26]
Tahir 2012	-0.5164	0.4081	6.6%	0.60 [0.27, 1.33]
Wong 2007	0.0953	0.4134	6.5%	1.10 [0.49, 2.47]
**Subtotal (95% CI)**			**96.2%**	**0.90 [0.73, 1.11]**
Heterogeneity: Chi² = 7.74, df = 8 (P = 0.46); I² = 0%				
Test for overall effect: Z = 0.96 (P = 0.34)				
**1.44.9 group counseling**				
**Subtotal (95% CI)**				**Not estimable**
Heterogeneity: Not applicable				
Test for overall effect: Not applicable				
**Total (95% CI)**			**100.0%**	**0.88 [0.72, 1.08]**
Heterogeneity: Chi² = 13.05, df = 10 (P = 0.22); I² = 23%				
Test for overall effect: Z = 1.20 (P = 0.23)				
Test for subgroup differences: Chi² = 1.33, df = 1 (P = 0.25), I² = 25.1%				

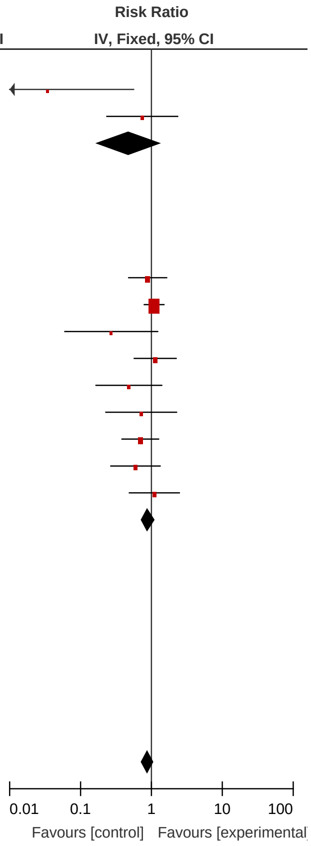

Figure 7b. Effect of breastfeeding education on the rate of partial breastfeeding at 0-1 month: community- vs facility-based interventions.

Study or Subgroup	log[Risk Ratio]	SE	Weight	Risk Ratio IV, Fixed, 95% CI
**1.46.4 community-based intervention**				
Dennis 2002	-0.1197	0.3142	11.2%	0.89 [0.48, 1.64]
**Subtotal (95% CI)**			**11.2%**	**0.89 [0.48, 1.64]**
Heterogeneity: Not applicable				
Test for overall effect: Z = 0.38 (P = 0.70)				
**1.46.5 facility-based intervention**				
Huang 2007	0.0896	0.1628	41.8%	1.09 [0.79, 1.50]
Kupratakul 2010	-1.3041	0.7691	1.9%	0.27 [0.06, 1.23]
McQueen 2011	0.1237	0.3463	9.2%	1.13 [0.57, 2.23]
Ryser 2004	-0.3314	0.5852	3.2%	0.72 [0.23, 2.26]
Sciacca 1995	-0.2963	0.5863	3.2%	0.74 [0.24, 2.35]
Tahir 2012	-0.5164	0.4081	6.6%	0.60 [0.27, 1.33]
Wong 2007	0.0953	0.4134	6.5%	1.10 [0.49, 2.47]
**Subtotal (95% CI)**			**72.5%**	**0.97 [0.76, 1.23]**
Heterogeneity: $Chi^2 = 5.46$, df = 6 (P = 0.49); $I^2 = 0\%$				
Test for overall effect: Z = 0.27 (P = 0.79)				
**1.46.7 facility and community-based intervention**				
Alvarado 1996	-3.3724	1.4261	0.5%	0.03 [0.00, 0.56]
Porteous 2000	-0.7324	0.5446	3.7%	0.48 [0.17, 1.40]
Simonetti 2012	-0.3606	0.303	12.1%	0.70 [0.39, 1.26]
**Subtotal (95% CI)**			**16.3%**	**0.58 [0.35, 0.96]**
Heterogeneity: $Chi^2 = 4.42$, df = 2 (P = 0.11); $I^2 = 55\%$				
Test for overall effect: Z = 2.10 (P = 0.04)				
**Total (95% CI)**			**100.0%**	**0.88 [0.72, 1.08]**
Heterogeneity: $Chi^2 = 13.05$, df = 10 (P = 0.22); $I^2 = 23\%$				
Test for overall effect: Z = 1.20 (P = 0.23)				
Test for subgroup differences: $Chi^2 = 3.17$, df = 2 (P = 0.21), $I^2 = 36.9\%$				

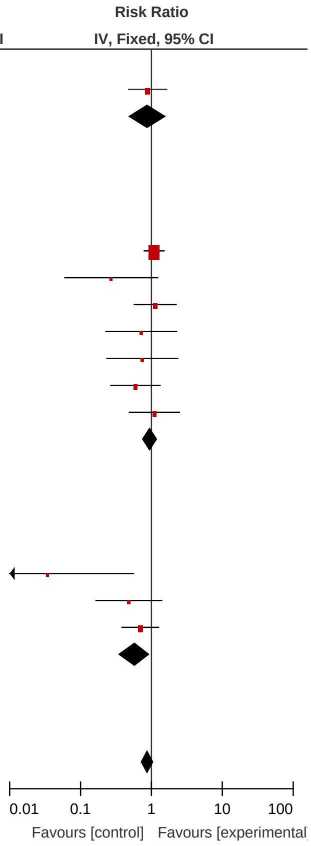

Figure 7c. Effect of breastfeeding education on the rate of partial breastfeeding at 0-1 month: developing vs developed countries.

Study or Subgroup	log[Risk Ratio]	SE	Weight	Risk Ratio IV, Fixed, 95% CI
**1.48.1 developing countries**				
Alvarado 1996	-3.3724	1.4261	0.5%	0.03 [0.00, 0.56]
Huang 2007	0.0896	0.1628	41.8%	1.09 [0.79, 1.50]
Kupratakul 2010	-1.3041	0.7691	1.9%	0.27 [0.06, 1.23]
Tahir 2012	-0.5164	0.4081	6.6%	0.60 [0.27, 1.33]
Wong 2007	0.0953	0.4134	6.5%	1.10 [0.49, 2.47]
**Subtotal (95% CI)**			**57.3%**	**0.94 [0.72, 1.24]**
Heterogeneity: Chi² = 10.25, df = 4 (P = 0.04); I² = 61%				
Test for overall effect: Z = 0.42 (P = 0.67)				
**1.48.2 developed countries**				
Dennis 2002	-0.1197	0.3142	11.2%	0.89 [0.48, 1.64]
McQueen 2011	0.1237	0.3463	9.2%	1.13 [0.57, 2.23]
Porteous 2000	-0.7324	0.5446	3.7%	0.48 [0.17, 1.40]
Ryser 2004	-0.3314	0.5852	3.2%	0.72 [0.23, 2.26]
Sciacca 1995	-0.2963	0.5863	3.2%	0.74 [0.24, 2.35]
Simonetti 2012	-0.3606	0.303	12.1%	0.70 [0.39, 1.26]
**Subtotal (95% CI)**			**42.7%**	**0.80 [0.59, 1.10]**
Heterogeneity: Chi² = 2.24, df = 5 (P = 0.81); I² = 0%				
Test for overall effect: Z = 1.35 (P = 0.18)				
**Total (95% CI)**			**100.0%**	**0.88 [0.72, 1.08]**
Heterogeneity: Chi² = 13.05, df = 10 (P = 0.22); I² = 23%				
Test for overall effect: Z = 1.20 (P = 0.23)				
Test for subgroup differences: Chi² = 0.56, df = 1 (P = 0.45), I² = 0%				

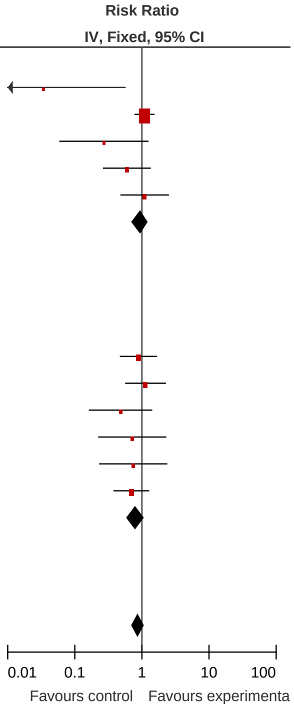

Figure 8a. Effect of breastfeeding education on the rate of partial breastfeeding at 1-6 months: individual vs group counseling.

Study or Subgroup	log[Risk Ratio]	SE	Weight	Risk Ratio IV, Random, 95% CI
**1.50.6 individual and group counseling**				
Alvarado 1996	-0.0957	0.1472	9.2%	0.91 [0.68, 1.21]
Bhandari 2003	-0.5267	0.1495	9.1%	0.59 [0.44, 0.79]
Sciacca 1995	1.0255	0.7919	0.9%	2.79 [0.59, 13.17]
**Subtotal (95% CI)**			**19.2%**	**0.82 [0.51, 1.31]**
Heterogeneity: Tau² = 0.10; Chi² = 7.01, df = 2 (P = 0.03); I² = 71%				
Test for overall effect: Z = 0.85 (P = 0.40)				
**1.50.8 individual counseling**				
Dennis 2002	0.1693	0.2496	5.7%	1.18 [0.73, 1.93]
Froozani 1999	-0.1208	0.3485	3.7%	0.89 [0.45, 1.75]
Huang 2007	0.3302	0.2035	7.1%	1.39 [0.93, 2.07]
Khresheh 2011	-0.7732	0.4463	2.5%	0.46 [0.19, 1.11]
Kools 2005	-0.109	0.307	4.4%	0.90 [0.49, 1.64]
Kupratakul 2010	-0.0513	0.5311	1.9%	0.95 [0.34, 2.69]
McInnes 2000	-0.5517	0.5032	2.0%	0.58 [0.21, 1.54]
McQueen 2011	-0.3594	0.4428	2.5%	0.70 [0.29, 1.66]
Morrell 2000	-0.1609	0.3074	4.4%	0.85 [0.47, 1.56]
Pisacane 2005	-0.0247	0.1874	7.6%	0.98 [0.68, 1.41]
Simonetti 2012	0.0702	0.2277	6.3%	1.07 [0.69, 1.68]
Tahir 2012	0.0468	0.0615	12.6%	1.05 [0.93, 1.18]
Valdes 2000	-0.5122	0.1505	9.0%	0.60 [0.45, 0.80]
Wong 2007	-1.0986	1.6269	0.2%	0.33 [0.01, 8.09]
Wrenn 1997	0.0892	0.2651	5.3%	1.09 [0.65, 1.84]
**Subtotal (95% CI)**			**75.1%**	**0.93 [0.80, 1.09]**
Heterogeneity: Tau² = 0.02; Chi² = 21.11, df = 14 (P = 0.10); I² = 34%				
Test for overall effect: Z = 0.90 (P = 0.37)				
**1.50.9 group counseling**				
Kruske 2007	-0.7728	0.3327	3.9%	0.46 [0.24, 0.89]
Noel-Weiss 2006	-0.0435	0.5384	1.8%	0.96 [0.33, 2.75]
**Subtotal (95% CI)**			**5.7%**	**0.59 [0.30, 1.15]**
Heterogeneity: Tau² = 0.07; Chi² = 1.33, df = 1 (P = 0.25); I² = 25%				
Test for overall effect: Z = 1.55 (P = 0.12)				
**Total (95% CI)**			**100.0%**	**0.87 [0.75, 1.02]**
Heterogeneity: Tau² = 0.04; Chi² = 37.89, df = 19 (P = 0.006); I² = 50%				
Test for overall effect: Z = 1.77 (P = 0.08)				
Test for subgroup differences: Chi² = 1.91, df = 2 (P = 0.39), I² = 0%				

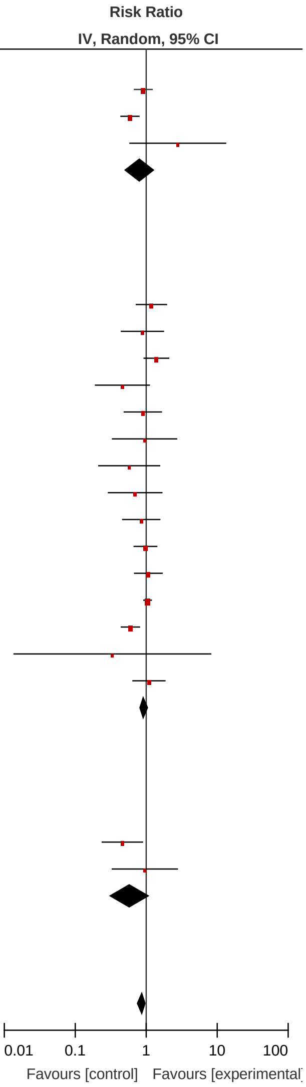

Figure 8b. Effect of breastfeeding education on the rate of partial breastfeeding at 1-6 months: community- vs facility-based interventions.

Study or Subgroup	log[Risk Ratio]	SE	Weight	Risk Ratio IV, Random, 95% CI
**1.52.4 community-based intervention**				
Bhandari 2003	-0.5267	0.1495	9.1%	0.59 [0.44, 0.79]
Dennis 2002	0.1693	0.2496	5.7%	1.18 [0.73, 1.93]
Kruske 2007	-0.7728	0.3327	3.9%	0.46 [0.24, 0.89]
McInnes 2000	-0.5517	0.5032	2.0%	0.58 [0.21, 1.54]
Morrell 2000	-0.1609	0.3074	4.4%	0.85 [0.47, 1.56]
**Subtotal (95% CI)**			**25.1%**	**0.71 [0.50, 1.00]**
Heterogeneity: Tau² = 0.07; Chi² = 7.82, df = 4 (P = 0.10); I² = 49%				
Test for overall effect: Z = 1.98 (P = 0.05)				
**1.52.5 facility-based intervention**				
Huang 2007	0.3302	0.2035	7.1%	1.39 [0.93, 2.07]
Khresheh 2011	-0.7732	0.4463	2.5%	0.46 [0.19, 1.11]
Kupratakul 2010	-0.0513	0.5311	1.9%	0.95 [0.34, 2.69]
McQueen 2011	-0.3594	0.4428	2.5%	0.70 [0.29, 1.66]
Noel-Weiss 2006	-0.0435	0.5384	1.8%	0.96 [0.33, 2.75]
Pisacane 2005	-0.0247	0.1874	7.6%	0.98 [0.68, 1.41]
Sciacca 1995	1.0255	0.7919	0.9%	2.79 [0.59, 13.17]
Tahir 2012	0.0468	0.0615	12.6%	1.05 [0.93, 1.18]
Valdes 2000	-0.5122	0.1505	9.0%	0.60 [0.45, 0.80]
Wong 2007	-1.0986	1.6269	0.2%	0.33 [0.01, 8.09]
**Subtotal (95% CI)**			**46.1%**	**0.91 [0.71, 1.16]**
Heterogeneity: Tau² = 0.06; Chi² = 20.46, df = 9 (P = 0.02); I² = 56%				
Test for overall effect: Z = 0.76 (P = 0.45)				
**1.52.7 facility and community-based intervention**				
Alvarado 1996	-0.0957	0.1472	9.2%	0.91 [0.68, 1.21]
Froozani 1999	-0.1208	0.3485	3.7%	0.89 [0.45, 1.75]
Kools 2005	-0.109	0.307	4.4%	0.90 [0.49, 1.64]
Simonetti 2012	0.0702	0.2277	6.3%	1.07 [0.69, 1.68]
Wrenn 1997	0.0892	0.2651	5.3%	1.09 [0.65, 1.84]
**Subtotal (95% CI)**			**28.8%**	**0.96 [0.79, 1.17]**
Heterogeneity: Tau² = 0.00; Chi² = 0.72, df = 4 (P = 0.95); I² = 0%				
Test for overall effect: Z = 0.40 (P = 0.69)				
**Total (95% CI)**			**100.0%**	**0.87 [0.75, 1.02]**
Heterogeneity: Tau² = 0.04; Chi² = 37.89, df = 19 (P = 0.006); I² = 50%				
Test for overall effect: Z = 1.77 (P = 0.08)				
Test for subgroup differences: Chi² = 2.32, df = 2 (P = 0.31), I² = 13.7%				

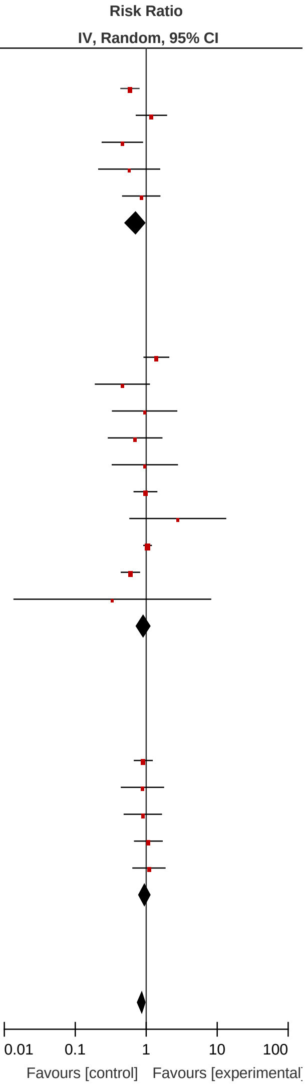

Figure 8c. Effect of breastfeeding education on the rate of partial breastfeeding at 1-6 months: developing vs developed countries.

Study or Subgroup	log[Risk Ratio]	SE	Weight	Risk Ratio IV, Random, 95% CI
**1.54.1 developing countries**				
Alvarado 1996	-0.0957	0.1472	9.2%	0.91 [0.68, 1.21]
Bhandari 2003	-0.5267	0.1495	9.1%	0.59 [0.44, 0.79]
Froozani 1999	-0.1208	0.3485	3.7%	0.89 [0.45, 1.75]
Huang 2007	0.3302	0.2035	7.1%	1.39 [0.93, 2.07]
Khresheh 2011	-0.7732	0.4463	2.5%	0.46 [0.19, 1.11]
Kupratakul 2010	-0.0513	0.5311	1.9%	0.95 [0.34, 2.69]
Tahir 2012	0.0468	0.0615	12.6%	1.05 [0.93, 1.18]
Valdes 2000	-0.5122	0.1505	9.0%	0.60 [0.45, 0.80]
Wong 2007	-1.0986	1.6269	0.2%	0.33 [0.01, 8.09]
**Subtotal (95% CI)**			**55.2%**	**0.83 [0.65, 1.06]**
Heterogeneity: Tau² = 0.08; Chi² = 28.32, df = 8 (P = 0.0004); I² = 72%				
Test for overall effect: Z = 1.52 (P = 0.13)				
**1.54.2 developed countries**				
Dennis 2002	0.1693	0.2496	5.7%	1.18 [0.73, 1.93]
Kools 2005	-0.109	0.307	4.4%	0.90 [0.49, 1.64]
Kruske 2007	-0.7728	0.3327	3.9%	0.46 [0.24, 0.89]
McInnes 2000	-0.5517	0.5032	2.0%	0.58 [0.21, 1.54]
McQueen 2011	-0.3594	0.4428	2.5%	0.70 [0.29, 1.66]
Morrell 2000	-0.1609	0.3074	4.4%	0.85 [0.47, 1.56]
Noel-Weiss 2006	-0.0435	0.5384	1.8%	0.96 [0.33, 2.75]
Pisacane 2005	-0.0247	0.1874	7.6%	0.98 [0.68, 1.41]
Sciacca 1995	1.0255	0.7919	0.9%	2.79 [0.59, 13.17]
Simonetti 2012	0.0702	0.2277	6.3%	1.07 [0.69, 1.68]
Wrenn 1997	0.0892	0.2651	5.3%	1.09 [0.65, 1.84]
**Subtotal (95% CI)**			**44.8%**	**0.94 [0.79, 1.12]**
Heterogeneity: Tau² = 0.00; Chi² = 9.54, df = 10 (P = 0.48); I² = 0%				
Test for overall effect: Z = 0.70 (P = 0.48)				
**Total (95% CI)**			**100.0%**	**0.87 [0.75, 1.02]**
Heterogeneity: Tau² = 0.04; Chi² = 37.89, df = 19 (P = 0.006); I² = 50%				
Test for overall effect: Z = 1.77 (P = 0.08)				
Test for subgroup differences: Chi² = 0.68, df = 1 (P = 0.41), I² = 0%				

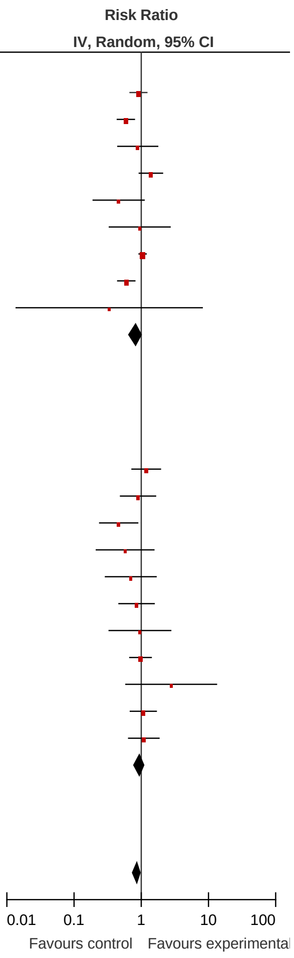

Figure 8d. Effect of breastfeeding education on the rate of partial breastfeeding at 6-12 months.

Study or Subgroup	log[Risk Ratio]	SE	Weight	Risk Ratio IV, Fixed, 95% CI
Arifeen 2009	0.1668	0.0309	98.9%	1.18 [1.11, 1.26]
Pisacane 2005	0.5232	0.292	1.1%	1.69 [0.95, 2.99]
**Total (95% CI)**			**100.0%**	**1.19 [1.12, 1.26]**
Heterogeneity: Chi² = 1.47, df = 1 (P = 0.22); I² = 32%				
Test for overall effect: Z = 5.56 (P < 0.00001)				

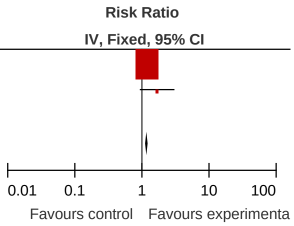

Figure 9a. Effect of breastfeeding education on the rate of no breastfeeding at day 1: individual vs group counseling.

Study or Subgroup	log[Risk Ratio]	SE	Weight	Risk Ratio IV, Random, 95% CI
**1.56.4 individual and group counseling**				
Bhandari 2003	-0.4153	0.0529	3.1%	0.66 [0.60, 0.73]
Dearden 2002	-0.0939	0.0405	3.1%	0.91 [0.84, 0.99]
Ingram 2005	0.8197	0.0683	3.1%	2.27 [1.99, 2.59]
Kumar 2008	-1.0571	0.0418	3.1%	0.35 [0.32, 0.38]
Pobocik 2000	-0.4218	0.1627	2.9%	0.66 [0.48, 0.90]
Sciacca 1995	-2.4596	1.4434	0.6%	0.09 [0.01, 1.45]
Su 2007b	-0.1542	0.5432	1.9%	0.86 [0.30, 2.49]
Valdes 1993	-1.3704	0.2194	2.8%	0.25 [0.17, 0.39]
Wambach 2011	-0.5132	0.2467	2.8%	0.60 [0.37, 0.97]
**Subtotal (95% CI)**			**23.4%**	**0.64 [0.39, 1.04]**
Heterogeneity: Tau² = 0.47; Chi² = 644.23, df = 8 (P < 0.00001); I² = 99%				
Test for overall effect: Z = 1.81 (P = 0.07)				
**1.56.5 individual counseling**				
Aksu 2011	0.4055	0.8756	1.2%	1.50 [0.27, 8.34]
Anderson 2005	-0.9079	0.4424	2.2%	0.40 [0.17, 0.96]
Bhutta 2008	-0.8445	0.0751	3.1%	0.43 [0.37, 0.50]
Brent 1995	-0.5566	0.1962	2.9%	0.57 [0.39, 0.84]
Chapman 2004	-0.9361	0.3992	2.3%	0.39 [0.18, 0.86]
Graffy 2004	0.2877	0.374	2.4%	1.33 [0.64, 2.78]
Haider 2000	-0.8476	0.0775	3.1%	0.43 [0.37, 0.50]
Kools 2005	0.1233	0.117	3.0%	1.13 [0.90, 1.42]
Long 1995	-0.6524	0.3887	2.4%	0.52 [0.24, 1.12]
MacArthur 2009	-0.0267	0.0607	3.1%	0.97 [0.86, 1.10]
Martens 2000	-0.0202	0.4706	2.1%	0.98 [0.39, 2.46]
McInnes 2000	-0.0469	0.0346	3.1%	0.95 [0.89, 1.02]
Morrow 1999	0.1476	0.2808	2.7%	1.16 [0.67, 2.01]
Mottl-Santiago 2008	-0.3556	0.0205	3.1%	0.70 [0.67, 0.73]
Muirhead 2006	-0.0296	0.1439	3.0%	0.97 [0.73, 1.29]
Nommsen-Rivers 2009	-0.1005	0.2347	2.8%	0.90 [0.57, 1.43]
Olson 2008	-0.559	0.0962	3.0%	0.57 [0.47, 0.69]
Pisacane 2005	-0.6061	0.4932	2.1%	0.55 [0.21, 1.43]
Quinlivan 2003	0.1349	0.2882	2.6%	1.14 [0.65, 2.01]
Rasmussen 2011	0	0		Not estimable
Rishel 2005	-3.6636	0.7049	1.5%	0.03 [0.01, 0.10]
Ryan 2006	0.6482	0.0064	3.1%	1.91 [1.89, 1.94]
Sandy 2009	-0.4515	0.2844	2.7%	0.64 [0.36, 1.11]
Serwint 1996	-0.1656	0.1276	3.0%	0.85 [0.66, 1.09]
Shaw 1999	-0.0313	0.0715	3.1%	0.97 [0.84, 1.11]
Su 2007a	-0.8179	0.6793	1.6%	0.44 [0.12, 1.67]
Taddei 2000	-0.1755	0.0399	3.1%	0.84 [0.78, 0.91]
**Subtotal (95% CI)**			**68.2%**	**0.73 [0.55, 0.96]**
Heterogeneity: Tau² = 0.43; Chi² = 3863.85, df = 25 (P < 0.00001); I² = 99%				
Test for overall effect: Z = 2.28 (P = 0.02)				
**1.56.6 group counseling**				
Rossiter 1994	-0.735	0.1757	2.9%	0.48 [0.34, 0.68]
Turan 2003	-0.3299	0.1157	3.0%	0.72 [0.57, 0.90]
Wolfberg 2004	-0.8286	0.3567	2.5%	0.44 [0.22, 0.88]
**Subtotal (95% CI)**			**8.4%**	**0.57 [0.41, 0.80]**
Heterogeneity: Tau² = 0.05; Chi² = 4.74, df = 2 (P = 0.09); I² = 58%				
Test for overall effect: Z = 3.29 (P = 0.0010)				
**Total (95% CI)**			**100.0%**	**0.68 [0.54, 0.87]**
Heterogeneity: Tau² = 0.48; Chi² = 5901.19, df = 37 (P < 0.00001); I² = 99%				
Test for overall effect: Z = 3.10 (P = 0.002)				
Test for subgroup differences: Chi² = 1.21, df = 2 (P = 0.55), I² = 0%				

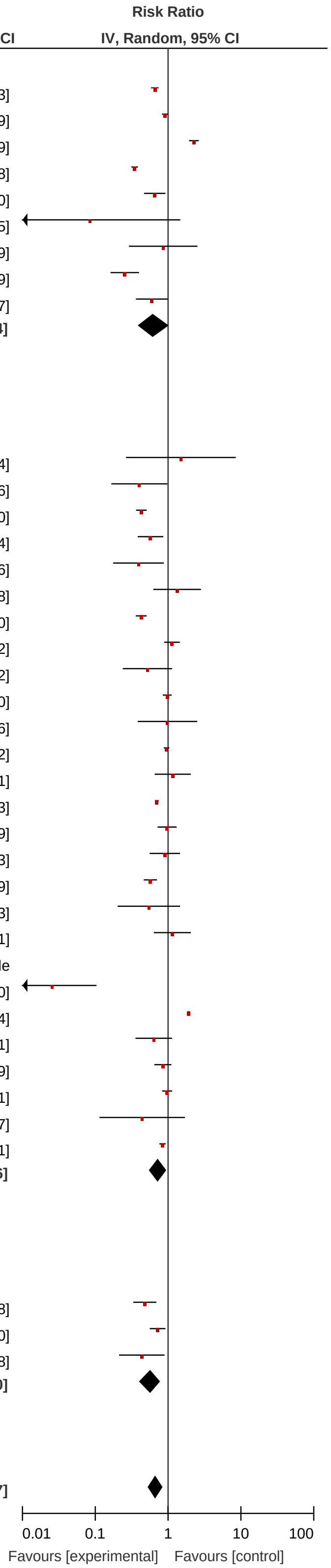

Figure 9b. Effect of breastfeeding education on the rate of no breastfeeding at day 1: community- vs facility-based interventions.

Study or Subgroup	log[Risk Ratio]	SE	Weight	Risk Ratio IV, Random, 95% CI
**1.58.7 community-based intervention**				
Bhandari 2003	-0.4153	0.0529	3.1%	0.66 [0.60, 0.73]
Bhutta 2008	-0.8445	0.0751	3.1%	0.43 [0.37, 0.50]
Dearden 2002	-0.0939	0.0405	3.1%	0.91 [0.84, 0.99]
Haider 2000	-0.8476	0.0775	3.1%	0.43 [0.37, 0.50]
Ingram 2005	0.8197	0.0683	3.1%	2.27 [1.99, 2.59]
Kumar 2008	-1.0571	0.0418	3.1%	0.35 [0.32, 0.38]
McInnes 2000	-0.0469	0.0346	3.1%	0.95 [0.89, 1.02]
Morrow 1999	0.1476	0.2808	2.7%	1.16 [0.67, 2.01]
Turan 2003	-0.3299	0.1157	3.0%	0.72 [0.57, 0.90]
**Subtotal (95% CI)**			**27.3%**	**0.74 [0.51, 1.07]**
Heterogeneity: Tau² = 0.31; Chi² = 783.87, df = 8 (P < 0.00001); I² = 99%				
Test for overall effect: Z = 1.61 (P = 0.11)				
**1.58.8 facility-based intervention**				
Brent 1995	-0.5566	0.1962	2.9%	0.57 [0.39, 0.84]
Martens 2000	-0.0202	0.4706	2.1%	0.98 [0.39, 2.46]
Pisacane 2005	-0.6061	0.4932	2.1%	0.55 [0.21, 1.43]
Rasmussen 2011	0	0		Not estimable
Rishel 2005	-3.6636	0.7049	1.5%	0.03 [0.01, 0.10]
Rossiter 1994	-0.735	0.1757	2.9%	0.48 [0.34, 0.68]
Sciacca 1995	-2.4596	1.4434	0.6%	0.09 [0.01, 1.45]
Serwint 1996	-0.1656	0.1276	3.0%	0.85 [0.66, 1.09]
Taddei 2000	-0.1755	0.0399	3.1%	0.84 [0.78, 0.91]
Valdes 1993	-1.3704	0.2194	2.8%	0.25 [0.17, 0.39]
Wolfberg 2004	-0.8286	0.3567	2.5%	0.44 [0.22, 0.88]
**Subtotal (95% CI)**			**23.5%**	**0.48 [0.34, 0.69]**
Heterogeneity: Tau² = 0.22; Chi² = 69.17, df = 9 (P < 0.00001); I² = 87%				
Test for overall effect: Z = 4.00 (P < 0.0001)				
**1.58.9 facility and community-based intervention**				
Aksu 2011	0.4055	0.8756	1.2%	1.50 [0.27, 8.34]
Anderson 2005	-0.9079	0.4424	2.2%	0.40 [0.17, 0.96]
Chapman 2004	-0.9361	0.3992	2.3%	0.39 [0.18, 0.86]
Graffy 2004	0.2877	0.374	2.4%	1.33 [0.64, 2.78]
Kools 2005	0.1233	0.117	3.0%	1.13 [0.90, 1.42]
Long 1995	-0.6524	0.3887	2.4%	0.52 [0.24, 1.12]
MacArthur 2009	-0.0267	0.0607	3.1%	0.97 [0.86, 1.10]
Mottl-Santiago 2008	-0.3556	0.0205	3.1%	0.70 [0.67, 0.73]
Muirhead 2006	-0.0296	0.1439	3.0%	0.97 [0.73, 1.29]
Nommsen-Rivers 2009	-0.1005	0.2347	2.8%	0.90 [0.57, 1.43]
Olson 2008	-0.559	0.0962	3.0%	0.57 [0.47, 0.69]
Pobocik 2000	-0.4218	0.1627	2.9%	0.66 [0.48, 0.90]
Quinlivan 2003	0.1349	0.2882	2.6%	1.14 [0.65, 2.01]
Ryan 2006	0.6482	0.0064	3.1%	1.91 [1.89, 1.94]
Sandy 2009	-0.4515	0.2844	2.7%	0.64 [0.36, 1.11]
Shaw 1999	-0.0313	0.0715	3.1%	0.97 [0.84, 1.11]
Su 2007a	-0.8179	0.6793	1.6%	0.44 [0.12, 1.67]
Su 2007b	-0.1542	0.5432	1.9%	0.86 [0.30, 2.49]
Wambach 2011	-0.5132	0.2467	2.8%	0.60 [0.37, 0.97]
**Subtotal (95% CI)**			**49.2%**	**0.81 [0.58, 1.12]**
Heterogeneity: Tau² = 0.45; Chi² = 2618.89, df = 18 (P < 0.00001); I² = 99%				
Test for overall effect: Z = 1.27 (P = 0.20)				
**Total (95% CI)**			**100.0%**	**0.68 [0.54, 0.87]**
Heterogeneity: Tau² = 0.48; Chi² = 5901.19, df = 37 (P < 0.00001); I² = 99%				
Test for overall effect: Z = 3.10 (P = 0.002)				
Test for subgroup differences: Chi² = 4.74, df = 2 (P = 0.09), I² = 57.8%				

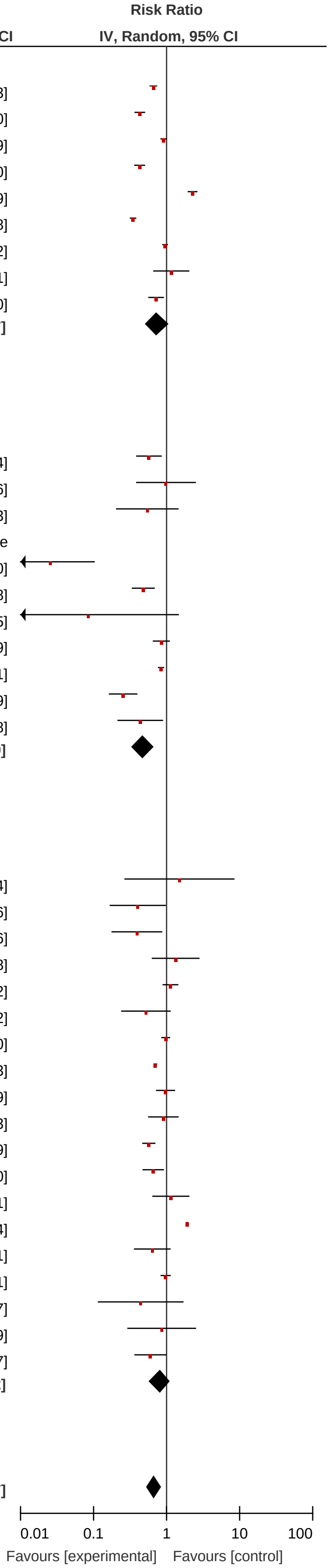

Figure 9c. Effect of breastfeeding education on the rate of no breastfeeding at day 1: developing vs developed countries.

Study or Subgroup	log[Risk Ratio]	SE	Weight	Risk Ratio IV, Random, 95% CI
**1.60.1 developing countries**				
Aksu 2011	0.4055	0.8756	1.2%	1.50 [0.27, 8.34]
Bhandari 2003	-0.4153	0.0529	3.1%	0.66 [0.60, 0.73]
Bhutta 2008	-0.8445	0.0751	3.1%	0.43 [0.37, 0.50]
Dearden 2002	-0.0939	0.0405	3.1%	0.91 [0.84, 0.99]
Haider 2000	-0.8476	0.0775	3.1%	0.43 [0.37, 0.50]
Kumar 2008	-1.0571	0.0418	3.1%	0.35 [0.32, 0.38]
Morrow 1999	0.1476	0.2808	2.7%	1.16 [0.67, 2.01]
Taddei 2000	-0.1755	0.0399	3.1%	0.84 [0.78, 0.91]
Turan 2003	-0.3299	0.1157	3.0%	0.72 [0.57, 0.90]
Valdes 1993	-1.3704	0.2194	2.8%	0.25 [0.17, 0.39]
**Subtotal (95% CI)**			**28.2%**	**0.58 [0.44, 0.78]**
Heterogeneity: Tau² = 0.19; Chi² = 411.45, df = 9 (P < 0.00001); I² = 98%				
Test for overall effect: Z = 3.61 (P = 0.0003)				
**1.60.2 developed countries**				
Anderson 2005	-0.9079	0.4424	2.2%	0.40 [0.17, 0.96]
Brent 1995	-0.5566	0.1962	2.9%	0.57 [0.39, 0.84]
Chapman 2004	-0.9361	0.3992	2.3%	0.39 [0.18, 0.86]
Graffy 2004	0.2877	0.374	2.4%	1.33 [0.64, 2.78]
Ingram 2005	0.8197	0.0683	3.1%	2.27 [1.99, 2.59]
Kools 2005	0.1233	0.117	3.0%	1.13 [0.90, 1.42]
Long 1995	-0.6524	0.3887	2.4%	0.52 [0.24, 1.12]
MacArthur 2009	-0.0267	0.0607	3.1%	0.97 [0.86, 1.10]
Martens 2000	-0.0202	0.4706	2.1%	0.98 [0.39, 2.46]
McInnes 2000	-0.0469	0.0346	3.1%	0.95 [0.89, 1.02]
Mottl-Santiago 2008	-0.3556	0.0205	3.1%	0.70 [0.67, 0.73]
Muirhead 2006	-0.0296	0.1439	3.0%	0.97 [0.73, 1.29]
Nommsen-Rivers 2009	-0.1005	0.2347	2.8%	0.90 [0.57, 1.43]
Olson 2008	-0.559	0.0962	3.0%	0.57 [0.47, 0.69]
Pisacane 2005	-0.6061	0.4932	2.1%	0.55 [0.21, 1.43]
Pobocik 2000	-0.4218	0.1627	2.9%	0.66 [0.48, 0.90]
Quinlivan 2003	0.1349	0.2882	2.6%	1.14 [0.65, 2.01]
Rasmussen 2011	0	0		Not estimable
Rishel 2005	-3.6636	0.7049	1.5%	0.03 [0.01, 0.10]
Rossiter 1994	-0.735	0.1757	2.9%	0.48 [0.34, 0.68]
Ryan 2006	0.6482	0.0064	3.1%	1.91 [1.89, 1.94]
Sandy 2009	-0.4515	0.2844	2.7%	0.64 [0.36, 1.11]
Sciacca 1995	-2.4596	1.4434	0.6%	0.09 [0.01, 1.45]
Serwint 1996	-0.1656	0.1276	3.0%	0.85 [0.66, 1.09]
Shaw 1999	-0.0313	0.0715	3.1%	0.97 [0.84, 1.11]
Su 2007a	-0.8179	0.6793	1.6%	0.44 [0.12, 1.67]
Su 2007b	-0.1542	0.5432	1.9%	0.86 [0.30, 2.49]
Wambach 2011	-0.5132	0.2467	2.8%	0.60 [0.37, 0.97]
Wolfberg 2004	-0.8286	0.3567	2.5%	0.44 [0.22, 0.88]
**Subtotal (95% CI)**			**71.8%**	**0.73 [0.57, 0.95]**
Heterogeneity: Tau² = 0.39; Chi² = 3086.11, df = 27 (P < 0.00001); I² = 99%				
Test for overall effect: Z = 2.37 (P = 0.02)				
**Total (95% CI)**			**100.0%**	**0.68 [0.54, 0.87]**
Heterogeneity: Tau² = 0.48; Chi² = 5901.19, df = 37 (P < 0.00001); I² = 99%				
Test for overall effect: Z = 3.10 (P = 0.002)				
Test for subgroup differences: Chi² = 1.29, df = 1 (P = 0.26), I² = 22.6%				

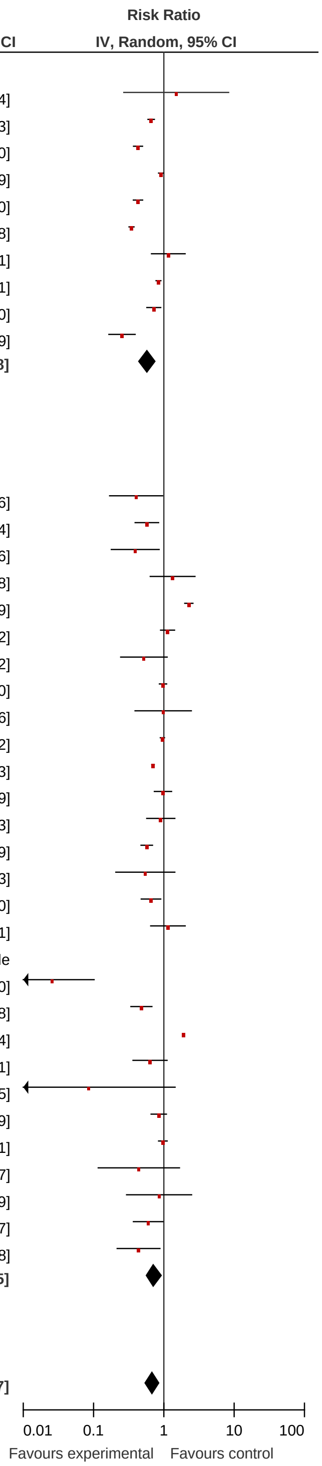

Figure 10a. Effect of breastfeeding education on the rate of no breastfeeding at 0-1 month: individual vs group counseling.

Study or Subgroup	log[Risk Ratio]	SE	Weight	Risk Ratio IV, Random, 95% CI
**1.62.4 individual and group counseling**				
Alvarado 1996	-1.0371	1.6235	0.2%	0.35 [0.01, 8.54]
Gross 1998a	-0.3365	0.3699	2.2%	0.71 [0.35, 1.47]
Gross 1998b	-0.5064	0.395	2.0%	0.60 [0.28, 1.31]
Gross 1998c	-0.8473	0.4364	1.7%	0.43 [0.18, 1.01]
Lieu 1998	-0.2763	0.173	4.6%	0.76 [0.54, 1.06]
Sciacca 1995	-2.4558	1.002	0.4%	0.09 [0.01, 0.61]
Su 2007b	-0.229	0.489	1.5%	0.80 [0.31, 2.07]
**Subtotal (95% CI)**			**12.6%**	**0.66 [0.51, 0.87]**
Heterogeneity: Tau² = 0.00; Chi² = 6.15, df = 6 (P = 0.41); I² = 2%				
Test for overall effect: Z = 2.98 (P = 0.003)				
**1.62.5 individual counseling**				
Bonuck 2006	-0.9695	0.2397	3.6%	0.38 [0.24, 0.61]
Brent 1995	-0.4431	0.4431	1.7%	0.64 [0.27, 1.53]
Bunik 2010	-0.0009	0.1825	4.5%	1.00 [0.70, 1.43]
Chapman 2004	-0.3646	0.1915	4.3%	0.69 [0.48, 1.01]
Dennis 2002	-0.7557	0.3666	2.2%	0.47 [0.23, 0.96]
Gill 2007	-0.3567	0.2359	3.7%	0.70 [0.44, 1.11]
Huang 2007	-0.9808	0.4425	1.7%	0.38 [0.16, 0.89]
Jones 1985	-0.5515	0.1727	4.6%	0.58 [0.41, 0.81]
Kupratakul 2010	-2.2472	1.4738	0.2%	0.11 [0.01, 1.90]
Labarere 2005	-0.5419	0.3365	2.5%	0.58 [0.30, 1.12]
Labarere 2011	0.0095	0.2423	3.6%	1.01 [0.63, 1.62]
Lieu 2000	-0.1731	0.1252	5.4%	0.84 [0.66, 1.07]
Long 1995	-0.4075	0.268	3.2%	0.67 [0.39, 1.12]
McQueen 2011	-0.6007	0.3642	2.3%	0.55 [0.27, 1.12]
Mongeon 1995	0.47	0.2475	3.5%	1.60 [0.99, 2.60]
Muirhead 2006	-0.0061	0.1109	5.6%	0.99 [0.80, 1.24]
Porteous 2000	-2.871	1.429	0.2%	0.06 [0.00, 0.93]
Quinlivan 2003	0.0499	0.1996	4.2%	1.05 [0.71, 1.55]
Rasmussen 2011	1.1499	0.751	0.7%	3.16 [0.72, 13.76]
Ryser 2004	-1.1497	0.4297	1.8%	0.32 [0.14, 0.74]
Schafer 1998	-0.7056	0.1514	5.0%	0.49 [0.37, 0.66]
Serwint 1996	-0.0556	0.0744	6.2%	0.95 [0.82, 1.09]
Simonetti 2012	-1.0296	1.6224	0.2%	0.36 [0.01, 8.59]
Sjolin 1979	-0.5754	0.3847	2.1%	0.56 [0.26, 1.20]
Su 2007a	-1.4435	0.772	0.7%	0.24 [0.05, 1.07]
Tahir 2012	-0.268	0.4071	1.9%	0.76 [0.34, 1.70]
**Subtotal (95% CI)**			**75.5%**	**0.71 [0.61, 0.84]**
Heterogeneity: Tau² = 0.08; Chi² = 70.15, df = 25 (P < 0.00001); I² = 64%				
Test for overall effect: Z = 4.15 (P < 0.0001)				
**1.62.6 group counseling**				
Kang 2007	-0.8267	0.3724	2.2%	0.44 [0.21, 0.91]
Rossiter 1994	-0.3964	0.1195	5.5%	0.67 [0.53, 0.85]
Wolfberg 2004	-0.0473	0.2044	4.1%	0.95 [0.64, 1.42]
**Subtotal (95% CI)**			**11.8%**	**0.71 [0.51, 0.99]**
Heterogeneity: Tau² = 0.04; Chi² = 3.98, df = 2 (P = 0.14); I² = 50%				
Test for overall effect: Z = 2.05 (P = 0.04)				
**Total (95% CI)**			**100.0%**	**0.70 [0.62, 0.80]**
Heterogeneity: Tau² = 0.07; Chi² = 83.03, df = 35 (P < 0.00001); I² = 58%				
Test for overall effect: Z = 5.28 (P < 0.00001)				
Test for subgroup differences: Chi² = 0.18, df = 2 (P = 0.91), I² = 0%				

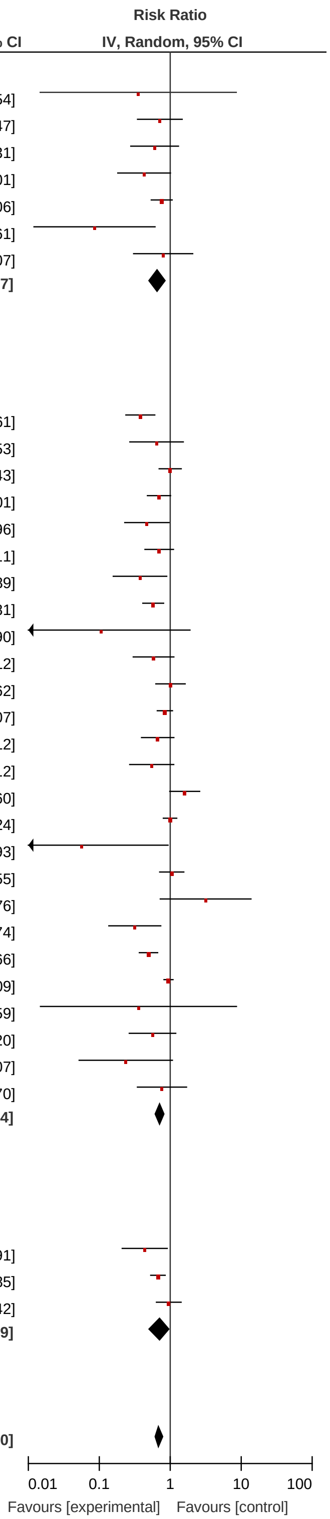

Figure 10b. Effect of breastfeeding education on the rate of no breastfeeding at 0-1 month: community- vs facility-based intervention.

Study or Subgroup	log[Risk Ratio]	SE	Weight	Risk Ratio IV, Random, 95% CI
**1.64.7 community-based intervention**				
Dennis 2002	-0.7557	0.3666	2.2%	0.47 [0.23, 0.96]
Lieu 2000	-0.1731	0.1252	5.4%	0.84 [0.66, 1.07]
Mongeon 1995	0.47	0.2475	3.5%	1.60 [0.99, 2.60]
**Subtotal (95% CI)**			**11.1%**	**0.90 [0.51, 1.57]**
Heterogeneity: Tau² = 0.18; Chi² = 8.84, df = 2 (P = 0.01); I² = 77%				
Test for overall effect: Z = 0.38 (P = 0.70)				
**1.64.8 facility-based intervention**				
Brent 1995	-0.4431	0.4431	1.7%	0.64 [0.27, 1.53]
Huang 2007	-0.9808	0.4425	1.7%	0.38 [0.16, 0.89]
Jones 1985	-0.5515	0.1727	4.6%	0.58 [0.41, 0.81]
Kang 2007	-0.8267	0.3724	2.2%	0.44 [0.21, 0.91]
Kupratakul 2010	-2.2472	1.4738	0.2%	0.11 [0.01, 1.90]
Labarere 2005	-0.5419	0.3365	2.5%	0.58 [0.30, 1.12]
Labarere 2011	0.0095	0.2423	3.6%	1.01 [0.63, 1.62]
Lieu 1998	-0.2763	0.173	4.6%	0.76 [0.54, 1.06]
McQueen 2011	-0.6007	0.3642	2.3%	0.55 [0.27, 1.12]
Rasmussen 2011	1.1499	0.751	0.7%	3.16 [0.72, 13.76]
Rossiter 1994	-0.3964	0.1195	5.5%	0.67 [0.53, 0.85]
Ryser 2004	-1.1497	0.4297	1.8%	0.32 [0.14, 0.74]
Sciacca 1995	-2.4558	1.002	0.4%	0.09 [0.01, 0.61]
Serwint 1996	-0.0556	0.0744	6.2%	0.95 [0.82, 1.09]
Tahir 2012	-0.268	0.4071	1.9%	0.76 [0.34, 1.70]
Wolfberg 2004	-0.0473	0.2044	4.1%	0.95 [0.64, 1.42]
**Subtotal (95% CI)**			**44.0%**	**0.69 [0.56, 0.83]**
Heterogeneity: Tau² = 0.07; Chi² = 35.04, df = 15 (P = 0.002); I² = 57%				
Test for overall effect: Z = 3.77 (P = 0.0002)				
**1.64.9 facility and community-based intervention**				
Alvarado 1996	-1.0371	1.6235	0.2%	0.35 [0.01, 8.54]
Bonuck 2006	-0.9695	0.2397	3.6%	0.38 [0.24, 0.61]
Bunik 2010	-0.0009	0.1825	4.5%	1.00 [0.70, 1.43]
Chapman 2004	-0.3646	0.1915	4.3%	0.69 [0.48, 1.01]
Gill 2007	-0.3567	0.2359	3.7%	0.70 [0.44, 1.11]
Gross 1998a	-0.3365	0.3699	2.2%	0.71 [0.35, 1.47]
Gross 1998b	-0.5064	0.395	2.0%	0.60 [0.28, 1.31]
Gross 1998c	-0.8473	0.4364	1.7%	0.43 [0.18, 1.01]
Long 1995	-0.4075	0.268	3.2%	0.67 [0.39, 1.12]
Muirhead 2006	-0.0061	0.1109	5.6%	0.99 [0.80, 1.24]
Porteous 2000	-2.871	1.429	0.2%	0.06 [0.00, 0.93]
Quinlivan 2003	0.0499	0.1996	4.2%	1.05 [0.71, 1.55]
Schafer 1998	-0.7056	0.1514	5.0%	0.49 [0.37, 0.66]
Simonetti 2012	-1.0296	1.6224	0.2%	0.36 [0.01, 8.59]
Sjolin 1979	-0.5754	0.3847	2.1%	0.56 [0.26, 1.20]
Su 2007a	-1.4435	0.772	0.7%	0.24 [0.05, 1.07]
Su 2007b	-0.229	0.489	1.5%	0.80 [0.31, 2.07]
**Subtotal (95% CI)**			**44.9%**	**0.67 [0.54, 0.82]**
Heterogeneity: Tau² = 0.08; Chi² = 36.31, df = 16 (P = 0.003); I² = 56%				
Test for overall effect: Z = 3.81 (P = 0.0001)				
**Total (95% CI)**			**100.0%**	**0.70 [0.62, 0.80]**
Heterogeneity: Tau² = 0.07; Chi² = 83.03, df = 35 (P < 0.00001); I² = 58%				
Test for overall effect: Z = 5.28 (P < 0.00001)				
Test for subgroup differences: Chi² = 0.95, df = 2 (P = 0.62), I² = 0%				

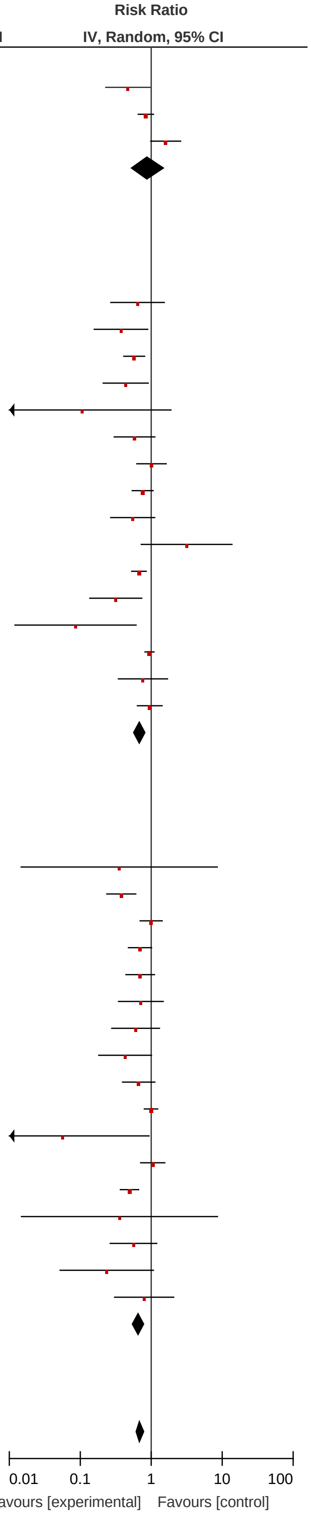

Figure 10c. Effect of breastfeeding education on the rate of no breastfeeding at 0-1 month: developing vs developed countries.

Study or Subgroup	log[Risk Ratio]	SE	Weight	Risk Ratio IV, Random, 95% CI
**1.66.1 developing countries**				
Alvarado 1996	-1.0371	1.6235	0.2%	0.35 [0.01, 8.54]
Huang 2007	-0.9808	0.4425	1.7%	0.38 [0.16, 0.89]
Kupratakul 2010	-2.2472	1.4738	0.2%	0.11 [0.01, 1.90]
Tahir 2012	-0.268	0.4071	1.9%	0.76 [0.34, 1.70]
**Subtotal (95% CI)**			**4.0%**	**0.51 [0.29, 0.90]**
Heterogeneity: Tau² = 0.00; Chi² = 2.66, df = 3 (P = 0.45); I² = 0%				
Test for overall effect: Z = 2.33 (P = 0.02)				
**1.66.2 developed countries**				
Bonuck 2006	-0.9695	0.2397	3.6%	0.38 [0.24, 0.61]
Brent 1995	-0.4431	0.4431	1.7%	0.64 [0.27, 1.53]
Bunik 2010	-0.0009	0.1825	4.5%	1.00 [0.70, 1.43]
Chapman 2004	-0.3646	0.1915	4.3%	0.69 [0.48, 1.01]
Dennis 2002	-0.7557	0.3666	2.2%	0.47 [0.23, 0.96]
Gill 2007	-0.3567	0.2359	3.7%	0.70 [0.44, 1.11]
Gross 1998a	-0.3365	0.3699	2.2%	0.71 [0.35, 1.47]
Gross 1998b	-0.5064	0.395	2.0%	0.60 [0.28, 1.31]
Gross 1998c	-0.8473	0.4364	1.7%	0.43 [0.18, 1.01]
Jones 1985	-0.5515	0.1727	4.6%	0.58 [0.41, 0.81]
Kang 2007	-0.8267	0.3724	2.2%	0.44 [0.21, 0.91]
Labarere 2005	-0.5419	0.3365	2.5%	0.58 [0.30, 1.12]
Labarere 2011	0.0095	0.2423	3.6%	1.01 [0.63, 1.62]
Lieu 1998	-0.2763	0.173	4.6%	0.76 [0.54, 1.06]
Lieu 2000	-0.1731	0.1252	5.4%	0.84 [0.66, 1.07]
Long 1995	-0.4075	0.268	3.2%	0.67 [0.39, 1.12]
McQueen 2011	-0.6007	0.3642	2.3%	0.55 [0.27, 1.12]
Mongeon 1995	0.47	0.2475	3.5%	1.60 [0.99, 2.60]
Muirhead 2006	-0.0061	0.1109	5.6%	0.99 [0.80, 1.24]
Porteous 2000	-2.871	1.429	0.2%	0.06 [0.00, 0.93]
Quinlivan 2003	0.0499	0.1996	4.2%	1.05 [0.71, 1.55]
Rasmussen 2011	1.1499	0.751	0.7%	3.16 [0.72, 13.76]
Rossiter 1994	-0.3964	0.1195	5.5%	0.67 [0.53, 0.85]
Ryser 2004	-1.1497	0.4297	1.8%	0.32 [0.14, 0.74]
Schafer 1998	-0.7056	0.1514	5.0%	0.49 [0.37, 0.66]
Sciacca 1995	-2.4558	1.002	0.4%	0.09 [0.01, 0.61]
Serwint 1996	-0.0556	0.0744	6.2%	0.95 [0.82, 1.09]
Simonetti 2012	-1.0296	1.6224	0.2%	0.36 [0.01, 8.59]
Sjolin 1979	-0.5754	0.3847	2.1%	0.56 [0.26, 1.20]
Su 2007a	-1.4435	0.772	0.7%	0.24 [0.05, 1.07]
Su 2007b	-0.229	0.489	1.5%	0.80 [0.31, 2.07]
Wolfberg 2004	-0.0473	0.2044	4.1%	0.95 [0.64, 1.42]
**Subtotal (95% CI)**			**96.0%**	**0.71 [0.62, 0.81]**
Heterogeneity: Tau² = 0.07; Chi² = 78.19, df = 31 (P < 0.00001); I² = 60%				
Test for overall effect: Z = 4.96 (P < 0.00001)				
**Total (95% CI)**			**100.0%**	**0.70 [0.62, 0.80]**
Heterogeneity: Tau² = 0.07; Chi² = 83.03, df = 35 (P < 0.00001); I² = 58%				
Test for overall effect: Z = 5.28 (P < 0.00001)				
Test for subgroup differences: Chi² = 1.25, df = 1 (P = 0.26), I² = 20.3%				

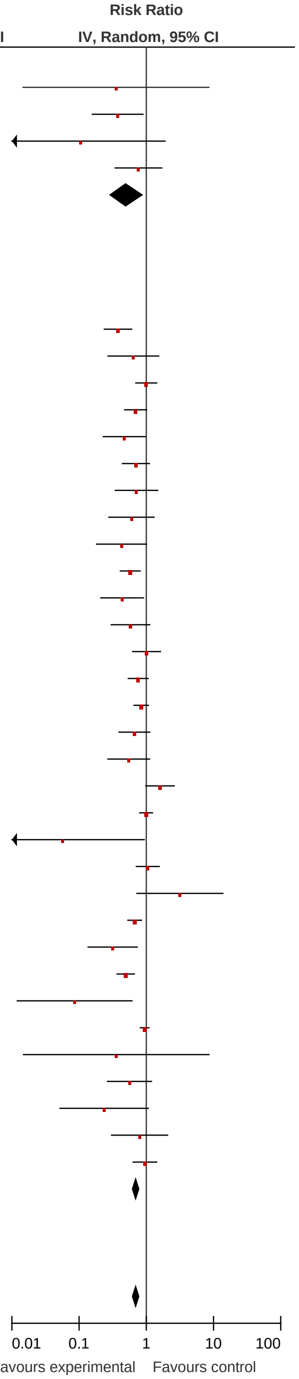

Figure 11a. Effect of breastfeeding education on the rate of no breastfeeding at 1-6 months: individual vs group counseling.

Study or Subgroup	log[Risk Ratio]	SE	Weight	Risk Ratio IV, Random, 95% CI	Risk Ratio IV, Random, 95% CI
**1.68.4 individual counseling**					
Anderson 2005	-0.2294	0.1524	1.4%	0.80 [0.59, 1.07]	
Bashour 2008a	-0.763	0.5948	0.3%	0.47 [0.15, 1.50]	
Bashour 2008b	-0.0036	0.4637	0.5%	1.00 [0.40, 2.47]	
Bonuck 2006	-0.1843	0.1028	1.5%	0.83 [0.68, 1.02]	
Brent 1995	-0.0749	0.0667	1.7%	0.93 [0.81, 1.06]	
Bunik 2010	0.1378	0.0755	1.6%	1.15 [0.99, 1.33]	
Chapman 2004	-0.3075	0.1319	1.4%	0.74 [0.57, 0.95]	
Dennis 2002	-0.5572	0.2208	1.1%	0.57 [0.37, 0.88]	
Frank 1987a	-0.0386	0.1714	1.3%	0.96 [0.69, 1.35]	
Frank 1987b	-0.3559	0.2215	1.1%	0.70 [0.45, 1.08]	
Froozani 1999	-1.2659	0.6252	0.3%	0.28 [0.08, 0.96]	
Gill 2007	-0.3205	0.1142	1.5%	0.73 [0.58, 0.91]	
Graffy 2004	-0.0694	0.0716	1.6%	0.93 [0.81, 1.07]	
Grossman 1990	-0.1037	0.1004	1.6%	0.90 [0.74, 1.10]	
Henderson 2001	0.2007	0.2017	1.2%	1.22 [0.82, 1.81]	
Huang 2007	-0.734	0.3	0.8%	0.48 [0.27, 0.86]	
Johnston 2006a	-0.764	0.2535	1.0%	0.47 [0.28, 0.77]	
Johnston 2006b	-0.5304	0.2309	1.0%	0.59 [0.37, 0.93]	
Jones 1985	-0.1505	0.0611	1.7%	0.86 [0.76, 0.97]	
Khresheh 2011	0.0953	0.2259	1.1%	1.10 [0.71, 1.71]	
Kools 2005	0.0806	0.0559	1.7%	1.08 [0.97, 1.21]	
Kramer 2001	-0.2414	0.0135	1.8%	0.79 [0.77, 0.81]	
Kupratakul 2010	-1.1499	0.2864	0.9%	0.32 [0.18, 0.56]	
Labarere 2003	0.0925	0.1121	1.5%	1.10 [0.88, 1.37]	
Lieu 2000	-0.0654	0.0679	1.7%	0.94 [0.82, 1.07]	
Long 1995	0.1372	0.1203	1.5%	1.15 [0.91, 1.45]	
Lynch 1986	0.025	0.1009	1.6%	1.03 [0.84, 1.25]	
McDonald 2008	0.1192	0.0962	1.6%	1.13 [0.93, 1.36]	
McInnes 2000	-0.0293	0.0208	1.7%	0.97 [0.93, 1.01]	
McQueen 2011	-0.1489	0.2559	1.0%	0.86 [0.52, 1.42]	
Mongeon 1995	-0.0513	0.0752	1.6%	0.95 [0.82, 1.10]	
Morrell 2000	0.0075	0.0456	1.7%	1.01 [0.92, 1.10]	
Morrow 1999	-0.5935	0.4416	0.5%	0.55 [0.23, 1.31]	
Muirhead 2006	-0.0694	0.0678	1.7%	0.93 [0.82, 1.07]	
Nommsen-Rivers 2009	-0.3528	0.2629	0.9%	0.70 [0.42, 1.18]	
Olson 2008	-0.0619	0.0271	1.7%	0.94 [0.89, 0.99]	
Pugh 2002	-0.1288	0.2503	1.0%	0.88 [0.54, 1.44]	
Pugh 2009	-0.0146	0.07	1.7%	0.99 [0.86, 1.13]	
Quinlivan 2003	0.0272	0.0955	1.6%	1.03 [0.85, 1.24]	
Rasmussen 2011	0.5368	0.3151	0.8%	1.71 [0.92, 3.17]	
Rishel 2005	-0.2757	0.4224	0.5%	0.76 [0.33, 1.74]	
Ryan 2006	0.3211	0.0106	1.8%	1.38 [1.35, 1.41]	
Schafer 1998	-0.5407	0.1075	1.5%	0.58 [0.47, 0.72]	
Schlickau 2005a	-0.069	0.3357	0.7%	0.93 [0.48, 1.80]	
Schlickau 2005b	-0.4745	0.4428	0.5%	0.62 [0.26, 1.48]	
Serwint 1996	-0.0248	0.0546	1.7%	0.98 [0.88, 1.09]	
Shaw 1999	-0.1618	0.0585	1.7%	0.85 [0.76, 0.95]	
Simonetti 2012	-0.4067	0.2343	1.0%	0.67 [0.42, 1.05]	
Sjolin 1979	-0.1706	0.1312	1.4%	0.84 [0.65, 1.09]	
Su 2007a	-0.099	0.099	1.6%	0.91 [0.75, 1.10]	
Tahir 2012	-0.3913	0.296	0.8%	0.68 [0.38, 1.21]	
Valdes 1993	-0.7826	0.1742	1.3%	0.46 [0.32, 0.64]	
Vitolo 2005	-0.3518	0.1414	1.4%	0.70 [0.53, 0.93]	
Wallace 2006	0.0409	0.0855	1.6%	1.04 [0.88, 1.23]	
Wen 2011	-0.1596	0.06	1.7%	0.85 [0.76, 0.96]	
Wilhelm 2006	-0.1044	0.1491	1.4%	0.90 [0.67, 1.21]	
Wrenn 1997	-0.1426	0.2476	1.0%	0.87 [0.53, 1.41]	
**Subtotal (95% CI)**			**73.0%**	**0.86 [0.79, 0.94]**	

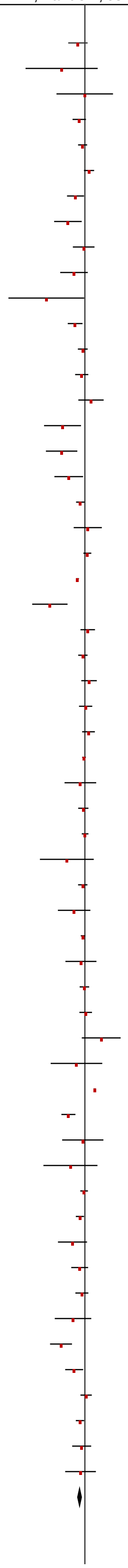

Heterogeneity: Tau² = 0.07; Chi² = 1378.90, df = 56 (P < 0.00001); I² = 96%

Test for overall effect: Z = 3.49 (P = 0.0005)

**1.68.5 group counseling**				
Forster 2004a	-0.008	0.0894	1.6%	0.99 [0.83, 1.18]
Forster 2004b	0.0907	0.0857	1.6%	1.09 [0.93, 1.30]
Garcia-Montrone 1996	-0.47	0.3536	0.7%	0.63 [0.31, 1.25]
Hill 1987	-0.1285	0.1832	1.2%	0.88 [0.61, 1.26]
Kang 2007	-0.6931	0.2415	1.0%	0.50 [0.31, 0.80]
Kruske 2007	-1.3519	0.3066	0.8%	0.26 [0.14, 0.47]
Noel-Weiss 2006	-0.4002	0.4465	0.5%	0.67 [0.28, 1.61]
Reifsnider 1997	-0.2758	0.1796	1.2%	0.76 [0.53, 1.08]
Rossiter 1994	-0.1207	0.0778	1.6%	0.89 [0.76, 1.03]
Turan 2003	-0.7428	0.3375	0.7%	0.48 [0.25, 0.92]
Wolfberg 2004	-0.2098	0.1676	1.3%	0.81 [0.58, 1.13]
**Subtotal (95% CI)**			**12.2%**	**0.76 [0.63, 0.91]**
Heterogeneity: Tau² = 0.05; Chi² = 35.21, df = 10 (P = 0.0001); I² = 72%				
Test for overall effect: Z = 2.96 (P = 0.003)				
**1.68.6 individual and group counseling**				
Alvarado 1996	-3.1564	1.0044	0.1%	0.04 [0.01, 0.30]
Bhandari 2003	1.2273	0.7877	0.2%	3.41 [0.73, 15.98]
Gross 1998a	-0.6217	0.1895	1.2%	0.54 [0.37, 0.78]
Gross 1998b	-0.6993	0.2189	1.1%	0.50 [0.32, 0.76]
Gross 1998c	-0.4798	0.1563	1.3%	0.62 [0.46, 0.84]
Ingram 2005	0.4406	0.0404	1.7%	1.55 [1.44, 1.68]
Lovera 2010	-0.3201	0.1534	1.3%	0.73 [0.54, 0.98]
Pobocik 2000	-0.2013	0.0656	1.7%	0.82 [0.72, 0.93]
Redman 1995	0.0155	0.1	1.6%	1.02 [0.83, 1.24]
Rosen 2008	-0.4797	0.163	1.3%	0.62 [0.45, 0.85]
Sciacca 1995	-0.6793	0.2693	0.9%	0.51 [0.30, 0.86]
Su 2007b	-0.1381	0.101	1.6%	0.87 [0.71, 1.06]
Valdes 2000	-1.4087	0.3058	0.8%	0.24 [0.13, 0.45]
**Subtotal (95% CI)**			**14.8%**	**0.68 [0.51, 0.91]**
Heterogeneity: Tau² = 0.22; Chi² = 205.09, df = 12 (P < 0.00001); I² = 94%				
Test for overall effect: Z = 2.59 (P = 0.010)				
**Total (95% CI)**			**100.0%**	**0.82 [0.77, 0.89]**
Heterogeneity: Tau² = 0.08; Chi² = 1635.68, df = 80 (P < 0.00001); I² = 95%				
Test for overall effect: Z = 5.26 (P < 0.00001)				
Test for subgroup differences: Chi² = 3.53, df = 2 (P = 0.17), I² = 43.3%				

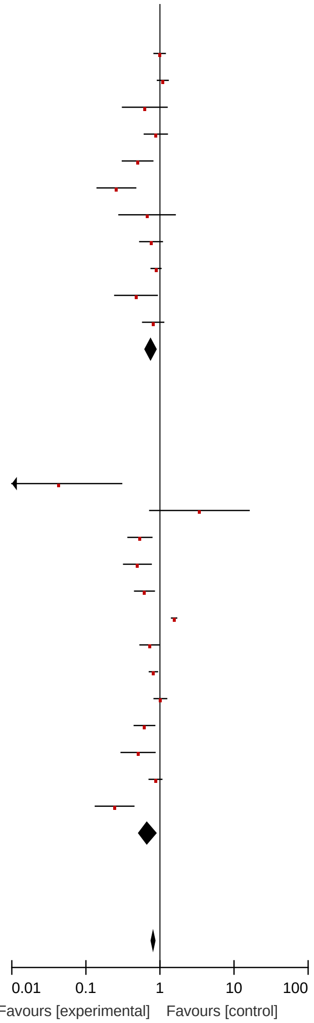

Figure 11b. Effect of breastfeeding education on the rate of no breastfeeding at 1-6 months: community- vs facility-based intervention.

Study or Subgroup	log[Risk Ratio]	SE	Weight	Risk Ratio IV, Random, 95% CI
**1.70.1 community-based intervention**				
Bashour 2008a	-0.763	0.5948	0.3%	0.47 [0.15, 1.50]
Bashour 2008b	-0.0036	0.4637	0.5%	1.00 [0.40, 2.47]
Bhandari 2003	1.2273	0.7877	0.2%	3.41 [0.73, 15.98]
Dennis 2002	-0.5572	0.2208	1.1%	0.57 [0.37, 0.88]
Ingram 2005	0.4406	0.0404	1.7%	1.55 [1.44, 1.68]
Kruske 2007	-1.3519	0.3066	0.8%	0.26 [0.14, 0.47]
Lieu 2000	-0.0654	0.0679	1.7%	0.94 [0.82, 1.07]
Lynch 1986	0.025	0.1009	1.6%	1.03 [0.84, 1.25]
McInnes 2000	-0.0293	0.0208	1.7%	0.97 [0.93, 1.01]
Mongeon 1995	-0.0513	0.0752	1.6%	0.95 [0.82, 1.10]
Morrell 2000	0.0075	0.0456	1.7%	1.01 [0.92, 1.10]
Morrow 1999	-0.5935	0.4416	0.5%	0.55 [0.23, 1.31]
Turan 2003	-0.7428	0.3375	0.7%	0.48 [0.25, 0.92]
**Subtotal (95% CI)**			**14.1%**	**0.89 [0.75, 1.06]**
Heterogeneity: Tau² = 0.06; Chi² = 153.29, df = 12 (P < 0.00001); I² = 92%				
Test for overall effect: Z = 1.34 (P = 0.18)				
**1.70.2 facility-based intervention**				
Brent 1995	-0.0749	0.0667	1.7%	0.93 [0.81, 1.06]
Garcia-Montrone 1996	-0.47	0.3536	0.7%	0.63 [0.31, 1.25]
Grossman 1990	-0.1037	0.1004	1.6%	0.90 [0.74, 1.10]
Henderson 2001	0.2007	0.2017	1.2%	1.22 [0.82, 1.81]
Hill 1987	-0.1285	0.1832	1.2%	0.88 [0.61, 1.26]
Huang 2007	-0.734	0.3	0.8%	0.48 [0.27, 0.86]
Jones 1985	-0.1505	0.0611	1.7%	0.86 [0.76, 0.97]
Kang 2007	-0.6931	0.2415	1.0%	0.50 [0.31, 0.80]
Khresheh 2011	0.0953	0.2259	1.1%	1.10 [0.71, 1.71]
Kramer 2001	-0.2414	0.0135	1.8%	0.79 [0.77, 0.81]
Kupratakul 2010	-1.1499	0.2864	0.9%	0.32 [0.18, 0.56]
Labarere 2003	0.0925	0.1121	1.5%	1.10 [0.88, 1.37]
McDonald 2008	0.1192	0.0962	1.6%	1.13 [0.93, 1.36]
McQueen 2011	-0.1489	0.2559	1.0%	0.86 [0.52, 1.42]
Noel-Weiss 2006	-0.4002	0.4465	0.5%	0.67 [0.28, 1.61]
Pugh 2002	-0.1288	0.2503	1.0%	0.88 [0.54, 1.44]
Rasmussen 2011	0.5368	0.3151	0.8%	1.71 [0.92, 3.17]
Reifsnider 1997	-0.2758	0.1796	1.2%	0.76 [0.53, 1.08]
Rishel 2005	-0.2757	0.4224	0.5%	0.76 [0.33, 1.74]
Rosen 2008	-0.4797	0.163	1.3%	0.62 [0.45, 0.85]
Rossiter 1994	-0.1207	0.0778	1.6%	0.89 [0.76, 1.03]
Schlickau 2005a	-0.069	0.3357	0.7%	0.93 [0.48, 1.80]
Schlickau 2005b	-0.4745	0.4428	0.5%	0.62 [0.26, 1.48]
Sciacca 1995	-0.6793	0.2693	0.9%	0.51 [0.30, 0.86]
Serwint 1996	-0.0248	0.0546	1.7%	0.98 [0.88, 1.09]
Tahir 2012	-0.3913	0.296	0.8%	0.68 [0.38, 1.21]
Valdes 1993	-0.7826	0.1742	1.3%	0.46 [0.32, 0.64]
Valdes 2000	-1.4087	0.3058	0.8%	0.24 [0.13, 0.45]
Wallace 2006	0.0409	0.0855	1.6%	1.04 [0.88, 1.23]
Wilhelm 2006	-0.1044	0.1491	1.4%	0.90 [0.67, 1.21]
Wolfberg 2004	-0.2098	0.1676	1.3%	0.81 [0.58, 1.13]
**Subtotal (95% CI)**			**35.3%**	**0.82 [0.75, 0.90]**
Heterogeneity: Tau² = 0.03; Chi² = 116.01, df = 30 (P < 0.00001); I² = 74%				
Test for overall effect: Z = 4.46 (P < 0.00001)				

Risk Ratio
IV, Random, 95% CI

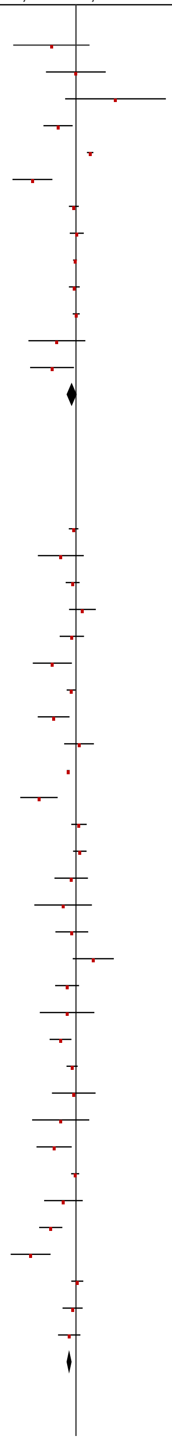

**1.70.3 facility and community-based intervention**				
Alvarado 1996	-3.1564	1.0044	0.1%	0.04 [0.01, 0.30]
Anderson 2005	-0.2294	0.1524	1.4%	0.80 [0.59, 1.07]
Bonuck 2006	-0.1843	0.1028	1.5%	0.83 [0.68, 1.02]
Bunik 2010	0.1378	0.0755	1.6%	1.15 [0.99, 1.33]
Chapman 2004	-0.3075	0.1319	1.4%	0.74 [0.57, 0.95]
Forster 2004a	-0.008	0.0894	1.6%	0.99 [0.83, 1.18]
Forster 2004b	0.0907	0.0857	1.6%	1.09 [0.93, 1.30]
Frank 1987a	-0.0386	0.1714	1.3%	0.96 [0.69, 1.35]
Frank 1987b	-0.3559	0.2215	1.1%	0.70 [0.45, 1.08]
Froozani 1999	-1.2659	0.6252	0.3%	0.28 [0.08, 0.96]
Gill 2007	-0.3205	0.1142	1.5%	0.73 [0.58, 0.91]
Graffy 2004	-0.0694	0.0716	1.6%	0.93 [0.81, 1.07]
Gross 1998a	-0.6217	0.1895	1.2%	0.54 [0.37, 0.78]
Gross 1998b	-0.6993	0.2189	1.1%	0.50 [0.32, 0.76]
Gross 1998c	-0.4798	0.1563	1.3%	0.62 [0.46, 0.84]
Johnston 2006a	-0.764	0.2535	1.0%	0.47 [0.28, 0.77]
Johnston 2006b	-0.5304	0.2309	1.0%	0.59 [0.37, 0.93]
Kools 2005	0.0806	0.0559	1.7%	1.08 [0.97, 1.21]
Long 1995	0.1372	0.1203	1.5%	1.15 [0.91, 1.45]
Lovera 2010	-0.3201	0.1534	1.3%	0.73 [0.54, 0.98]
Muirhead 2006	-0.0694	0.0678	1.7%	0.93 [0.82, 1.07]
Nommsen-Rivers 2009	-0.3528	0.2629	0.9%	0.70 [0.42, 1.18]
Olson 2008	-0.0619	0.0271	1.7%	0.94 [0.89, 0.99]
Pobocik 2000	-0.2013	0.0656	1.7%	0.82 [0.72, 0.93]
Pugh 2009	-0.0146	0.07	1.7%	0.99 [0.86, 1.13]
Quinlivan 2003	0.0272	0.0955	1.6%	1.03 [0.85, 1.24]
Redman 1995	0.0155	0.1	1.6%	1.02 [0.83, 1.24]
Ryan 2006	0.3211	0.0106	1.8%	1.38 [1.35, 1.41]
Schafer 1998	-0.5407	0.1075	1.5%	0.58 [0.47, 0.72]
Shaw 1999	-0.1618	0.0585	1.7%	0.85 [0.76, 0.95]
Simonetti 2012	-0.4067	0.2343	1.0%	0.67 [0.42, 1.05]
Sjolin 1979	-0.1706	0.1312	1.4%	0.84 [0.65, 1.09]
Su 2007a	-0.099	0.099	1.6%	0.91 [0.75, 1.10]
Su 2007b	-0.1381	0.101	1.6%	0.87 [0.71, 1.06]
Vitolo 2005	-0.3518	0.1414	1.4%	0.70 [0.53, 0.93]
Wen 2011	-0.1596	0.06	1.7%	0.85 [0.76, 0.96]
Wrenn 1997	-0.1426	0.2476	1.0%	0.87 [0.53, 1.41]
**Subtotal (95% CI)**			**50.6%**	**0.83 [0.75, 0.92]**

Heterogeneity: $Tau^2 = 0.08$; $Chi^2 = 662.79$, df = 36 ($P < 0.00001$); $I^2 = 95\%$

Test for overall effect: $Z = 3.50$ ($P = 0.0005$)

**Total (95% CI)**			**100.0%**	**0.82 [0.77, 0.89]**

Heterogeneity: $Tau^2 = 0.08$; $Chi^2 = 1635.68$, df = 80 ($P < 0.00001$); $I^2 = 95\%$

Test for overall effect: $Z = 5.26$ ($P < 0.00001$)

Test for subgroup differences: $Chi^2 = 0.63$, df = 2 ($P = 0.73$), $I^2 = 0\%$

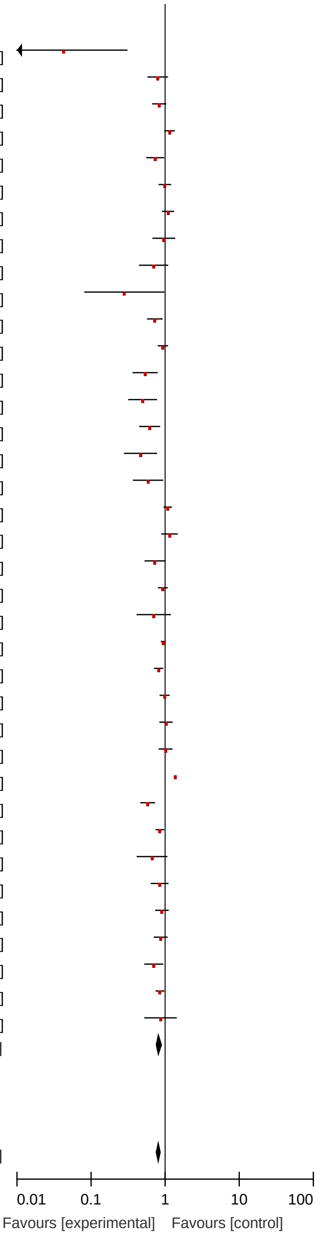

Figure 11c. Effect of breastfeeding education on the rate of no breastfeeding at 1-6 months: developing vs developed countries.

Study or Subgroup	log[Risk Ratio]	SE	Weight	Risk Ratio IV, Random, 95% CI
**1.72.1 developing countries**				
Alvarado 1996	-3.1564	1.0044	0.1%	0.04 [0.01, 0.30]
Bashour 2008a	-0.763	0.5948	0.3%	0.47 [0.15, 1.50]
Bashour 2008b	-0.0036	0.4637	0.5%	1.00 [0.40, 2.47]
Bhandari 2003	1.2273	0.7877	0.2%	3.41 [0.73, 15.98]
Froozani 1999	-1.2659	0.6252	0.3%	0.28 [0.08, 0.96]
Garcia-Montrone 1996	-0.47	0.3536	0.7%	0.63 [0.31, 1.25]
Huang 2007	-0.734	0.3	0.8%	0.48 [0.27, 0.86]
Johnston 2006a	-0.764	0.2535	1.0%	0.47 [0.28, 0.77]
Johnston 2006b	-0.5304	0.2309	1.0%	0.59 [0.37, 0.93]
Khresheh 2011	0.0953	0.2259	1.1%	1.10 [0.71, 1.71]
Kramer 2001	-0.2414	0.0135	1.8%	0.79 [0.77, 0.81]
Kupratakul 2010	-1.1499	0.2864	0.9%	0.32 [0.18, 0.56]
Morrow 1999	-0.5935	0.4416	0.5%	0.55 [0.23, 1.31]
Tahir 2012	-0.3913	0.296	0.8%	0.68 [0.38, 1.21]
Turan 2003	-0.7428	0.3375	0.7%	0.48 [0.25, 0.92]
Valdes 1993	-0.7826	0.1742	1.3%	0.46 [0.32, 0.64]
Valdes 2000	-1.4087	0.3058	0.8%	0.24 [0.13, 0.45]
Vitolo 2005	-0.3518	0.1414	1.4%	0.70 [0.53, 0.93]
**Subtotal (95% CI)**			**14.0%**	**0.56 [0.45, 0.69]**
Heterogeneity: Tau² = 0.11; Chi² = 63.84, df = 17 (P < 0.00001); I² = 73%				
Test for overall effect: Z = 5.32 (P < 0.00001)				
**1.72.2 developed countries**				
Anderson 2005	-0.2294	0.1524	1.4%	0.80 [0.59, 1.07]
Bonuck 2006	-0.1843	0.1028	1.5%	0.83 [0.68, 1.02]
Brent 1995	-0.0749	0.0667	1.7%	0.93 [0.81, 1.06]
Bunik 2010	0.1378	0.0755	1.6%	1.15 [0.99, 1.33]
Chapman 2004	-0.3075	0.1319	1.4%	0.74 [0.57, 0.95]
Dennis 2002	-0.5572	0.2208	1.1%	0.57 [0.37, 0.88]
Forster 2004a	-0.008	0.0894	1.6%	0.99 [0.83, 1.18]
Forster 2004b	0.0907	0.0857	1.6%	1.09 [0.93, 1.30]
Frank 1987a	-0.0386	0.1714	1.3%	0.96 [0.69, 1.35]
Frank 1987b	-0.3559	0.2215	1.1%	0.70 [0.45, 1.08]
Gill 2007	-0.3205	0.1142	1.5%	0.73 [0.58, 0.91]
Graffy 2004	-0.0694	0.0716	1.6%	0.93 [0.81, 1.07]
Gross 1998a	-0.6217	0.1895	1.2%	0.54 [0.37, 0.78]
Gross 1998b	-0.6993	0.2189	1.1%	0.50 [0.32, 0.76]
Gross 1998c	-0.4798	0.1563	1.3%	0.62 [0.46, 0.84]
Grossman 1990	-0.1037	0.1004	1.6%	0.90 [0.74, 1.10]
Henderson 2001	0.2007	0.2017	1.2%	1.22 [0.82, 1.81]
Hill 1987	-0.1285	0.1832	1.2%	0.88 [0.61, 1.26]
Ingram 2005	0.4406	0.0404	1.7%	1.55 [1.44, 1.68]
Jones 1985	-0.1505	0.0611	1.7%	0.86 [0.76, 0.97]
Kang 2007	-0.6931	0.2415	1.0%	0.50 [0.31, 0.80]
Kools 2005	0.0806	0.0559	1.7%	1.08 [0.97, 1.21]
Kruske 2007	-1.3519	0.3066	0.8%	0.26 [0.14, 0.47]
Labarere 2003	0.0925	0.1121	1.5%	1.10 [0.88, 1.37]
Lieu 2000	-0.0654	0.0679	1.7%	0.94 [0.82, 1.07]
Long 1995	0.1372	0.1203	1.5%	1.15 [0.91, 1.45]
Lovera 2010	-0.3201	0.1534	1.3%	0.73 [0.54, 0.98]
Lynch 1986	0.025	0.1009	1.6%	1.03 [0.84, 1.25]
McDonald 2008	0.1192	0.0962	1.6%	1.13 [0.93, 1.36]
McInnes 2000	-0.0293	0.0208	1.7%	0.97 [0.93, 1.01]
McQueen 2011	-0.1489	0.2559	1.0%	0.86 [0.52, 1.42]
Mongeon 1995	-0.0513	0.0752	1.6%	0.95 [0.82, 1.10]
Morrell 2000	0.0075	0.0456	1.7%	1.01 [0.92, 1.10]
Muirhead 2006	-0.0694	0.0678	1.7%	0.93 [0.82, 1.07]
Noel-Weiss 2006	-0.4002	0.4465	0.5%	0.67 [0.28, 1.61]
Nommsen-Rivers 2009	-0.3528	0.2629	0.9%	0.70 [0.42, 1.18]
Olson 2008	-0.0619	0.0271	1.7%	0.94 [0.89, 0.99]
Pobocik 2000	-0.2013	0.0656	1.7%	0.82 [0.72, 0.93]
Pugh 2002	-0.1288	0.2503	1.0%	0.88 [0.54, 1.44]
Pugh 2009	-0.0146	0.07	1.7%	0.99 [0.86, 1.13]
Quinlivan 2003	0.0272	0.0955	1.6%	1.03 [0.85, 1.24]
Rasmussen 2011	0.5368	0.3151	0.8%	1.71 [0.92, 3.17]

Risk Ratio IV, Random, 95% CI

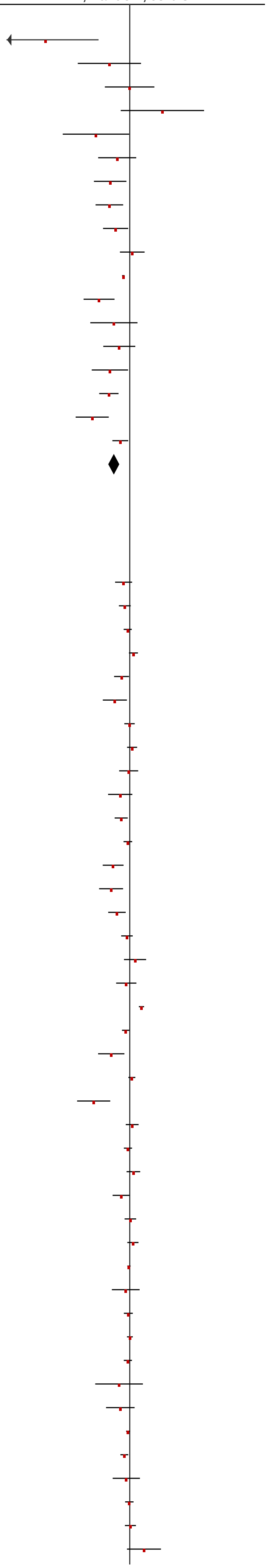

Redman 1995	0.0155	0.1	1.6%	1.02 [0.83, 1.24]
Reifsnider 1997	-0.2758	0.1796	1.2%	0.76 [0.53, 1.08]
Rishel 2005	-0.2757	0.4224	0.5%	0.76 [0.33, 1.74]
Rosen 2008	-0.4797	0.163	1.3%	0.62 [0.45, 0.85]
Rossiter 1994	-0.1207	0.0778	1.6%	0.89 [0.76, 1.03]
Ryan 2006	0.3211	0.0106	1.8%	1.38 [1.35, 1.41]
Schafer 1998	-0.5407	0.1075	1.5%	0.58 [0.47, 0.72]
Schlickau 2005a	-0.069	0.3357	0.7%	0.93 [0.48, 1.80]
Schlickau 2005b	-0.4745	0.4428	0.5%	0.62 [0.26, 1.48]
Sciacca 1995	-0.6793	0.2693	0.9%	0.51 [0.30, 0.86]
Serwint 1996	-0.0248	0.0546	1.7%	0.98 [0.88, 1.09]
Shaw 1999	-0.1618	0.0585	1.7%	0.85 [0.76, 0.95]
Simonetti 2012	-0.4067	0.2343	1.0%	0.67 [0.42, 1.05]
Sjolin 1979	-0.1706	0.1312	1.4%	0.84 [0.65, 1.09]
Su 2007a	-0.099	0.099	1.6%	0.91 [0.75, 1.10]
Su 2007b	-0.1381	0.101	1.6%	0.87 [0.71, 1.06]
Wallace 2006	0.0409	0.0855	1.6%	1.04 [0.88, 1.23]
Wen 2011	-0.1596	0.06	1.7%	0.85 [0.76, 0.96]
Wilhelm 2006	-0.1044	0.1491	1.4%	0.90 [0.67, 1.21]
Wolfberg 2004	-0.2098	0.1676	1.3%	0.81 [0.58, 1.13]
Wrenn 1997	-0.1426	0.2476	1.0%	0.87 [0.53, 1.41]
**Subtotal (95% CI)**			**86.0%**	**0.88 [0.82, 0.95]**

Heterogeneity: Tau² = 0.06; Chi² = 928.57, df = 62 (P < 0.00001); I² = 93%
Test for overall effect: Z = 3.53 (P = 0.0004)

**Total (95% CI)**			**100.0%**	**0.82 [0.77, 0.89]**

Heterogeneity: Tau² = 0.08; Chi² = 1635.68, df = 80 (P < 0.00001); I² = 95%
Test for overall effect: Z = 5.26 (P < 0.00001)
Test for subgroup differences: Chi² = 15.69, df = 1 (P < 0.0001), I² = 93.6%

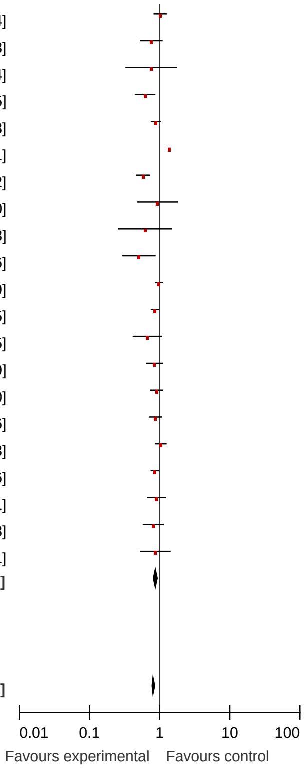

Figure 11d. Effect of breastfeeding education on the rate of no breastfeeding at 6-12 months.

Study or Subgroup	log[Risk Ratio]	SE	Weight	Risk Ratio IV, Random, 95% CI
Bonuck 2006	0.1884	0.3332	0.8%	1.21 [0.63, 2.32]
Jones 1985	0.0328	0.0198	18.5%	1.03 [0.99, 1.07]
Kramer 2001	-0.0983	0.0066	19.9%	0.91 [0.89, 0.92]
Lovera 2010	0	0		Not estimable
Olson 2008	0.0008	0.0028	20.1%	1.00 [1.00, 1.01]
Pisacane 2005	-0.0929	0.0513	12.6%	0.91 [0.82, 1.01]
Quinlivan 2003	0.0272	0.0955	6.5%	1.03 [0.85, 1.24]
Vitolo 2005	-0.2073	0.0997	6.1%	0.81 [0.67, 0.99]
Wen 2011	-0.0758	0.0363	15.5%	0.93 [0.86, 1.00]
**Total (95% CI)**			**100.0%**	**0.95 [0.90, 1.01]**

Heterogeneity: Tau² = 0.00; Chi² = 206.24, df = 7 (P < 0.00001); I² = 97%

Test for overall effect: Z = 1.56 (P = 0.12)

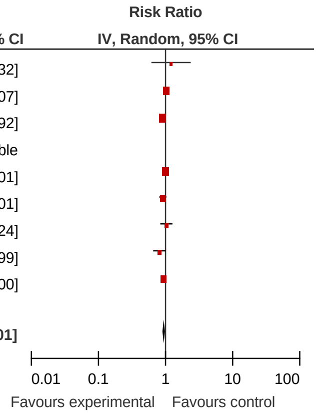

Figure 11e. Effect of breastfeeding education on the rate of no breastfeeding from 12 to 23 months.

Study or Subgroup	log[Risk Ratio]	SE	Weight	Risk Ratio IV, Random, 95% CI
Greiner 1999	-0.3315	0.3706	100.0%	0.72 [0.35, 1.48]
**Total (95% CI)**			**100.0%**	**0.72 [0.35, 1.48]**

Heterogeneity: Not applicable

Test for overall effect: Z = 0.89 (P = 0.37)

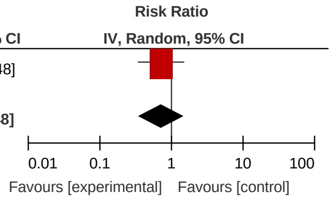

Supplement: Additional File 2 — Plots for meta analyses for day 1 and 0-1 month. [file 1471-2458-13-S3-S20-S2.pdf]
